# Aldehyde or Hydrate? Investigation into the Oxidation of 5‐Formylcytosine Derivatives Using a Computational and Experimental Approach

**DOI:** 10.1002/cbic.202500480

**Published:** 2025-09-23

**Authors:** Kuangjie Liu, Annika Menke, Fabian L. Zott, Domenic Mayer, Lena J. Daumann, Hendrik Zipse

**Affiliations:** ^1^ Faculty of Chemistry and Pharmacy Ludwig‐Maximilians University Munich Butenandtstr. 5‐13 81377 Munich Germany; ^2^ Chair of Bioinorganic Chemistry Heinrich Heine University Düsseldorf Universitätsstraße 1 40225 Düsseldorf Germany

**Keywords:** aldehyde hydrates, C—H bond oxidation, DNA methylation, modified nucleobases, ten‐eleven‐translocation enzymes

## Abstract

This study investigates the oxidation of 5‐hydroxymethyl and 5‐formyl nucleobases using an iron(IV)‐oxido complex that mimics the function of TET enzymes. A central question in this context is whether the oxidation of formyl substrates proceeds via the aldehyde or the hydrate form. To investigate the possible different reaction kinetics of these two forms, nucleobases containing a 6‐aza‐moiety are employed, giving rise to significantly more aldehyde hydrate as compared to the unaltered nucleobase. The concentration changes of substrates and products during oxidation were followed with ^1^H NMR spectroscopy. To analyze the kinetics of the oxidation reactions, a detailed numerical simulation of the stepwise sequential oxidation process is applied. 5‐Hydroxymethyl nucleobases are first oxidized to the respective 5‐formyl derivatives, which exist in equilibrium with their hydrate forms, and then further oxidized to the final 5‐carboxyl nucleobases. The rate constants for 5‐hydroxymethyl nucleobase oxidation show a good correlation with C—H bond dissociation values. The influence of hydrate formation on sequential oxidation is most prominent in the 6‐aza‐derivatives. The results not only deepen our understanding of substrate oxidation by iron‐oxido species but also pave the way for future studies on related biological oxidation mechanisms.

## Graphical abstract

Hydrate‐mediated oxidation of 5‐formyl nucleobases by the iron(IV)‐oxido complex proceeds via geminal diol intermediates, accelerating hydrogen‐atom transfer and revealing aza‐dependent kinetic and mechanistic pathways.

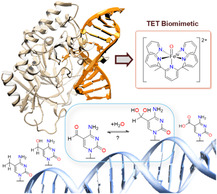

## 1 Introduction

Epigenetics expands the scope of biological information storage beyond the sequence of canonical DNA bases.^[^ ^1^ ^]^ The most prominent epigenetic alteration is the methylation of cytosine (**C**) to 5‐methylcytosine (**5mC**).^[^ ^2^ ^]^ Methylated CpG sites act in the regulation of gene expression by influencing repressor protein^[^ ^3^ ^]^ and transcription factor binding,^[^ ^4^ ^]^ as well as chromatin dynamics.^[^ ^5^ ^]^ The reversibility of DNA modifications enable changes during an organism's development and its adaptation to environmental circumstances.

**Figure 1:**
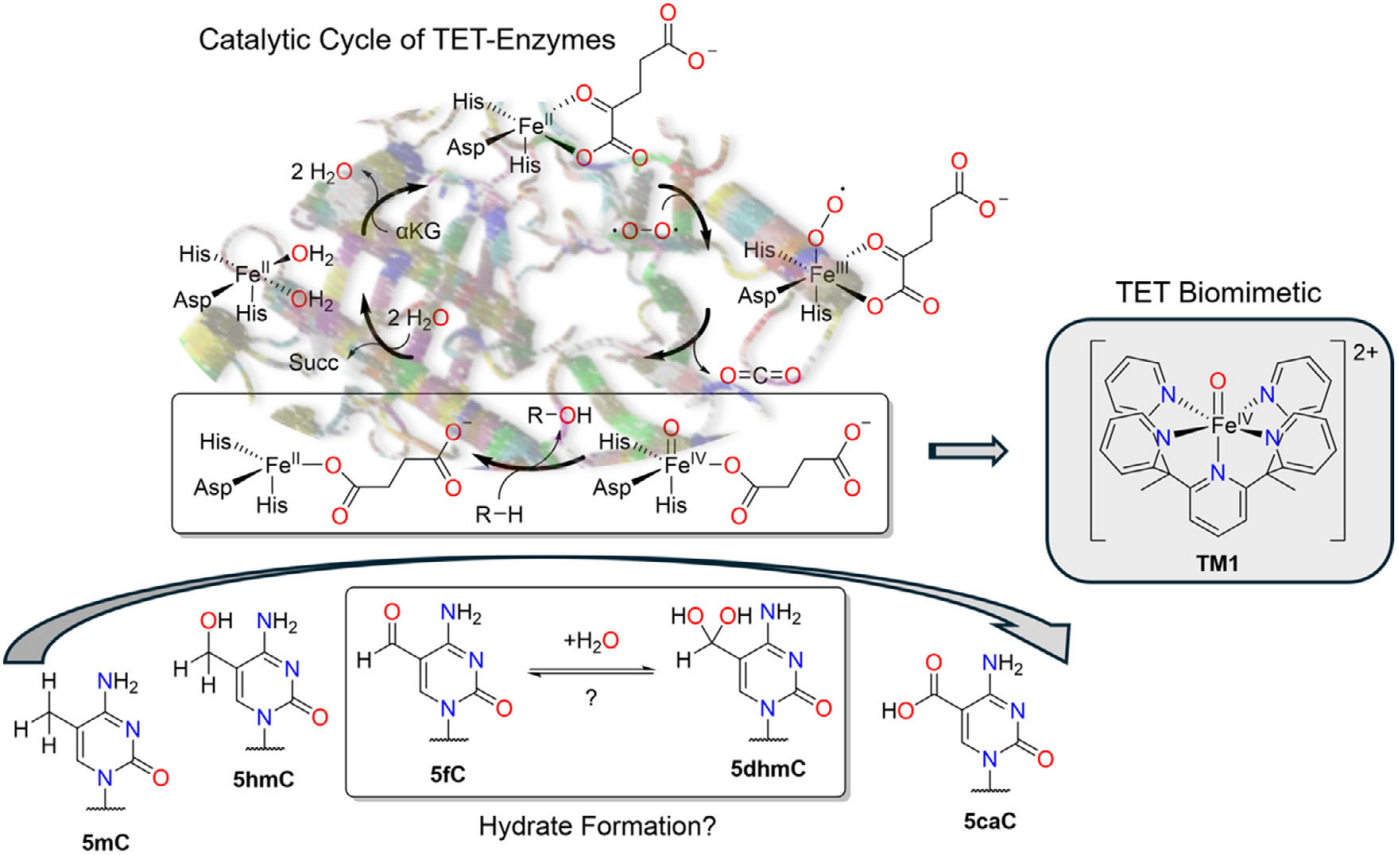
Catalytic cycle of TET enzymes. Highlighted hydrogen atom transfer step (HAT) and sequential array of oxidized substrates from **5mC** to **5caC**. Structure of TET biomimetic (**TM1**) used as model complex.^[^ ^20^ ^]^

To this end, demethylation is largely orchestrated by the *α*‐ketoglutarate‐ and O_2_‐dependent nonheme ten‐eleven translocation (TET) dioxygenases.^[^ ^6^ ^]^ TET enzymes can sequentially oxidize **5mC** to their respective hydroxymethyl (**5hmC**), formyl (**5fC**), and carboxylic acid (**5caC**) derivatives. These moieties are prone to passive demethylation during replication, active demethylation via the base excision repair (BER) pathway, or the less established direct deformylation and decarboxylation.^[^ ^7^ ^]^ In the generally accepted oxidation mechanism of TET enzymes,^[^ ^8^ ^]^ the hydrogen atom transfer (HAT) step is viewed as rate‐limiting, but not all steps are fully elucidated. In particular, a potential hydrate formation of **5fC** toward 5‐dihydroxymethylcytosine (**5dhmC**) is discussed concerning further TET‐dependent oxidation and specific oligonucleotide binding events (**Figure** **1**).^[^ ^9^ ^]^ The potential involvement of HAT processes is supported by theoretically calculated C—H bond dissociation energies (BDEs) and their qualitative correlation with HAT efficiencies.^[^ ^10^ ^]^ However, due to conformational restraints, this is not observed for substrates in the catalytic pocket of TET enzymes, as illustrated by empirical data from Xu et al.^[^ ^11^ ^]^ and calculations by Luo et al. (**Table** **1**).^[^ ^12^ ^]^

**Table 1:** C—H BDEs and observed catalytic efficiency parameters for the oxidation of **5mC**, **5hmC**, and **5fC** by TET2. The hydrate form **5dhmC** is shown as a possible transient intermediate.

Nucleobase	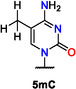	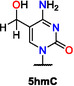	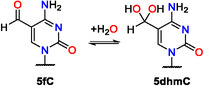	
C–H BDEa) [ kJ mol^−1^]	378.2	360.7	388.7	–
*k*_cat_/*K*_m_ b) [10^3 ^L mol^−1 ^s^−1^]	4.42	0.70	0.35	

a) CBS‐QB3, CPCM calculations as published by Xu et al.;^[^ ^21^ ^]^

b) *k*_cat_/*K*_m_ as published by Xu et al.^[^ ^21^ ^]^

The simplified TET biomimetic [Fe^IV^(O)(Py_5_Me_2_H)]^2+^ (**TM1**), first published by Chang et al.^[^ ^13^ ^]^ was shown to oxidize **5mC** as nucleobase,^[^ ^14^ ^]^ as nucleoside or as oligomeric nucleotide.^[^ ^15^ ^]^ Here, the calculated BDE values shown in Table 1 correspond well with the found reaction rates of **5mC** and **5hmC**. Accumulation of **5fC** during the oxidation was observed, but no validated reaction rate for the substrate has yet been reported (Table 1).^[^ ^14^ ^]^ As the **5dhmC** geminal diol was detected in (+)‐MS experiments^[^ ^9^ ^b]^ and has been implicated as an explanation of different base‐flipping kinetics,^[^ ^16^ ^]^ a transient hydrate formation during TET‐mediated **5fC** oxidation seems possible. **5dhmC** forms in a pH‐dependent manner and was found at 0.5% under acidic conditions.^[^ ^9^ ^]^ In contrast, the hydrate form of the noncanonical nucleotide 5‐formyl‐6‐aza‐cytidine (**1rb5f6aC**) was reported to be comparatively abundant at 20% by Carell et al.^[^ ^17^ ^]^ Kinetic measurements on selected substrates alongside the respective BDE value calculations might therefore aid in evaluating the importance of the hydrate adduct for **5fC** oxidation with potential implications regarding the relevance of direct deformylation and decarboxylation of methylcytosine derivatives.

## 2 Results and Discussion

### 2.1 Analytical Data

**Figure 2:**
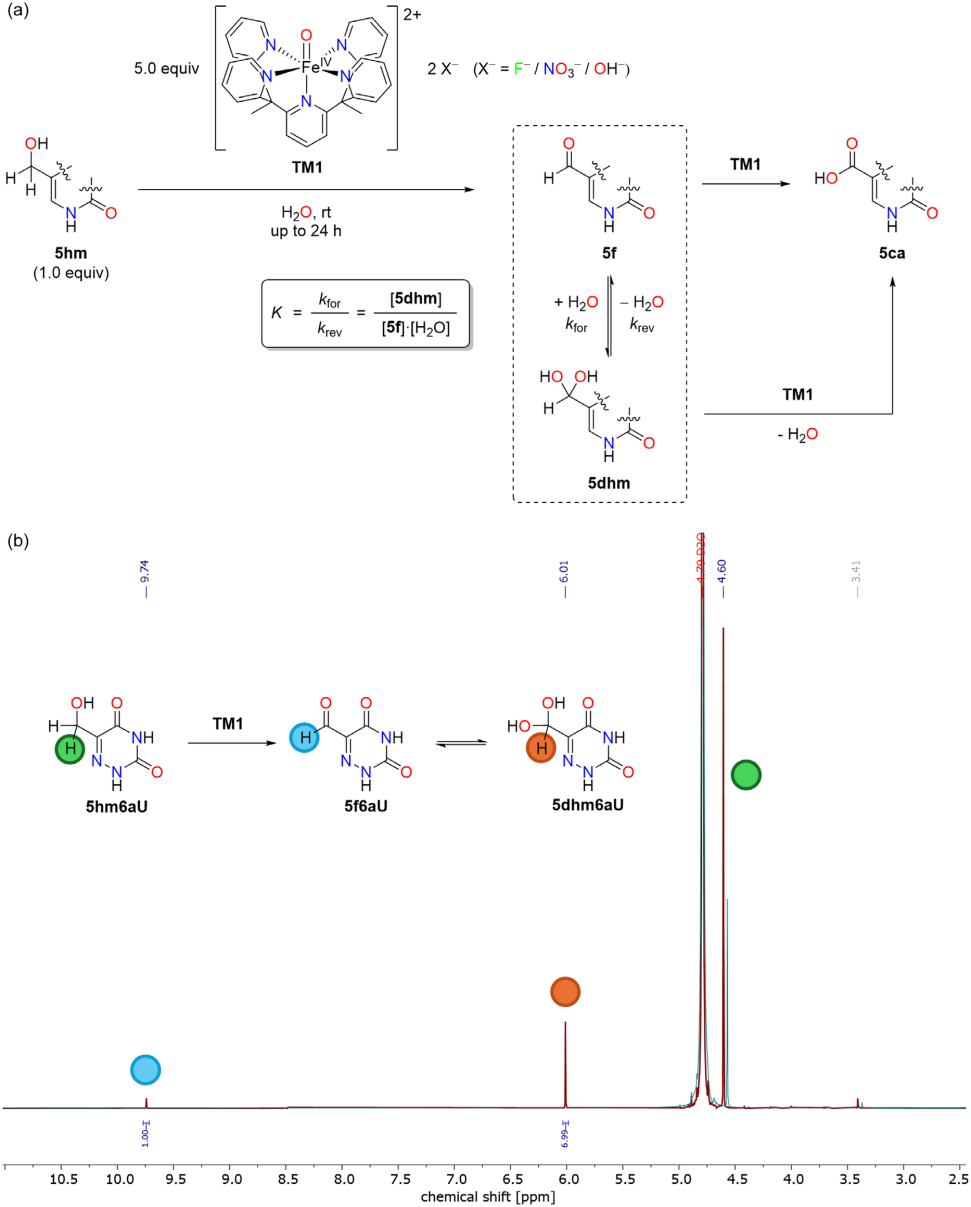
a) Reaction mechanism for the oxidation from 5‐hydroxymethyl (**5hm)** via 5‐formyl (**5f**) and 5‐dihydroxy (**5dhm**) to 5‐carboxyl (**5ca**) with biomimetic iron(IV)oxido complex **TM1** in water at room temperature. The equilibrium constant between **5f** and **5dhmU** is formulated as *K *= *k*_for_/*k*_rev_. b) ^1^H NMR spectrum in D_2_O of **5hm6aU** oxidation. Integration of the aldehyde and hydrate peaks revealed a distribution **5f6aU**:**5dhm6aU = **1:7 (88% hydrate). Reaction conditions: [**5hm6aU**] = [**TM1**] = 5 mM, H_2_O, 25 °C, 1 h.

**Figure 3:**
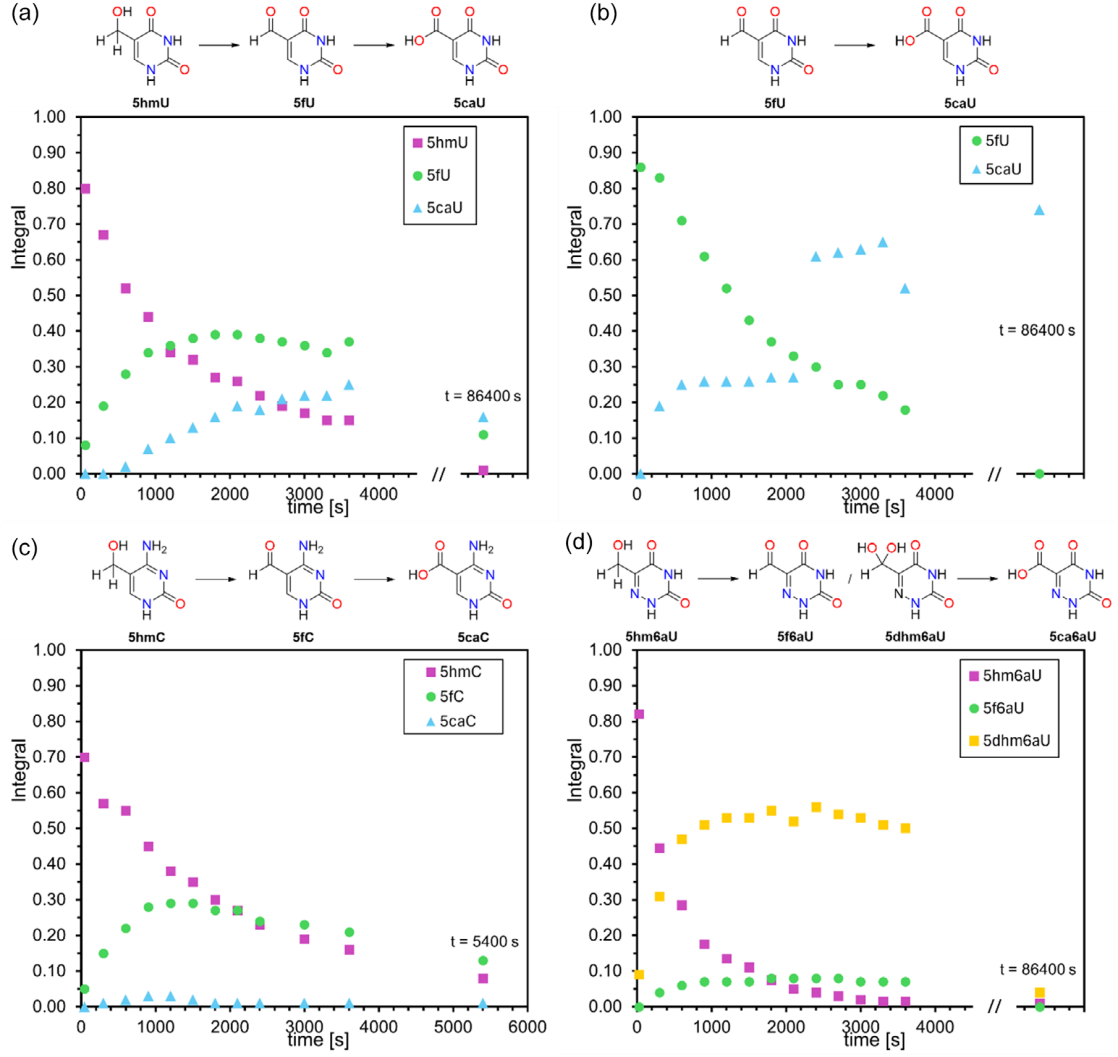
Raw ^1^H NMR integrals as observed for the oxidation cascade of a) **5hmU**, b) **5fU**, c) **5hmC**, and d) **5hm6aU**.

Following the procedure illustrated in **Figure** **2**, each oxidation reaction employed a substrate concentration of 0.2 mM and a fivefold excess (1.0 mM) of iron‐complex **TM1** at 25 °C. Synthesis of the iron complex followed literature procedures.^[^ ^13^ ^,^ ^18^ ^]^ After anion exchange to remove excess cerium nitrate species, **TM1** is obtained as an aqueous solution (10 mM) that was always used in situ and assumed to exist as a mixed anion system (including fluoride, nitrate, and hydroxide ions). Reaction kinetics were measured by taking samples at selected reaction times, followed by a work‐up step employing adsorptive filtration through a short silica gel column, and ^1^H NMR analysis of the filtrate using pyrazine as internal standard. The four species identified spectroscopically in oxidation experiments starting with **5hm** nucleobases include the starting material itself, the **5f** derivative in equilibrium with its hydrate form **5dhm**, and **5ca** as the endpoint of the oxidation cascade as shown in Figure 2a. The time‐dependent integrals observed for oxidation reactions starting from **5hmU**, **5fU**, **5hmC**, and 5‐hydroxymethyl‐6‐aza‐uracil (**5hm6aU**) have been collected in **Figure** **3**. The sum of reactant and product integrals decreases during the reaction, most evidently at longer reaction times. We attribute this to the loss of nucleobases on the silica gel column, minor side reactions, and partial degradation caused by the reactive iron(IV)‐oxido species. While no additional signals were identified in the ^1^H NMR spectra, it is likely that proton‐deficient or paramagnetic products are formed in trace amounts. Combination of the integrated signal intensities with the known internal standard concentrations provides time‐dependent concentrations of allreactants/intermediates/products and thus the basis for the subsequent kinetic analysis. Inspection of the results obtained for the oxidation of **5hmU** (Figure 3a) indicates **5fU** as the only detected intermediate and **5caU** as the final product. Effectively, the same result is obtained when starting the oxidation from **5fU** as shown in Figure 3b. While no hydrate could be detected as a transient intermediate in the oxidation of **5hmC** (Figure 3c), this species is the most abundant intermediate detected in the oxidation of **5hm6aU** (Figure 3d). Importantly, **5fC** and **5fU** both have extremely small hydration equilibria (*K *< < 10^−3^),^[^ ^19^ ^]^ which explains why their hydrate forms were not observed under the reaction conditions used in this study. By contrast, a hydrate content of 20% has been reported for 5‐formyl‐6‐aza‐cytidine (**1rb5f6aC**), which corresponds to *K *= 4.5 × 10^−3 ^L mol^−1^.^[^ ^17^ ^]^ Since uracil derivatives typically hydrate about 20‐fold more readily than their cytosine analogs, we extrapolated *K*(**5f6aU**) ≈ 9.0 × 10^−2 ^L mol^−1^, which predicts an equilibrium hydrate content of 83% (see Figure S1 in Supporting Information). Strikingly, our NMR experiments on **5f6aU** oxidation show a constant diol content of ≈88%, in excellent agreement with the estimated 83% hydrate content (Figure 3d). The fact that this ratio remains essentially unchanged over the entire reaction time, where **5f6aU** is being continually oxidized by **TM1** to the final **5ca6aU** product, demonstrates that the aldehyde‐hydrate equilibrium is established much faster than the oxidation itself. This justifies treating the hydration step as a rapid pre‐equilibrium in our kinetic simulations (see below) and highlights the mechanistic role of the geminal diol in directing the subsequent iron(IV)‐oxido mediated oxidation of **5f6aU**.

### 2.2 Kinetic Modeling

Numerical microkinetics simulations with the COPASI package where employed to fit the time dependent concentrations of all species to the general oxidation mechanism shown in Figure 2a. The reaction steps considered in the kinetic models also include the slow first‐order self‐deactivation of the **TM1** oxidant to its reduced form (**TM2**) quantified in independent experiments ([**TM1**]_0 _= 1.0 mM, *k*_TM _= 1.75·10^−5 ^s^−1^). Using the oxidation cascade of **5hmU** via **5fU** to **5caU** as an example, thus yields the following kinetic model

step 0:

(1) TM1 → TM2

(2) $$-\frac{d\left[TM1\right]}{dt}=k_{\mathrm{TM}}\left[TM1\right]$$

step 1:

(3) 5hmU + TM1 → 5fU + TM2

(4) $$-\frac{d\left[5hmU\right]}{dt}=\frac{d\left[5fU\right]}{dt}=k(5hmU)\left[TM1\right]\left[5hmU\right]$$

step 2:

(5) 5fU + TM1 → 5caU + TM2

(6) $$-\frac{d\left[5fU\right]}{dt}=k(5fU)\left[TM1\right]\left[5fU\right]$$

Preliminary experiments indicated that ommitting the last measurement at very long reaction times (*t *= 86,400 s) and treating the initial substrate concentration as a variable (rather than a fixed quantity) gave the best results for all four substrates. This is not surprising as some susbtrates reacted faster than others and within the used setup, starting concentrations at the first measurement point are thus slightly different. Initial fitting of the rate constant for **5hmU** oxidation to the decay of this species, and subsequent use of the optimized value in fitting the rate constant for **5fU** oxidation to the concentrations of all species provided the most robust approach for evaluation of the overal kinetic scheme and gave *k*(**5hmU**) = 0.66 ± 0.02 L mol^−1 ^s^−1^ and *k*(**5fU**) = 0.43 ± 0.01 L mol^−1 ^s^−1^.

### 2.3 Data Analysis

The rate constants derived from the primary data shown in Figure 3 for the oxidation of **5hmU**, **5hmC**, and **5hm6aC** are collected in **Table** **2**. The measurement type “direct” indicates that the rate constant was derived as the first step of the overal oxdiation cascade, while “indirect” indicates a rate constant for oxidation of a transient intermediate.

**Table 2:** Rate constants (*k*) and their standard deviations (*δk*) for the side‐chain oxidation of different nucleobases.

Base	Cascade	Step No.	Measurement	*k*	*δk*
				[L mol^−1 ^s^−1^]	[L mol^−1 ^s^−1^]
**5hmU**	**5hm → 5f → 5ca**	1	direct	0.66	0.02
**5fU**		2	indirect	0.43	0.01
**5fU**	**5f → 5ca**	1	direct	0.48	0.01
**5hmC**	**5hm → 5f → 5ca**	1	direct	0.51	0.01
**5fC**		2	indirect	0.53	0.03
**5hm6aU**	**5hm → 5f/5dhm → 5ca**	1	direct	1.87	0.06
**5f6aU**		2	indirect	2.86	0.26
**5dhm6aU**		2	indirect	0.24	0.01

**Figure 4:**
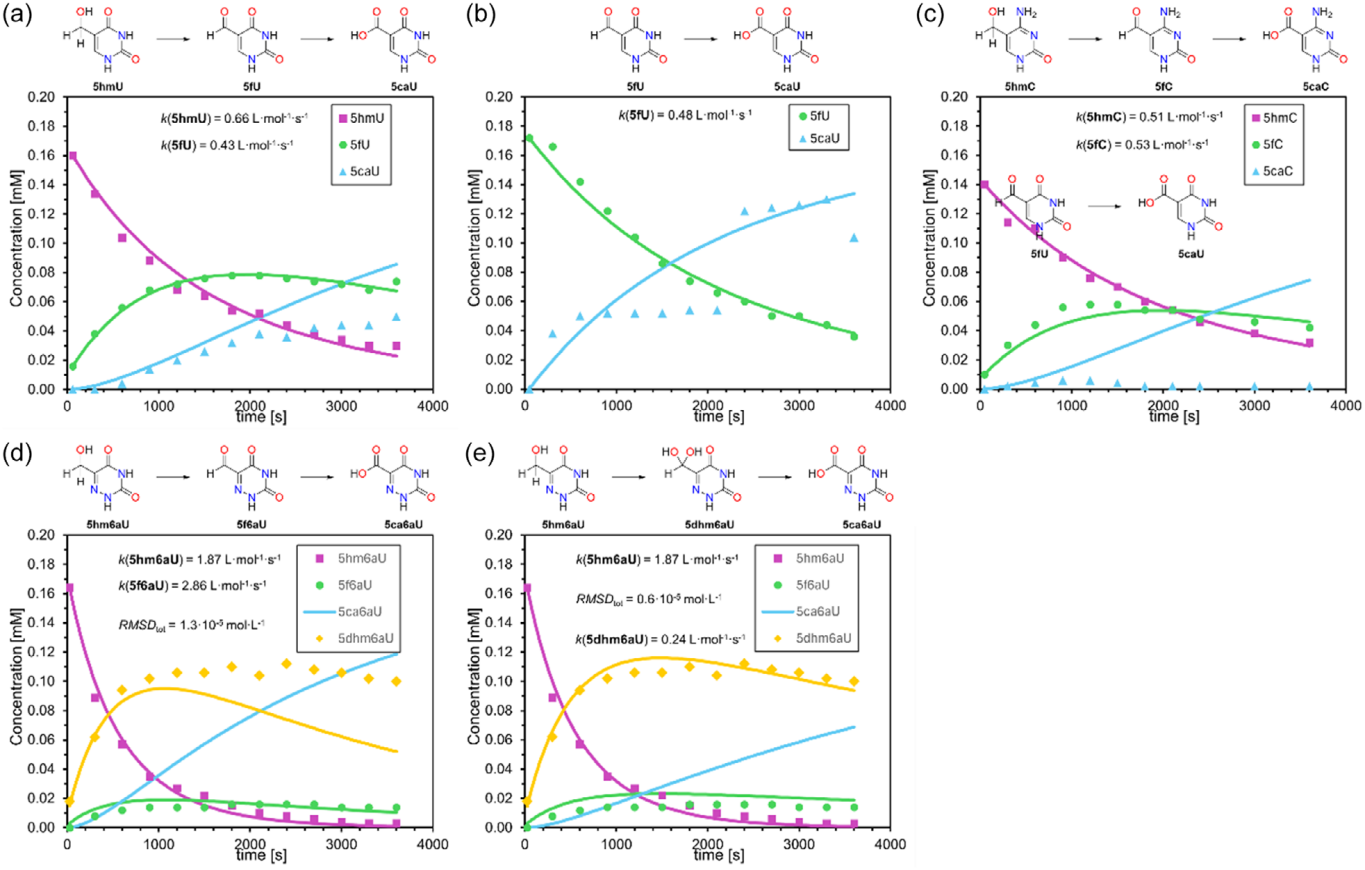
Overlay of simulated data with original data for the oxidation cascade of a) **5hmU**, b) **5fU**, c) **5hmC**, d) **5hm6aU** via **5f6aU**, and e) **5hm6aU** via **5dhm6aU** with the determined rate constants and the overall RMSD_tot_ value for all observed species.

In how far the direct and indirect measurement types yield different values was tested for the oxidation of **5fU**, whose direct measurement gave *k*(**5fU**) = 0.43 ± 0.01 L mol^−1 ^s^−1^. Determination of the same rate constant as the second step of the oxdiation cascade starting from **5hmU** gave a closely similar value of *k*(**5fU**) = 0.48 ± 0.01 L mol^−1 ^s^−1^, which demonstates the robustness of our kinetic analysis. The fidelity of the rate constant determinations can also be inferred from the standard deviations collected in Table 2, and also from visual inspection of **Figure** **4**, where experimentally measured turnover data is combined with the simulated reaction progress as defined by the rate constants. The predicted decay of **5hmU**, as well as the intermediate rise and subsequent decrease of **5fU** as a transient intermediate, are quite well reproduced by the simulated turnover cuves. In contrast, the accurate quantification of **5caU** suffers from the chosen work‐up method, as is reflected in the kinetic fits and raw data shown in Figure 4a,b. This implies that accurate kinetic data will only be obtained by following the concentrations of **5hmU** and **5fU**, but not that of **5caU**. The same observation can be made for **5hmC** as the initial substrate, where rate constants for the oxidation of **5hmC** and **5fC** are close to those obtained for the uracil derivatives before, and where quantification of the final **5caC** product is again not possible in a quantitative manner. Kinetic analysis of **5hm6aU** oxidation differs from the first two substrates in that the first step of the oxidation cascade is much faster than for the non‐aza parent systems with *k*(**5fU**) = 1.87 ± 0.06 L mol^−1 ^s^−1^, and where **5f6aU** and **5dhm6aU** are both detected as true intermediates of the oxidation cascade.

**Figure 5:**
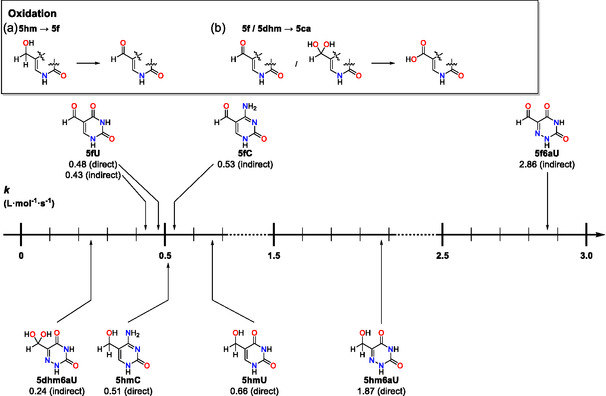
Second‐order rate constants (L mol^−1 ^s^−1^) for the single‐step oxidation of a) hydroxymethyl‐ and b) formyl‐substituted nucleobases by iron complex **TM1**.

The experimentally determined second order rate constants *k* for the oxidation step converting **5hm**‐ to **5f**‐, and **5f**‐ to **5ca**‐nucleobases are summarized in a graphical manner in **Figure** **5**. Oxidation of **5hmC** and **5hmU** proceeds at largely similar reaction rates of 0.51 and 0.66 L mol^−1 ^s^−1^, respectively. Oxidation of **5hm6aU** to its **5f**‐derivative is significantly faster with a rate constant of 1.87 L mol^−1^ s^−1^, indicating that substitution at the C6 position with nitrogen (6‐aza‐substitution) accelerates the transformation significantly.

Kinetic analysis of the oxidation of **5f** nucleobases is complicated by the possible involvement of aldehyde hydrates in the oxidation cascade, even when direct detection of these hydrates by ^1^H NMR measurements is not possible or inconclusive. This is the case for **5fU**, where the known equilibrium constant for hydrate formation amounts to *K*(**5fU**) = 2.94·10^−4 ^L mol^−1^.^[^ ^19^ ^]^ Kinetic analysis of the oxidation of **5fU** thus assumes no involvement of the respective hydrate form and yields rate constants of 0.48 L mol^−1 ^s^−1^ (direct method) and 0.43 L mol^−1 ^s^−1^ (indirect method), both of which are identical within experimental error and only marginally lower than that for **5hmU** at 0.66 L mol^−1 ^s^−1^ (Table 2). For **5fC**, the hydration equilibrium is even less favorable than for **5fU**. Equilibrium constants have been proposed based on the limit of detection (*K *= 4.05 ·10^−5^) and the limit of quantification (*K *= 1.22 × 10^−5^) in ^1^H NMR measurements, yielding an average value of *K *= 2.64 × 10^−5 ^L mol^−1^.^[^ ^19^ ^]^ Assuming that oxidation of **5fC** proceeds without the involvement of its hydrate form yields *k*(**5fC**) = 0.53 L mol^−1 ^s^−1^, which is only marginally faster than oxidation of **5fU**.

The oxidation of **5f6aU** is mechanistically more complex in that two plausible reaction pathways exist for its conversion to **5ca6aU**. The first involves oxidation of **5f6aU** without the involvement of any **5dhm6aU** oxidation. This yields a rate constant of *k*(**5f6aU**) = 2.86 L mol^−1 ^s^−1^ together with a comparatively large cumulative root mean square deviation (RMSD_tot_) value (Figure 4). The second pathway assumes that only **5dhm6aU** is oxidized out of the hydration equilibrium with **5f6aU**, which yields *k*(**5dhm6aU**) = 0.24 L mol^−1 ^s^−1^ together with a more favorable RMSD_tot_ value (Figure 4). This rate constant is counterintuitively small, but results from the comparatively high concentration of **5dhm6aU** in the hydration equilibrium. This further strengthens our assumption that the oxidation over the hydrate route better describes the oxidation cascade. Kinetics simulations with both oxidation pathways active simultaneously met with significant numerical problems but confirm that the aldehyde‐hydrate equilibrium is established on a timescale much faster than oxidation, and that most of the substrate is oxidized via the hydrate form. Consequently, it is both chemically and kinetically more appropriate to treat hydration as a rapid pre‐equilibrium and to describe the system by a single, effective oxidation rate of the hydrated species. This insight underpins our final kinetic model and reinforces the mechanistic conclusion that geminal‐diol formation is an obligate and dominant feature of **5f6aU** oxidation by the iron(IV)‐oxido biomimetic complex **TM1**. The results also underscore how small changes in nucleobase structure can markedly alter the oxidation kinetics. Electron‐withdrawing substituents modulate the susceptibility of the **5hm** group to undergo oxidation, and **5hm6aU** is the most rapidly oxidized to its **5f** form among the nucleobases tested, whereas **5hmC** and **5hmU** proceed at more moderate but still comparable rates.

### 2.4 Mechanistic Insights from Comparison to BDE(C—H) Values

How C—H bond abstraction from formyl hydrates relates to that from the parent aldehydes can be assessed by inspection of the respective BDE(C—H) values. Using the same theoretical approach as employed in a recent reactivity analysis of 5‐methyl substituted nucleobases,^[^ ^10^ ^]^ we have now computed the BDE(C—H) values for the **5hm**, **5f**, and **5dhm** derivatives of the pyrimidine bases studied here (**Table** **3**). For each of the bases studied here, we find the BDE(C—H) values to be largest for the formyl C—H bonds, followed by those for the hydroxymethyl C—H bonds, and lowest for the C—H bonds in the aldehyde hydrates. Closer inspection of Table 3 shows that the BDE(C—H) values for the three formyl group C—H bonds in **5fC**, **5f6aU**, and **5fU** are closely similar (within 6 kJ mol^−1^), while this is not so for the hydrates of these nucleobases. For these latter systems, the weakest C—H bond is found for **5dhm6aU** at BDE(C—H) = 313.8 kJ mol^−1^, which is more than 40 kJ mol^−1^ lower than that for **5dhmU** at BDE(C—H) = 354.1 kJ mol^−1^. Despite this apparent lack of systematic bond energy trends, we can nevertheless employ the computed BDE(C—H) values for correlation analysis with the experimentally determined oxidation rate constants. These are also collected in Table 3 together with their logarithmic forms. For **5fU** oxidation, an average rate constant *k*(**5fU**) = 0.46 L mol^−1 ^s^−1^ was used. For the other species, there is only one value each.

**Table 3:** Comparison of calculated BDEs and observed reaction rates.

Base	BDE(C—H)	*k*	ln *k*
	[kJ mol^−1^]	[L mol^−1 ^s^−1^]	
Previous study^[10]^	–	–	–
**1mU**	416.0	0.06	−2.81
**1mC**	414.8	0.06	−2.81
**5mC**	387.3	0.18	−1.71
**1,5dmC**	386.5	0.30	−1.20
**1,5dmU**	385.7	0.44	−0.82
**5mU**	383.1	0.63	−0.46
**5miC**	379.4	1.44	+0.36
This study	–	–	–
**5fC**	401.0	0.53	−0.64
**5f6aU**	395.4	2.86	+1.05
**5fU**	394.7	0.46	−0.78
**5hmC**	367.4	0.51	−0.67
**5hmU**	360.0	0.66	−0.42
**5dhmU**	354.1	(27.66)	(+3.32)
**5hm6aU**	337.7	1.87	+0.63
**5dhmC**	322.1	(364.23)	(+5.90)
**5dhm6aU**	313.8	0.24	−1.43

As already mentioned above, the equilibrium constants for the hydration of **5fC** and **5fU** are extremely low, but we can nevertheless explore the scenario where the oxidation of these systems proceeds solely through the respective hydrate forms. For **5fC** the average value for the equilibrium constant *K*(**5fC**) = 2.64·10^−5 ^L mol^−1^ was used, while for **5fU** we employ the known equilibrium constant *K*(**5fU**) = 2.94·10^−4 ^L mol^−1^.^[^ ^19^ ^]^ For **5f6aU** the equilibrium constant has not been determined under stationary conditions, and we therefore employ here the extrapolated value of *K*(**5f6aU**) = 9.0 × 10^−2 ^L mol^−1^ mentioned already above. In dilute solutions, it is practical to consider that the equilibrium between the formyl and hydrate is rather quickly established, which can be reflected in the kinetics simulations by assuming a fast forward rate constant of *k*_for _= 1000 L mol^−1 ^s^−1^ in combination with a reverse rate constant *k*_rev_ such that the ratio of both yields the equilibrium constant. The overall kinetics scheme then is

step 0:

(7) TM1 → TM2

(8) $$-\frac{d\left[\mathrm{TM}1\right]}{dt}=k_{\mathrm{TM}}\left[\mathrm{TM}1\right]$$

step 1:

(9) 5hm + TM1 → 5f + TM2

(10) $$-\frac{d\left[5\mathrm{hm}\right]}{dt}=k(5\mathrm{hm})\left[\mathrm{TM}1\right]\left[5\mathrm{hm}\right]$$

step 2:

(11) $$5f\;+\;H_{2}O\;↔\;5\mathrm{dhm}$$

(12) $$-\frac{d\left[5f\right]}{dt}=k_{\mathrm{for}}\left[5f\right]\left[H_{2}O\right]$$

(13) $$-\frac{d\left[5\mathrm{dhm}\right]}{dt}=k_{\mathrm{rev}}\left[5d\mathrm{hm}\right]$$

step 3:

(14) $$5\mathrm{dhm}\;+\;\mathrm{TM}1\;\to \;5\mathrm{caC}\;+\;\mathrm{TM}2\;+\;H_{2}O$$

(15) $$-\frac{d\left[5\mathrm{dhm}\right]}{dt}=k(5\mathrm{dhm})\left[\mathrm{TM}1\right]\left[5\mathrm{dhm}\right]$$

Applying this kinetic model yields rate constants for **5dhmU** of *k *= 26.2 ± 0.4 L mol^−1 ^s^−1^ for the indirect method and *k *= 29.2 ± 0.7 L mol^−1 ^s^−1^ for the direct method, the average of which is *k*(**5dhmU**) = 27.7 L mol^−1 ^s^−1^, while for **5dhmC** we obtain a notably large value of *k*(**5dhmC**) = 364.2 ± 20.7 L mol^−1 ^s^−1^ (Table 3). The rather large rate constants for **5dhmU** and **5dhmC** simply result from the rather low concentration of the respective hydrates under equilibrating conditions. Assuming that reaction proceeds through the respective hydrates does not lead to an improved fit of the turnover curves to the kinetic model for these two species. This is different for oxidation of **5f6aU** where the oxidation route via the hydrate form **5dhm6aU** provides the best fit for the turnover curves of **5f6aU** and **5dhm6aU**.

**Figure 6:**
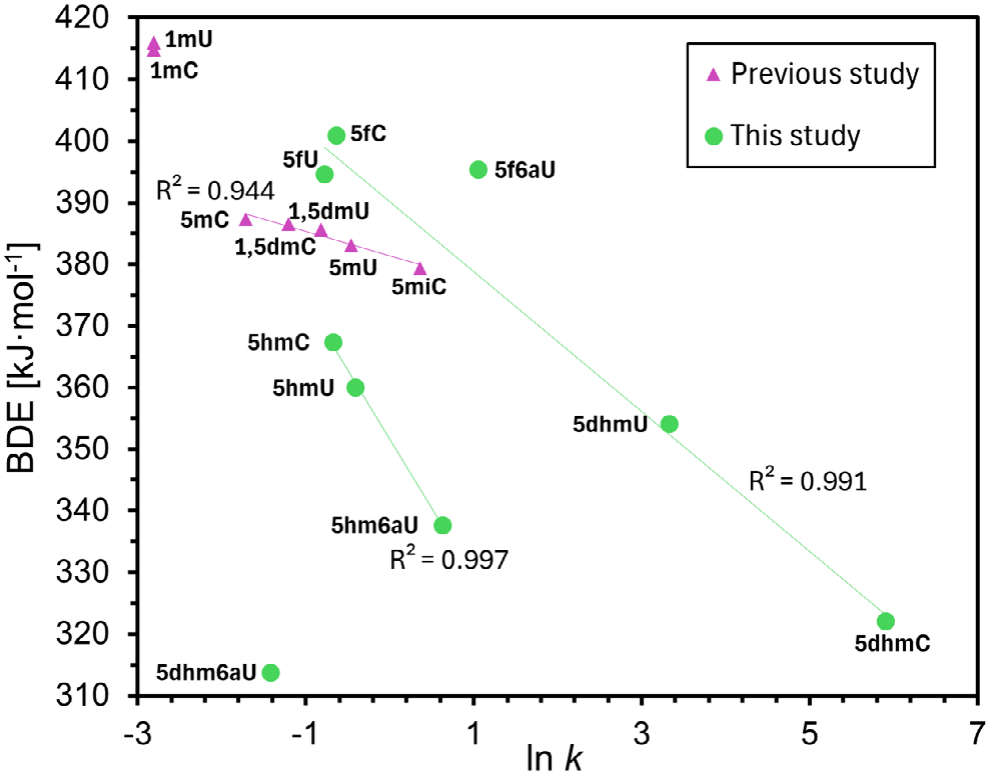
Correlation of the experimentally derived rate constants for the oxidation cascades with the calculated bond dissociation energies (BDEs) determined in this study in comparison with our previous study.^[^ ^10^ ^]^

With the newly derived rate constants for the hydrate forms in hand, we can explore a possible correlation with the respective BDE(C—H) values (**Figure** **6**). For the correlation of the calculated BDE values with the natural logarithm of the experimentally measured second‐order rate constants *k*, we observe a linear relationship for the three **5hm** species (*R* ^2 ^= 0.997). As expected, the correlation predicts an increase in oxidation rate with decreasing BDE(C—H) values. Extension of this correlation to significantly higher BDE(C—H) values appears to cross through data points for the oxidation of methylated cytosine and uracil derivatives determined in earlier studies,^[^ ^10^ ^]^ but not those for the three formyl derivatives investigated in the current study. If we were to include **5fC** and **5fU** in the data set, the *R* ^2^ value drops to 0.677, suggesting a less satisfactory correlation. Despite the structural diversity, a second inverse correlation between BDE and ln *k* (*R* ^2 ^= 0.991) appears to include **5fC** and **5fU** with their hydrated counterparts **5dhmC** and **5dhmU**, but not the respective 6‐aza variants **5f6aU** and **5dhm6aU**. This may again suggest that the oxidation of the aza‐substituted **5f6aU** proceeds through a different mechanism. We may thus conclude that oxidation proceeds through the hydrate for the species that exhibit high hydrate‐formation, combined with the fact that the BDE for the C—H of the geminal diol is much lower than the original formyl group, thereby facilitating the oxidation.

## 3 Conclusion

We have combined quantitative NMR experiments, numerical kinetic modeling, and density functional theory calculations to clarify the mechanism of side‐chain oxidation in 5‐substituted nucleobases by a biomimetic iron(IV)‐oxido complex (**TM1**). Time‐resolved ^1^H NMR measurements allowed monitoring the stepwise conversion of 5‐hydroxymethyl (**5hm**) to 5‐formyl (**5f**) and ultimately to 5‐carboxyl (**5ca**) derivatives for a series of substrates (**5hmU**, **5fU**, **5hmC**, and **5hm6aU**). By accounting for the oxidant's slow self‐deactivation and properly selecting initial and final turnover points, we obtained robust second‐order rate constants *k* spanning two orders of magnitude from **5hmC** to **5hm6aU**, 0.51–1.87 L mol^−1 ^s^−1^, for the direct **5hm** to **5f** step. Notably, the 6‐aza substitution in **5hm6aU** accelerates hydrogen‐atom abstraction by almost threefold, underscoring the influence of heterocycle electronics on reactivity. For the oxidation from **5f** to **5ca**, we distinguished two scenarios: the direct oxidation of **5f** or the oxidation of the hydrate counterparts (indirect pathway). We found that the numerical description of direct oxidation for the nucleobases with low hydrate formation tendency, **5fC** and **5fU**, is sufficient. However, for the nucleobase with significant hydration, **5f6aU**, the oxidation via **5dhm6aU** better fits the whole oxidation cascade. Complementary DLPNO‐CCSD(T)/CBS calculations of C—H BDEs reveal an excellent inverse correlation (*R* ^2 ^≈ 0.99) between bond strength and oxidation rate across the **5hm** series, supporting that hydrogen‐atom abstraction is rate‐determining. For formyl substrates (**5fU** and **5fC**) with sufficiently low hydration equilibria, a linear correlation with their hydrates (**5dhmU** and **5dhmC**) may be understood such that minimal hydration doesn’t change the reaction mechanism. However, the significantly increased level of hydration of **5f6aU** suggests that the rate‐determining step from C—H abstraction at the aldehyde switches to that at its hydrate counterpart **5dhm6aU**. Together, this comprehensive study shows that direct H‐atom abstraction governs **5hm** oxidation, while hydrate‐mediated pathways can dominate **5f** oxidation when the aldehyde hydration is thermodynamically favorable. This mechanistic duality not only has important implications for TET‐catalyzed demethylation in epigenetics but also provides design principles for tailoring biomimetic oxidants toward selective C—H activation in nucleic acids.

## Conflict of Interest

The authors declare no conflict of interest.

## Supporting information

Supplementary Material

## Data Availability

The data that support the findings of this study are available in the Supporting Information of this article. The data is also accessible on the Zenodo archive with DOI:10.5281/zenodo.17142840.
