# Supplementary material for: Aldehyde or Hydrate? Investigation into the Oxidation of 5‐Formylcytosine Derivatives Using a Computational and Experimental Approach
Source: Chembiochem. 2025 Sep 23;26(21):e202500480. doi: 10.1002/cbic.202500480 (PMC12596925; doi:10.1002/cbic.202500480)
Supplement: Supplementary file 1 — Supplementary Material [file CBIC-26-e202500480-s001.pdf]

Supporting Information I

Aldehyde or Hydrate? Investigation into the Oxidation of 5-Formylcytosine Derivatives using a Computational and Experimental Approach

Kuangjie Liu[§], Annika Menke[§], Fabian L. Zott[§], Domenic Mayer, Lena J. Daumann*, Hendrik Zipse*

lena.daumann@hhu.de

Faculty of Mathematics and Natural Sciences, Heinrich-Heine-Universität Düsseldorf, Universitätsstr. 1, 40225 Düsseldorf, Germany

zipse@cup.uni-muenchen.de

Faculty of Chemistry and Pharmacy, Ludwig-Maximilians University Munich, Butenandtstr. 5-13, 81377 Munich, Germany

Table of Contents

S1. General Information and Techniques 3

S2. Synthetic Procedures 5

S2.1 Iron(IV) Complex $[Fe^{IV}L1(O)]^{2+}$ (TM1) 5

S2.2 Nucleobase Substrates 6

S3. Development of Kinetics Study 10

S3.1 Design of Oxidation Experiments 10

S3.2 Analytical Procedures 11

S3.2.1 Sampling and Work-Up 11

S3.2.2 ^{1}H NMR Data Collection 11

S3.2.3 Normalized vs. Non-Normalized Data 12

S3.3 Raw Data 12
S4. Data Analysis 18
S4.1 Analysis of 5hmU Oxidation 19
S4.1.1 Selection of Data Points 19
S4.2 Analysis of 5fU Oxidation 25
S4.2.1 Indirect Determination via 5hmU Oxidation Cascade 25
S4.2.2 Direct Determination via 5fU Oxidation 27
S4.3 Analysis of 5hmC Oxidation 28
S4.4 Analysis of 5fC Oxidation 29
S4.5 Analysis of 5hm6aU Oxidation 31
S4.6 Analysis of 5f6aU Oxidation 32
S4.6.1 Oxidation only from 5f6aU 35
S4.6.2 Oxidation only from 5dhm6aU 37
S4.7 Simulation of Oxidation Cascade 41
S4.7.1 5hmU Oxidation 41
S4.7.2 5hmC Oxidation 42
S4.7.3 5hm6aU Oxidation 43
S5. Results and Discussion 44
S5.1 Overview of Determined Rate Constants 44
S5.2 Correlation Between BDE and Oxidation Rate Constants 46
S6. Appendix 53
S7. Literature 59

S1. General Information and Techniques

General Methods

Reactions sensitive to air and moisture were done under N_2. Glassware and stirring bars were stored in the oven (125 °C) prior to use. Syringes were purchased from VWR. Mechanical pipettes (2-20 µL, 20-200 µL, 100-1000 µL, and 0.25-2.5 mL, Eppendorf Research plus, single channel) were used with pipette tips (0.1-20 µL, 2-200 µL, 50-1000 µL, and 0.25-2.5 mL, Eppendorf Biopur, epT.I.P.S.) for kinetic sample preparation. Reagents and solvents were purchased from ABCR, Merck, TCI, Thermo Fischer, and VWR and were used as received unless specified otherwise. A Christ Alpha 1-2 LDplus Lyophilisator (LYO) was used to remove water. Molecular sieve was activated by acetone washing, oven drying (125 °C), and vacuum drying in oil bath (150 °C) overnight prior to use. Silica gel for chromatography (60, irregular 40-63 µm) was purchased from VWR. TLC plates (60 F254, unmodified, aluminum backed, 2 µm) were purchased from Merck. Sample vials (ND8, 1.5 mL, 11.6×32 mm, clear) with caps (ND8, screw caps with septa, white silicone/red PTFE) were purchased from VWR. Pressure tubes (Ace, bushing type, Back seal, 15 mL, 10.2×2.54 cm) were purchased from Merck.

NMR Spectroscopy

All ^{1}H NMR spectra were recorded at room temperature either on Jeol ECP 270 (400 MHz), Jeol ECX 400 or Bruker Avance III, Varian INOVA 400 or 600 machines in D_2O, DMSO-*d6* or 0.18 M phosphate buffer (pH = 7.4) spiked with 20% D_2O at 400 or 600 MHz. All ^{13}C NMR spectra were recorded respectively at 101 or 151 MHz. Chemical shifts (δ) are reported in parts per million (ppm), referenced to DMSO-*d6* (δ = 2.50 ppm for ^{1}H; δ = 39.52 ppm for ^{13}C), or D_2O (δ = 4.79 ppm for ^{1}H; ^{13}C signals are automatically referenced to the deuterium lock). Spectra were processed with MestreNova 11.0.4 software. Automatic phase correction and default baseline correction were used prior to analysis. Coupling constants are reported in Hertz (Hz), and splitting patterns are designated as singlet (s), doublet (d), triplet (t), quartet (q), multiplet (m), broad singlet (br).

Mass Spectrometry

Mass spectra were recorded using an Advion expressionL compact mass spectrometer (CMS) with the atmospheric solid analysis probe (ASAP) technique. The ion source was set to "low temperature and low fragmentation" and spectra were acquired with a mass range of 500 m/z at a speed of 104 m/z units per second. Analysis was performed using Advion's CheMS Express 5.1.0.2 software after background subtraction. High-resolution mass spectrometry (HRMS) spectra were obtained on a Thermo Finnigan LTQ FT machine with either electron impact ionization (EI, 70 eV) or electrospray ionization (ESI). Samples were prepared at concentration 0.1-1 mg/mL in methanol. For ESI measurements, samples were spiked with 0.1-1% formic acid to aid ionization.

Elemental Analysis

Elemental analysis (EA) of C, H, N, and S was performed using the vario EL and vario micro cube systems from Elementar, with high-temperature digestion, dynamic gas component separation, and detection in a single apparatus. Samples were weighed in tin boats. Combustion of the samples occurred at 1150 °C in an oxygen-enriched helium atmosphere. The resulting combustion products were collected and separated with gas chromatography. The quantification of C, H, N, and S was achieved by detecting CO_2, H_2O, N_2, and SO_2, with a thermal conductivity detector (TCD). The system with three adsorption columns allowed for the sequential removal of H_2O, SO_2, and CO_2 from the gas mixture, with nitrogen as the only unaffected component. Each element's concentration was calculated through the recorded output signal in TCD over time.

COPASI Analysis

We employed COPASI software package 4.44 (Build 295) to perform numerical study of the oxidation reactions. Kinetic constants were determined using "Parameter Estimation" function with "Evolutionary Programming". For the simulation of the oxidation cascade with determined rate constants were simulated using the "Time Course" function with "Deterministic (LSODA)" and an interval size of 0.01 s. Unless otherwise specified, the other settings remained default.

S2. Synthetic Procedures

S2.1 Iron(IV) Complex $[Fe^{IV}L1(O)]^{2+}$ (TM1)

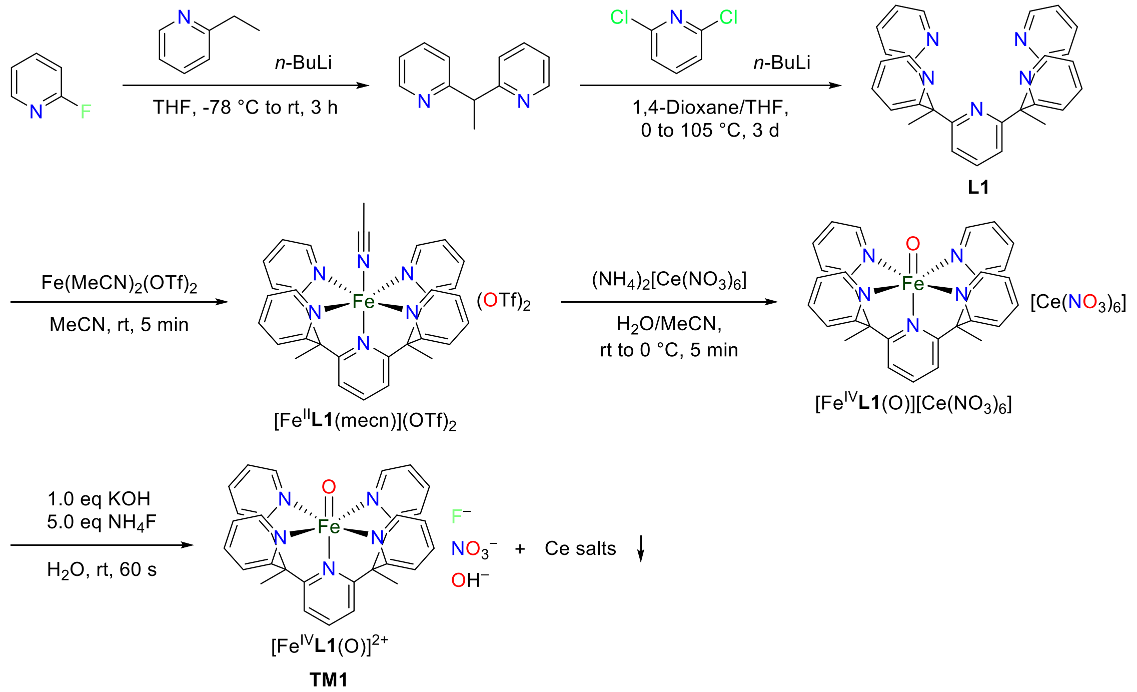

Scheme S1. Synthesis of **TM1**.

Synthesis of the iron complex followed literature procedures.[1] After anion exchange, the iron complex (**TM1**) is obtained as an aqueous solution (10 mM) that was always used *in situ*. The anion is assumed to consist of a mixture of fluoride, nitrate and hydroxide ions. For clarity, the anions were left out in the following figures where **TM1** is present.

S2.2 Nucleobase Substrates

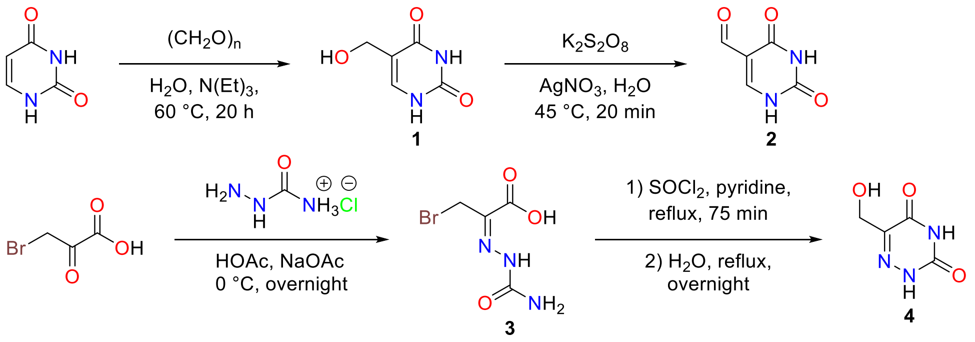

Scheme S2. Syntheses of **5hmU** (**1**), **5fU** (**2**), **5hm6aU** (**4**).

5-Hydroxymethyluracil (1)

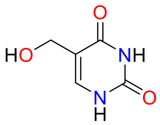

Modified literature procedure[2]:

Triethylamine (42 mL, 300 mmol,1.5 eq) was added to a suspension of uracil (22.4 g, 200 mmol) and paraformaldehyde (18.0 g, 600 mmol) in water (600 mL). The solution was stirred at 60 °C overnight. Water was removed yielding a yellowish viscous oil, to which ethanol (100 mL) was added and stored at -20 °C for several hours. The formed white precipitate was filtered and washed with cold ethanol (3 × 20 mL). The filter cake was recrystallized from 95% aqueous ethanol yielding 5-hydroxymethyluracil as a white solid (19.5 g, 137.5 mmol, 69%, **1**).

^{1}H NMR (400 MHz, DMSO-*d6*): δ = 11.06 (s, 1H, N-H), 10.71 (s, 1H, N-H), 7.24 (s, 1H, 6-H), 4.85 (t, J = 4 Hz, 1H, O-H), 4.10 (t, J = 4 Hz, 2H, 7-H) ppm.

^{13}C NMR (101 MHz, DMSO-*d6*): δ = 163.8, 151.4, 138.2, 112.7, 55.8 ppm.

EA: Calcd for $C_5H_6N_2O_3$ [%] C 42.26, N 19.71, H 4.26; found [%] C 41.99, N 19.66, H 4.24.

HRMS (ESI, in MeOH): m/z calcd for $C_5H_5N_2O_3$ [M - H$^+$] 141.0306; found 141.0305.

R$_f$ (25% MeOH/5% NH_4OH/DCM, UV) = 0.30

5-Formyluracil (2)

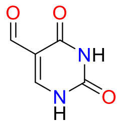

Modified literature procedure[2]:

5-Hydroxymethyluracil (24.6 mmol, 3.5 g, 1.0 eq.) was dissolved in water (130 mL) by heating to 90 °C. The solution was cooled to 45 °C and potassium persulfate (45.1 mmol, 12.2 g, 1.8 eq.) and silver nitrate (0.74 mmol, 3 mol%, 12.53 mmol) were added. The mixture was stirred at 45 °C for 20 min. After cooling to 0 °C, the precipitated product was collected by filtration and washed with cold water (3 × 5 mL). The filtrate was concentrated, and the precipitate was collected in this manner two more times yielding 5-formyluracil (17.5 mmol, 2.45 g, 72%, **2**) as a white solid.

^{1}H NMR (400 MHz, DMSO-*d6*): δ = 11.91 (s, 1H, N-H), 11.51 (s, 1H, N-H), 9.73 (s, 1H, C(O)-H), 8.14 (s, 1H, 6-H) ppm.

^{13}C NMR (100 MHz, DMSO-*d6*): δ = 186.49, 162.53, 150.53, 149.41, 110.13 ppm.

EA: Calcd for $C_5H_4N_2O_3$ [%] C 42.87, N 20.00, H 2.88; found [%] C 40.32, N 19,87, H 2.84.

HRMS (ESI, in MeOH): *m/z* calcd for $C_5H_3N_2O_3$ [M - H$^+$] 139.0149; found 139.0148.

R$_f$ (25% MeOH/5% NH_4OH/DCM, UV) = 0.45

Semicarbazone (3)

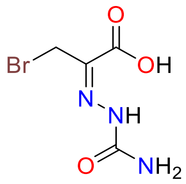

Modified literature procedure[3]:

A solution of 3-bromopyruvic acid (5.0 g, 30.0 mmol, 1.0 eq.) in 15 mL acetic acid and 5 mL HPLC grade H_2O was firstly prepared in a 100 mL flask at 0 °C in a water-ice mixture. A solution of semicarbazide hydrochloride (3.34 g, 30.0 mmol, 1.0 eq.) and anhydrous NaOAc (3.20 g, 38.6 mmol, 1.3 eq.) in HPLC grade H_2O (25 mL) was prepared and then added dropwise with a pipette to the reaction mixture. After 10 min, the reaction solution turned milky. The mixture was then allowed to naturally warm up to room temperature over 2.5 h, followed by cooling back to 0 °C in a cryostat filled by isopropanol for 15 h overnight. The resulting white precipitate was filtered, washed with cold HPLC grade H_2O (3 × 5 mL) and then dried Et_2O (3 × 5 mL). Semicarbazone was yielded as a mixture of (E)- and (Z)-isomers (3.9 g, 17.5 mmol, 58 %, **3**) as a white solid and used for next step without further purification.

^{1}H NMR (400 MHz, DMSO-*d6*): δ = 10.58 (s, 0.4H, NN-H), 10.51 (s, 0.6H, NN-H), 6.98 (br, 2H, CON-H), 4.57 (s, 0.8H, 3-H), 4.46 (s, 1.2H, 3-H) ppm.

EA: Calcd for $C_4H_6N_3O_3Br$ [%] C 21.45, N 18.76, H 2.70; found [%] C 23.68, N 20.34, H 2.90.

HRMS (EI, in MeOH): *m*/*z* calcd for $C_4H_6N_3O_3Br$ [$M^{\cdot+}$] 222.9587; found 222.9585.

5-Hydroxymethyl-6-aza-uracil (4)

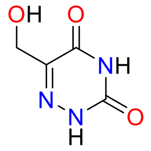

Modified literature procedure[3]:

$SOCl_2$ (60 mL) and anhydrous pyridine (0.2 mL) were added to semicarbazone (2.8 g, 12.4 mmol, 1.0 eq) under N_2 atmosphere. The system was refluxed at 90 °C for 75 min and then cooled to room temperature. The volume was concentrated in vacuo to 15 mL and cooled to -20 °C overnight. The formed yellow precipitate was filtered, and the filtrate was extracted with DCM (3 × 20 mL). DCM was removed, combined with the previously collected filter cake, dissolved in HPLC grade H_2O (30 mL), refluxed at 120 °C overnight, and then lyophilized yielding 5-hydroxymethyl-6-azauracil (1.41 g, 9.87 mmol, 79 %, **4**) as a bright yellow solid.

^{1}H NMR (400 MHz, DMSO-*d6*): δ = 12.17 (s, 1H, N-H), 11.94 (s, 1H, N-H), 4.87 (br, 1H, O-H), 4.25 (s, 2H, 7-H) ppm.

^{13}C NMR (101 MHz, DMSO-*d6*): δ = 156.8, 149.6, 144.4, 58.0 ppm.

EA: Calcd for $C_4H_5N_3O_3$ [%] C 33.57, N 29.36, H 3.52; found [%] C 31.97, N 28.09, H 3.52.

HRMS (ESI, in MeOH): *m/z* calcd for $C_4H_4N_3O_3$ [M - H^+] 142.0258; found 142.0258.

R_f (25% MeOH/5% NH_4OH/DCM, UV) = 0.15

S3. Development of Kinetics Study

S3.1 Design of Oxidation Experiments

In order to investigate the oxidation of 5hm-substituted nucleobases by an iron-complex (TET-mimetic), we set up a series of reactions in aqueous solution at 25 °C. Each reaction employed a substrate concentration of 0.4 mM (0.15 mmol nucleobase in 375 mL MQ-water) and a five-fold 2.0 mM of the freshly prepared $[Fe^{IV}(O)(\mathbf{L1})]^{2+}2X^{-}$ ($X = F^{-}$, $NO3^{-}$, or OH^{-}) solution. The clock started at the moment of mixing. Samples (50 mL) were withdrawn after 25 s, every 5 min between 5 to 60 min and after 24 h, to ensure that both rapid initial changes and long-term reactions could be tracked.

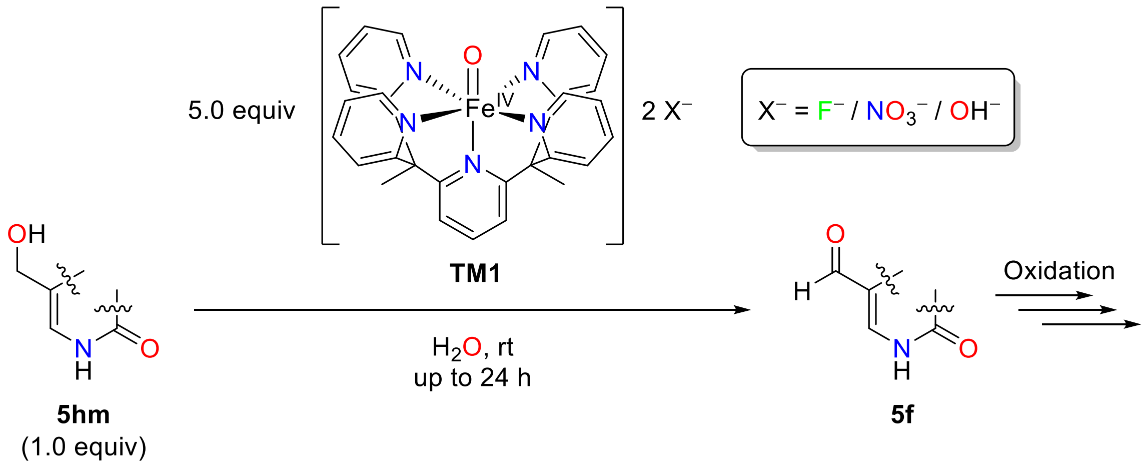

Figure S1. Oxidation of 5-hydroxymethyl (**5hm**) to 5-formyl (**5f**) side chain as an example of reaction design for the oxidation of nucleobases with our biomimetic iron-oxo complex (**TM1**) in water at room temperature.

S3.2 Analytical Procedures

S3.2.1 Sampling and Work-Up

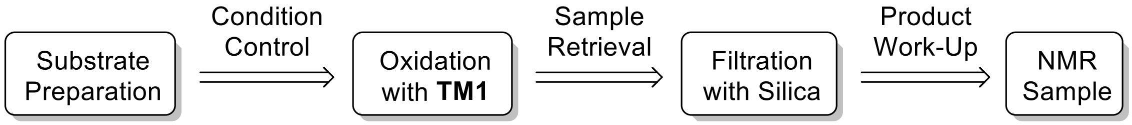

Scheme S3. Workflow of the kinetic study, starting from preparation the substrate solution, mixing of substrate solutions with **TM1** solution in a controlled way, retrieving of reaction samples in certain time steps, to reaction work-up for NMR measurements.

Each aliquot was rapidly filtered through a short column of silica (diameter 2.2 cm; length 8.5 cm; filled with silica gel 6.5 ± 0.1 g) in vacuo to remove any precipitates or colloidal iron species. A 100 mL round-bottom flask was connected to the column at the bottom to collect the oxidation products. The column was then washed with MQ-water (20 mL) to reduce the loss of our compounds on the column, and vacuum was drawn for 2 min each. The collected samples were lyophilized, redissolved in MQ-water (5 mL), transferred into 15 mL falcon tubes, and then lyophilized again. In the end of our working flow, residues were redissolved in D_2O (0.5 mL) containing pyrazine (0.2 mM) as an internal standard and transferred to NMR tubes for immediate ^{1}HNMR measurements.

S3.2.2 ^{1}H NMR Data Collection

Integrals for the substrate-related signals were referenced to the integrated pyrazine signal (set to four protons). Where applicable, the relevant H-peak for each species were:

for **U** and **C**, the proton at the 6-position of the pyrimidine ring (CH): 5hmU at 7.64 ppm, 5fU at 8.43 ppm, 5caU at 8.38 ppm, 5hmC at 7.75 ppm, 5fC at 8.48 ppm, 5caC at 8.35 ppm;

for **6a5hmU**, the methylene protons (CH_2): at 4.60 ppm;

for **6a5fU**, the formyl proton (CHO) at 9.74 ppm and the diol protons ($C(OH)_2$) at 6.01 ppm;

for **6a5caU**, no suitable proton was present in ^{1}H NMR for quantification.

This internal standard approach allows direct comparison of integral values to the known pyrazine concentration, yielding absolute concentrations of the various oxidation products.

Slight variations of the chemical shift were occasionally observed especially for the carboxy species due to a change in pH during the reaction between **TM1** and the substrates, which was also observed in our previous work.[1b] Reference spectra of the starting materials as well as examples of the reaction mixtures are shown in section S6.

S3.2.3 Normalized vs. Non-Normalized Data

Two methods of reporting integrals could be taken into consideration:

1. **Non-normalized:**
   The raw integrals (referenced only to the pyrazine standard) directly reflect the observed ^{1}H NMR intensity.
2. **Normalized:**
   The integrals for each sample are scaled such that the sum of all detectable substrate- and product-related protons equals 1.

We decided to go for the non-normalized approach, the reason was that the loss of 5caU was unavoidable, leading to overscaling of the normalized concentrations. In the tables below, we primarily present non-normalized integrals, however, a final step of normalization can be applied if needed for consistent comparisons across different experiments

S3.3 Raw Data

The following tables summarize the time-resolved NMR integrals for key species in each oxidation experiment. The "Sum" column indicates the total of all pyrimidine-ring or formyl/methylene signals at each time point. In some experiments, a decrease in total integral is observed. This decline is attributed to minor side reactions or partial degradation of products, catalyzed by the reactive iron(IV)-oxo species. Furthermore, especially the carboxy species might not be completely washed through the silica column but be retained to some extent, which also leads to a lower amount of species observed.

Table S1. Oxidation cascade of **5hmU**.

**Sample** No.	**Time** s	**5hmU** Integral	**5fU** Integral	**5dhmU** Integral	**5caU** Integral	**Sum** Integral
**1**	60	0.80	0.08	-	0	0.88
**2**	300	0.67	0.19	-	0	0.86
**3**	600	0.52	0.28	-	0.02	0.82
**4**	900	0.44	0.34	-	0.07	0.85
**5**	1200	0.34	0.36	-	0.10	0.80
**6**	1500	0.32	0.38	-	0.13	0.83
**7**	1800	0.27	0.39	-	0.16	0.82
**8**	2100	0.26	0.39	-	0.19	0.84
**9**	2400	0.22	0.38	-	0.18	0.78
**10**	2700	0.19	0.37	-	0.21	0.77
**11**	3000	0.17	0.36	-	0.22	0.75
**12**	3300	0.15	0.34	-	0.22	0.71
**13**	3600	0.15	0.37	-	0.25	0.77
**14**	86400	0.01	0.11	-	0.16	0.28

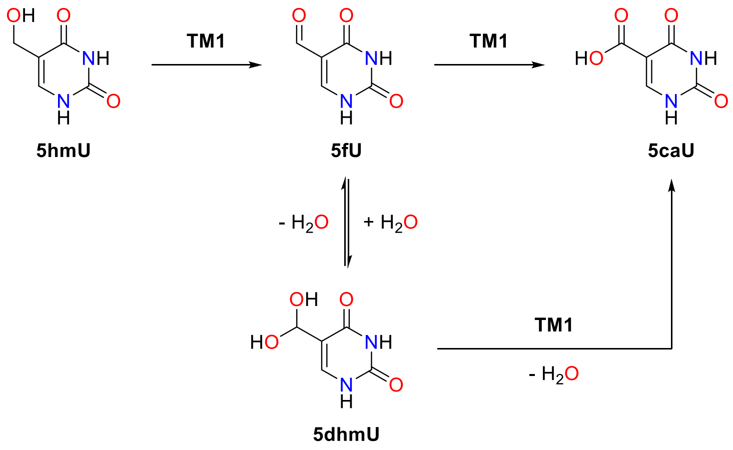

Scheme S4. Oxidation cascade from **5hmU** via **5fU** and **5dhmU** to **5caU**.

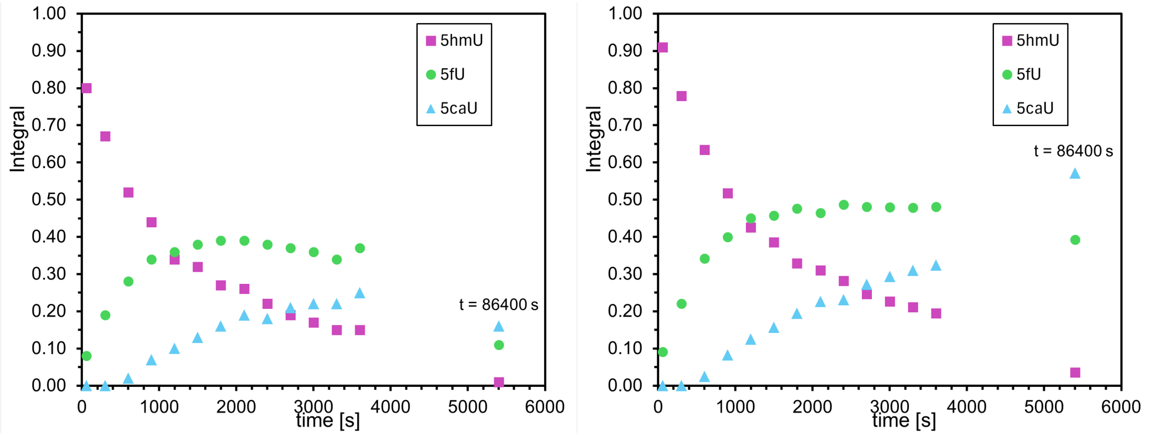

Figure S2. Integrals as observed (left) and normalized integral from sum (right) for the oxidation cascade of **5hmU** (**Table S1**).

Table S2. Direct oxidation of **5fU**.

**Sample No.**	**Time** s	**5hmU** Integral	**5fU** Integral	**5dhmU** Integral	**5caU** Integral	**Sum** Integral
**1**	45	-	0.86	-	0	0.86
**2**	300	-	0.83	-	0.19	1.02
**3**	600	-	0.71	-	0.25	0.96
**4**	900	-	0.61	-	0.26	0.87
**5**	1200	-	0.52	-	0.26	0.78
**6**	1500	-	0.43	-	0.26	0.69
**7**	1800	-	0.37	-	0.27	0.64
**8**	2100	-	0.33	-	0.27	0.60
**9**	2400	-	0.3	-	0.61	0.91
**10**	2700	-	0.25	-	0.62	0.87
**11**	3000	-	0.25	-	0.63	0.88
**12**	3300	-	0.22	-	0.65	0.87
**13**	3600	-	0.18	-	0.52	0.70
**14**	86400	-	0	-	0.74	0.74

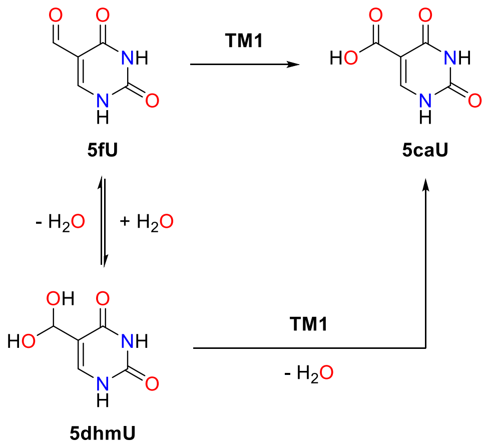

Scheme S5. Oxidation cascade from **5fU** and **5dhmU** to **5caU**.

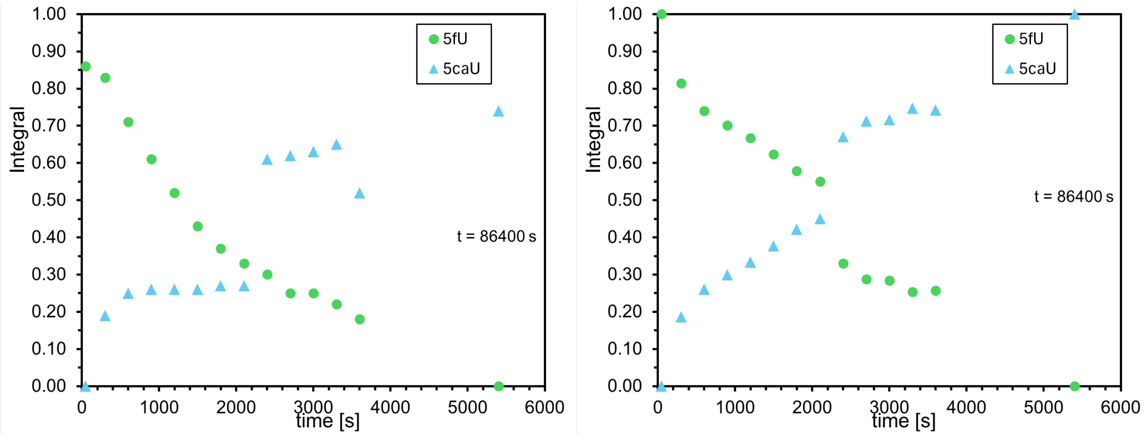

Figure S3. Integrals as observed (left) and normalized integral from sum (right) for the oxidation cascade of **5fU** (**Table S2**).

Table S3. Oxidation cascade of **5hmC**.

Sample No.	Time s	5hmC Integral	5fC Integral	5dhmC Integral	5caC Integral	Sum Integral
**1**	45	0.70	0.05	-	0	0.75
**2**	300	0.57	0.15	-	0.01	0.73
**3**	600	0.55	0.22	-	0.02	0.79
**4**	900	0.45	0.28	-	0.03	0.76
**5**	1200	0.38	0.29	-	0.03	0.70
**6**	1500	0.35	0.29	-	0.02	0.66
**7**	1800	0.30	0.27	-	0.01	0.58
**8**	2100	0.27	0.27	-	0.01	0.55
**9**	2400	0.23	0.24	-	0.01	0.48
**10**	3000	0.19	0.23	-	0.01	0.43
**11**	3600	0.16	0.21	-	0.01	0.38
**12**	5400	0.08	0.13	-	0.01	0.22

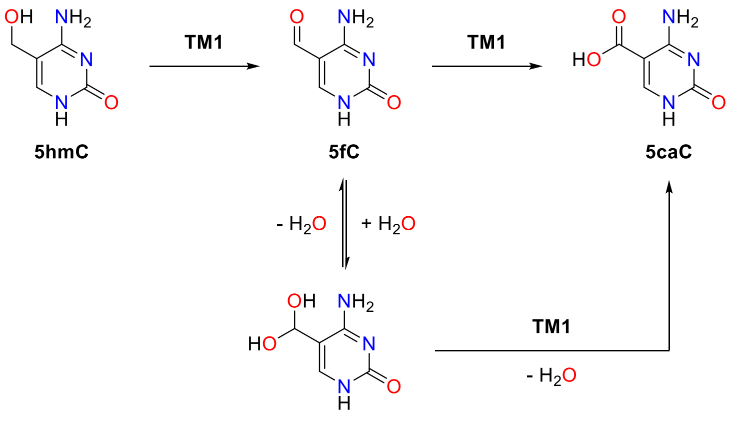

Scheme S6. Oxidation cascade from **5hmC** via **5fC** and **5dhmC** to **5caC**.

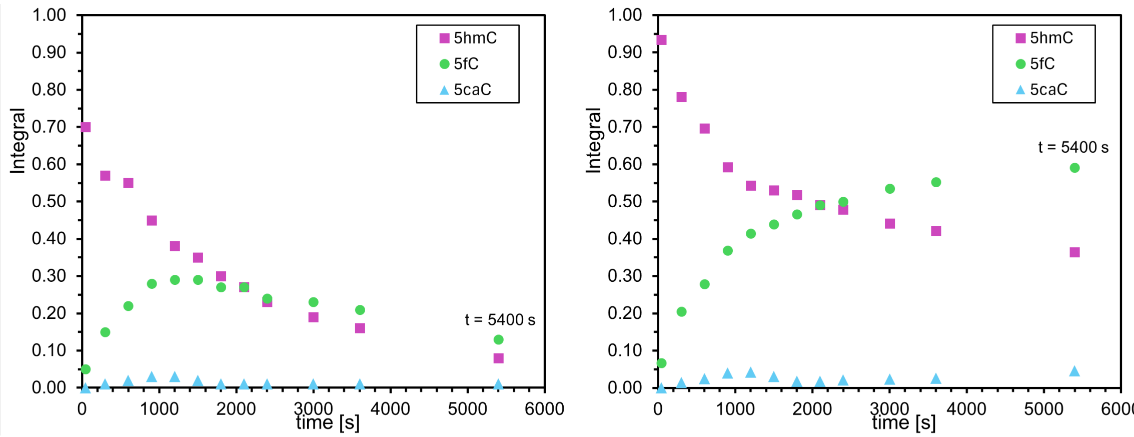

Figure S4. Integrals as observed (left) and normalized integral from sum (right) for the oxidation cascade of **5hmC** (**Table S3**).

Table S4. Oxidation cascade of **5hm6aU**.

Sample No.	Time s	5hm6aU Integral	5f6aU Integral	5dhm6aU Integral	5f : 5dhm	5ca6aU Integral	Sum Integral
**1**	25	0.82	0.00	0.09	0.00	-	0.91
**2**	300	0.445	0.04	0.31	0.13	-	0.80
**3**	600	0.285	0.06	0.47	0.13	-	0.82
**4**	900	0.175	0.07	0.51	0.14	-	0.76
**5**	1200	0.135	0.07	0.53	0.13	-	0.74
**6**	1500	0.11	0.07	0.53	0.13	-	0.71
**7**	1800	0.075	0.08	0.55	0.15	-	0.71
**8**	2100	0.05	0.08	0.52	0.15	-	0.65
**9**	2400	0.04	0.08	0.56	0.14	-	0.68
**10**	2700	0.03	0.08	0.54	0.15	-	0.65
**11**	3000	0.02	0.07	0.53	0.13	-	0.62
**12**	3300	0.015	0.07	0.51	0.14	-	0.60
**13**	3600	0.015	0.07	0.50	0.14	-	0.59
**14**	86400	0.01	0.00	0.04	0.00	-	0.05
					(Avg: 0.13)		

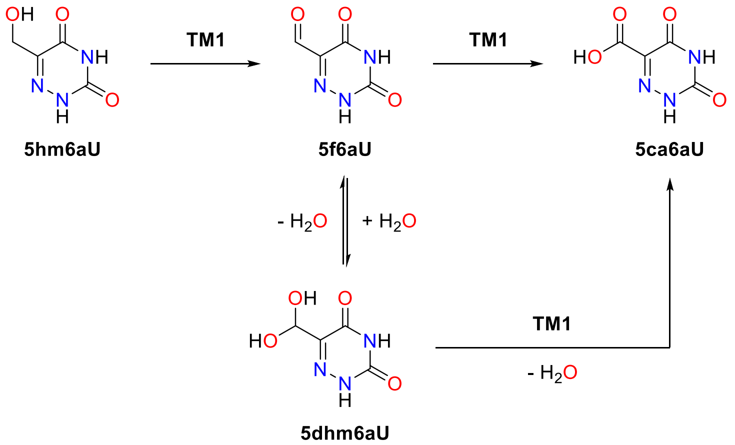

Scheme S7. Oxidation cascade from **5hm6aU** via **5f6aU** to **5ca6aU**.

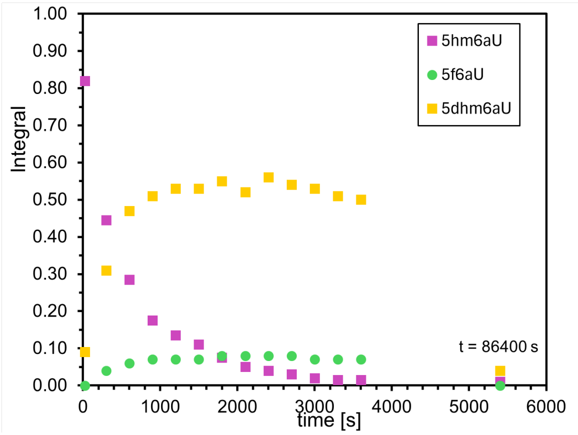

Figure S5. Integrals as observed for the oxidation cascade of **5hm6aU** (**Table S4**).

The sum of integrals sometimes decreases during the reaction, most evidently at longer reaction times. We attribute this to the loss of nucleobases on the column, minor side reactions, and partial degradation caused by the reactive iron(IV)-oxido species. While no additional signals were identified in the ^{1}H NMR spectra, it is likely that proton-deficient or paramagnetic products are formed in trace amounts. The NMR spectra of the reaction between **TM1** and **5hm6aU** reveal significant side products from the first measured point on. The substrate **5hm6aU** is consumed very fast and not only turns into the expected oxidation products but seems to form decomposition products also in a fast reaction. This side reaction presumably consumes significant amounts of **TM1** leading to higher amounts of accumulated oxidation product **5f6aU** than expected instead of fast further oxidation of **5f6aU** towards **5ca6aU**. The reference sample of **5fC** used in preliminary measurements were contaminated, which prevented reliable kinetic analysis, so these data are omitted from the tables. For the synthesis of **5f6aU**, despite multiple attempts, oxidation starting from **5hm6aU** could not be halted at the formyl **5f6aU** stage, and only **5ca6aU** was ever obtained. Therefore, the direct oxidation measurement from **5f6aU** was omitted as well. By combining the integrated signal intensities from **5hmU**, **5fU**, **5hmC**, and **5hm6aU** with the known internal standard concentration, these data constitute the basis for our subsequent kinetic modeling (**section S4**).

S4. Data Analysis

As we have previously discussed, we assumed a reaction model starting from **5hm** species, which could be further oxidized to **5f** species. Depending on the electronic nature of the nucleobases for hydration, the **5f** species stand in different equilibrium with the corresponding hydrates, their 5dhm counterparts (**Scheme S8**). Both could then be further oxidized into their final **5ca** products.

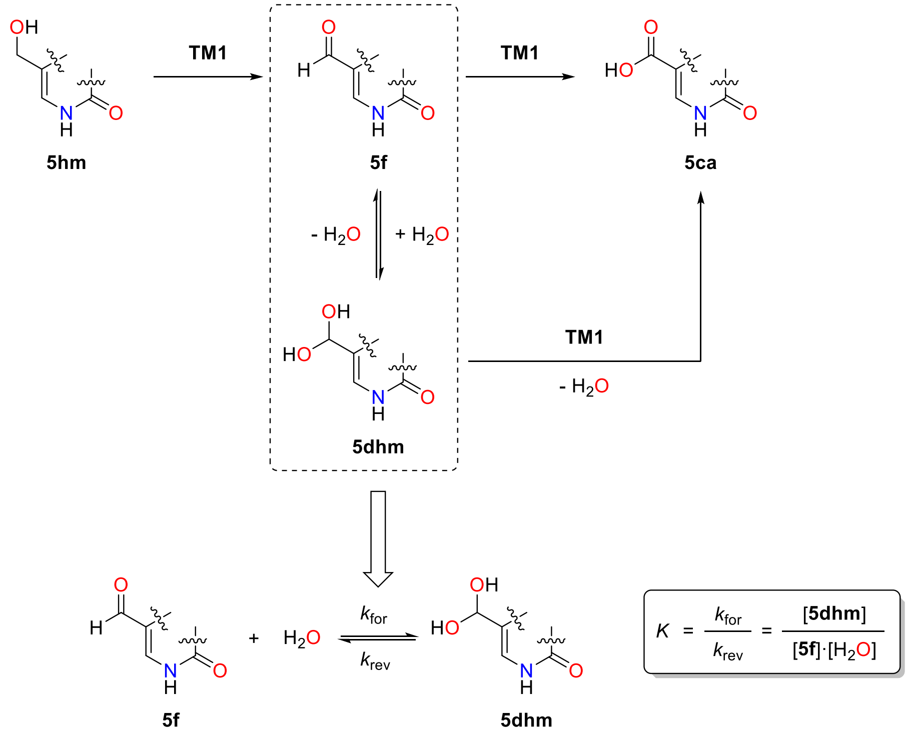

Scheme S8. Reaction model for oxidation from **5hm** via **5f** and **5dhm** to **5ca**, with **5f** and **5dhmU** in an equilibrium with a constant of *K*.

S4.1 Analysis of 5hmU Oxidation

Since **5hmU** is the initial substrate in the oxidation cascade leading to **5fU** and then **5caU**, we focused on fitting its concentration profile under various kinetic assumptions. Experiments were carried out such that the TET-biomimetic iron-oxo complex (**TM1**) was present at 5 eq (1.0 mM) relative to **5hmU** (0.2 mM). The time-resolved concentrations ([**5hmU**], [**5fU**], [**5caU**]) were determined via quantitative NMR. We consider the following reaction at the beginning:

$$\mathbf{5hmU} + \mathbf{TM1} \rightarrow \mathbf{5fU} + \mathbf{TM2} \tag{4.1}$$

S4.1.1 Selection of Data Points

For the initial concentration, we could either assume the starting concentration of **5hmU** to be precise by design (t_0 = 0 s, [**5hmU**]$_0$ = 0.2 mM), or we could use the earliest measured **5hmU** concentration as the initial value in the calculation (t_0 = 60 s, [**5hmU**]$_0$ = 0.16 mM), which eliminates potential errors during the preparation of the stock solution. For a bimolecular reaction, we started our calculation via second-order kinetics (eq. (4.2)) and compared multiple fitting strategies.

$$-\frac{d[\mathbf{5hmU}]}{d\mathrm{t}} = k(\mathbf{5hmU})[\mathbf{TM1}]_0[\mathbf{5hmU}] \tag{4.2}$$

S4.1.1.1 All Points with No Adjustments

Using all data points and keeping the initial **5hmU** concentration at 0.2 mM gives the results shown in **Figure S6**. It is apparent that the fit looks quite poor due to the last data point yielding a rate constant of k(**5hmU**) = 0.838359 ± 0.045892 L·mol^{-1}·s^{-1}.

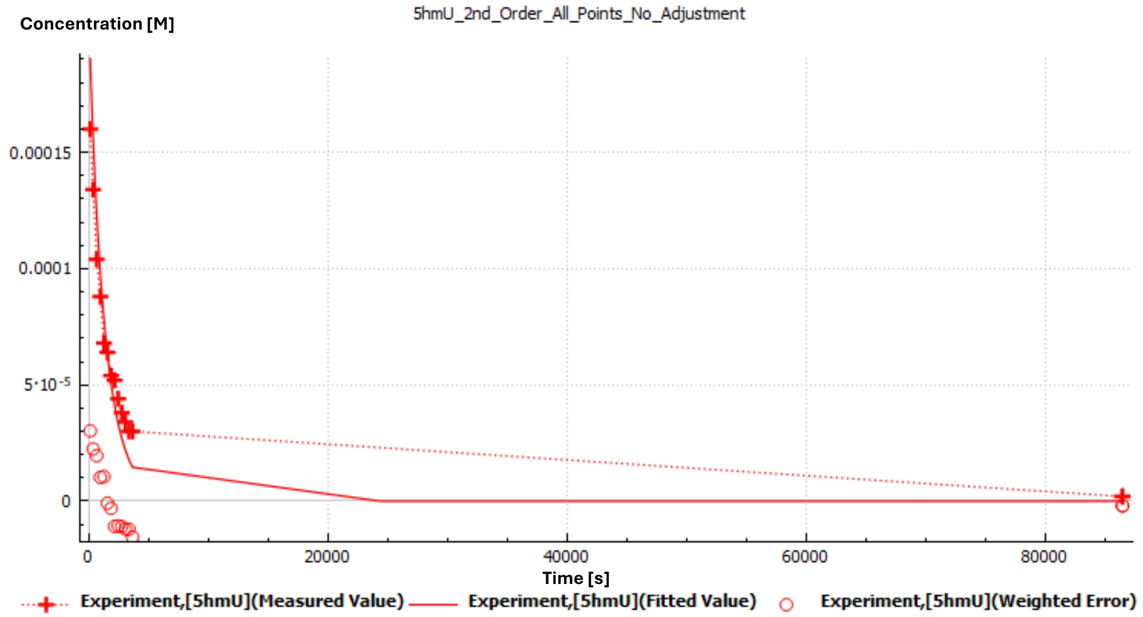

Figure S6. Measured and fitted data for the decay of **5hmU**. All data were selected without further adjustments. Substrate concentrations are given in M and reactions times in s. Residuals are shown as open circles.

S4.1.1.2 Exclusion of the Final Point

Excluding the last experimentally determined value (t = 86400 s, [**5hmU**] = 0.002 mM) from the data set and refitting the data yields the results shown in **Figure S6**.

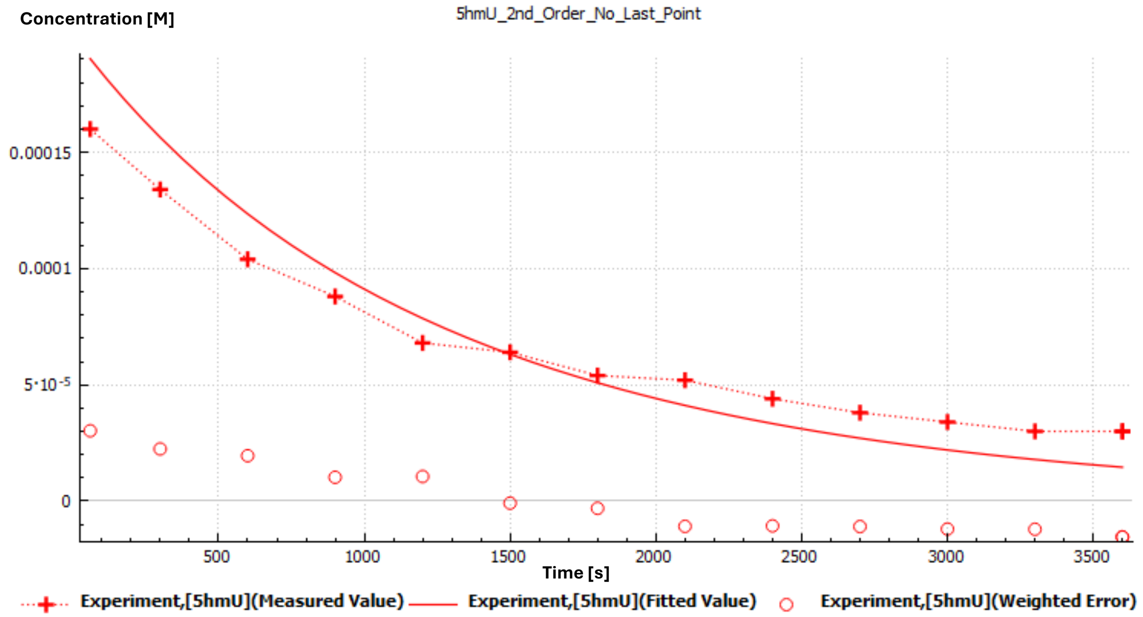

Figure S7. Measured and fitted data for the decay of **5hmU**. All data except the last data point were selected. Substrate concentrations are given in M and reactions times in s. Residuals are shown as open circles.

The quality of the fitted curve looks better yielding a rate constant of k(**5hmU**) = 0.838360 ± 0.047733 L·mol^{-1}·s^{-1}, but there remains a deviation between the curve and the measured data points and the rate constant didn't improve as expected.

S4.1.1.3 Exclusion of the Designed Initial Point

Replacing the initial assumed substrate concentration (t_0 = 0 s, [**5hmU**]$_0$ = 0.2 mM) by the first experimentally measured value (t_0 = 60 s, [**5hmU**]$_0$ = 0.16 mM) yields further improvements in fitting the substrate oxidation reaction and a slightly reduced rate constant of k(**5hmU**) = 0.644261 ± 0.018292 L·mol^{-1}·s^{-1}.

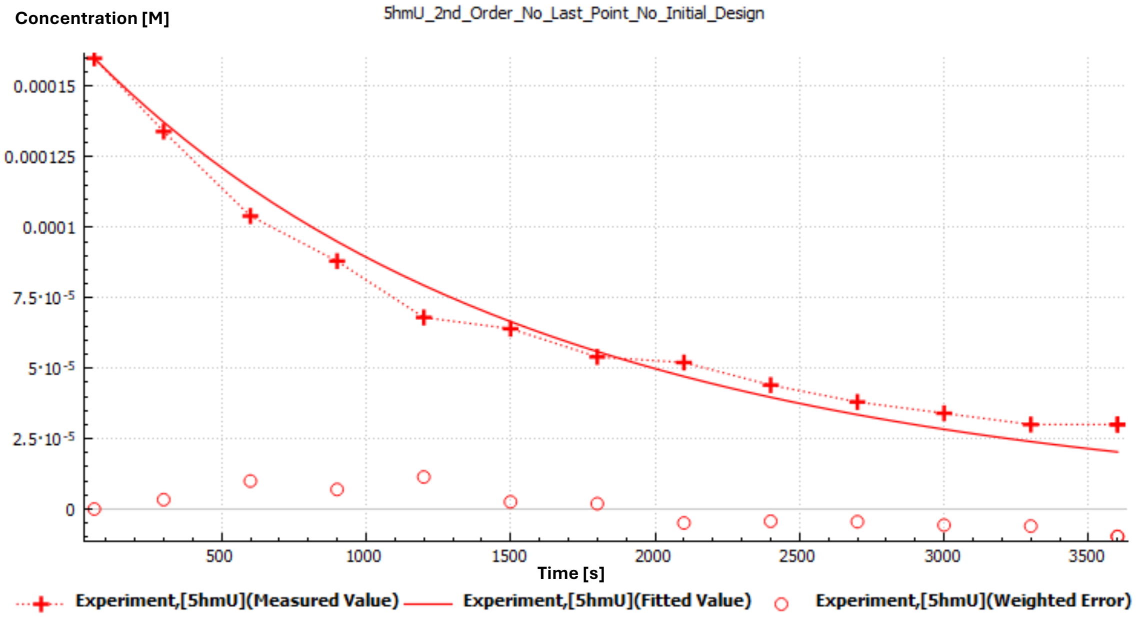

Figure S8. Measured and fitted data for the decay of **5hmU**. All data except the last data point were selected and the designed initial data point was omitted. Substrate concentrations are given in M and reactions times in s. Residuals are shown as open circles.

S4.1.1.4 Inclusion of Oxidant Self-Reaction

As shown in **Figure S8**, the **TM1** oxidant undergoes a slow substrate-independent decomposition reaction. Therefore, we replaced [**TM1**]$_0$ with [**TM1**]. This process was modeled by assuming an effective first-order rate law as shown in eq. (4.4) and yields k_{TM} = 1.754384·10^{-5} ± 0.006146·10^{-5} s^{-1}. Combination of this step with the **5hmU** substrate oxidation as given in eq. (4.3) then yields k(**5hmU**) = 0.655752 ± 0.017548 L·mol^{-1}·s^{-1}. Comparison with the value of k(**5hmU**) = 0.644261 ± 0.018292 L·mol^{-1}·s^{-1} obtained in the absence of oxidant self-reaction, we see that the difference is of the same order as the *RMSD* of the actual measurments (2%).

step 0: **TM1** → **TM2** (4.3)

$$-\frac{d[\mathbf{TM1}]}{dt} = k_{\mathrm{TET}}[\mathbf{TM1}] \tag{4.4}$$

step 1: **5hmU** + **TM1** → **5fU** + **TM2** (4.5)

$$-\frac{d[\mathbf{5hmU}]}{dt} = k(\mathbf{5hmU})[\mathbf{TM1}][\mathbf{5hmU}] \tag{4.6}$$

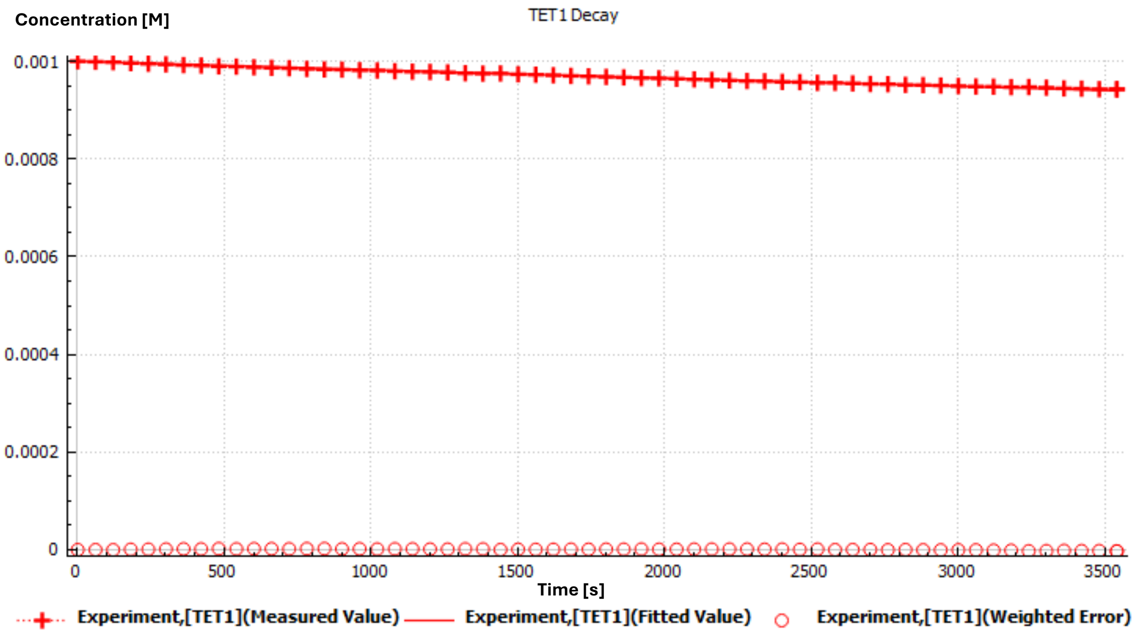

Figure S9. Measured and fitted data for the first-order decay of **TM1** in the absence of substrate. **TM1** concentrations are given in M and reactions times in s. Residuals are shown as open circles.

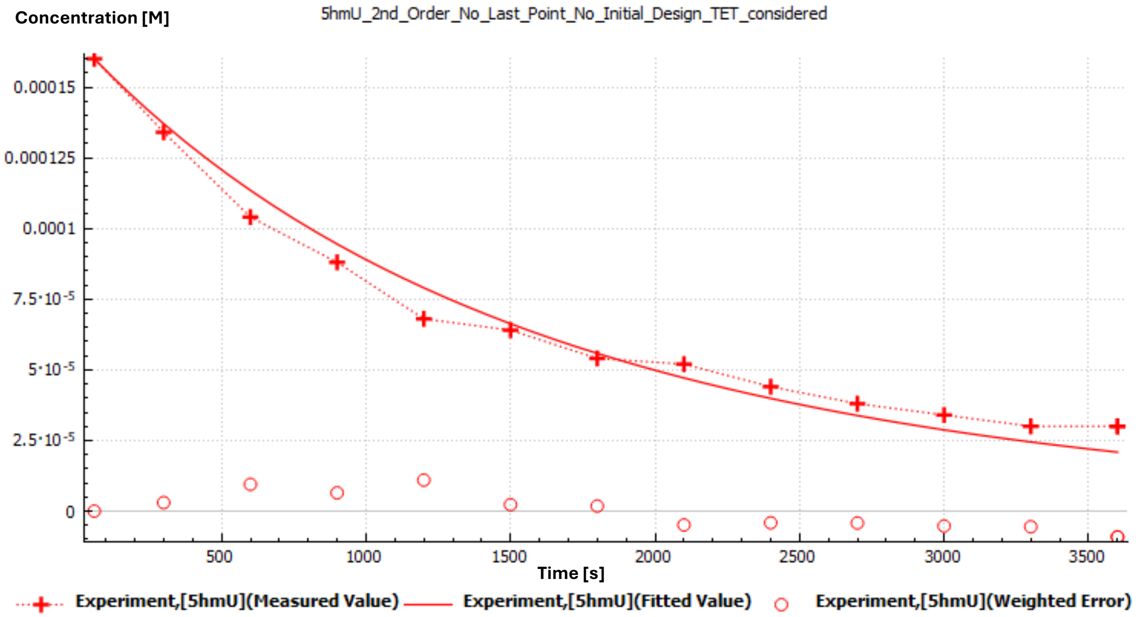

Figure S10. Measured and fitted data for the decay of **5hmU**. All data except the last data point were selected and the designed initial data point was omitted. The self-deactivation of iron-complex was considered. Substrate concentrations are given in M and reactions times in s. Residuals are shown as open circles.

From the various kinetic analyses of the data obtained for the oxidation of **5hmU**, we conclude that omitting the last measurement and the designed initial concentration, and including the self-deactivation of the iron-complex, represents the most robust and accurate method for kinetic analysis. Correspondingly, we applied this method to the oxidation of **5hmC** and **5hm6aU**.

S4.2 Analysis of 5fU Oxidation

S4.2.1 Indirect Determination via 5hmU Oxidation Cascade

Since **5hmU** is sequentially oxidized first to **5fU** and then to **5caU**, we extended the kinetic model to include both steps:

step 0:

$$\mathbf{TM1} \rightarrow \mathbf{TM2} \quad (4.7)$$

$$-\frac{d[\mathbf{TET1}]}{dt} = k_{\mathrm{TM}}[\mathbf{TM1}] \quad (4.8)$$

step 1:

$$\mathbf{5hmU} + \mathbf{TM1} \rightarrow \mathbf{5fU} + \mathbf{TM2} \quad (4.9)$$

$$-\frac{d[\mathbf{5hmU}]}{dt} = \frac{d[\mathbf{5fU}]}{dt} = k(\mathbf{5hmU})[\mathbf{TM1}][\mathbf{5hmU}] \quad (4.10)$$

step 2:

$$\mathbf{5fU} + \mathbf{TM1} \rightarrow \mathbf{5caU} + \mathbf{TM2} \quad (4.11)$$

$$-\frac{d[\mathbf{5fU}]}{dt} = k(\mathbf{5fU})[\mathbf{TM1}][\mathbf{5fU}] \quad (4.12)$$

Since we already determined the rate constant k(**5hmU**) to be 0.655752 $L \cdot mol^{-1} \cdot s^{-1}$ by fitting its concentration profile alone with the optimal method (section S4.1.1.4), we used the value directly without further adjustment. We added the second oxidation step to the reaction kinetics, the formation of **5caU** from **5fU**, as a separate reaction with its own rate constant k(**5fU**). As previously discussed, we omitted the last measurement (t = 86400 s, [**5fU**] = 0.022 mM), used the first measured point (t_0 = 60 s, $[\mathbf{5fU}]_0$ = 0.016 mM) instead of the designed initial concentration (t_0 = 0 s, $[\mathbf{5fU}]_0$ = 0 mM) , and included the self-deactivation of the iron-complex ($[\mathbf{TM1}]_0$ = 1.0 mM, k_{TM} = $1.754384 \cdot 10^{-5}$ s^{-1}). Since there's no observable amount of hydrate formed with an extremely low equilibrium constant ($2.94 \cdot 10^{-4}$ $L \cdot mol^{-1}$), we omitted the equilibrium from **5fU** to **5dhmU**.[2] We optimized the k(**5fU**) to match the formation and consumption profiles of **5fU**, yielding 0.433530 ± 0.006342 $L \cdot mol^{-1} \cdot s^{-1}$.

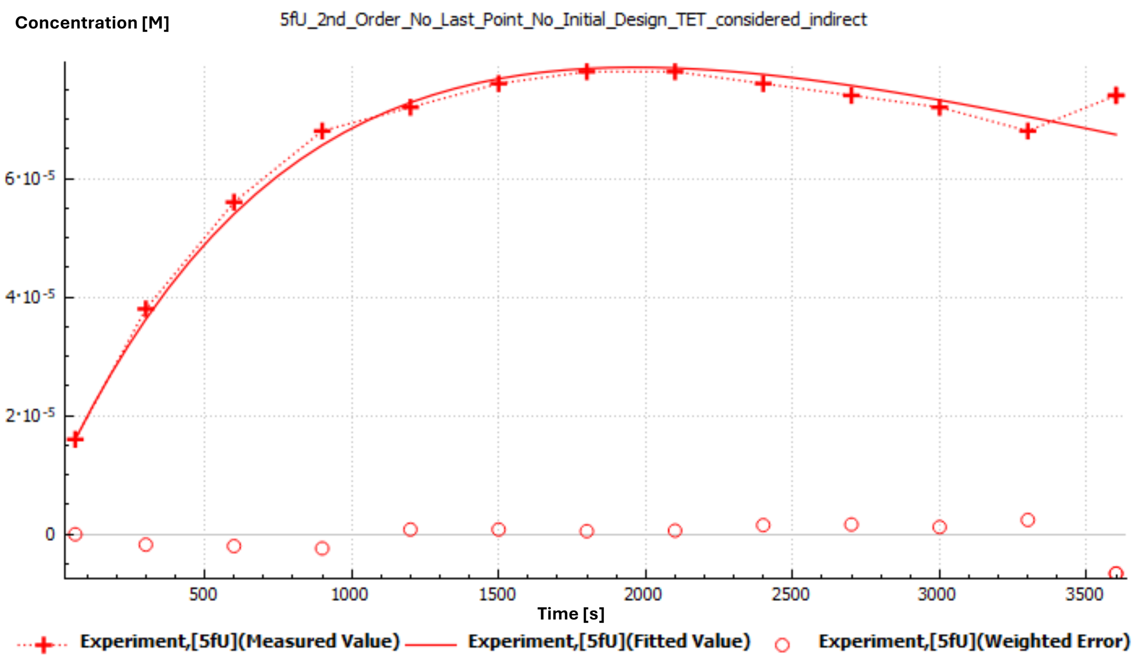

Figure S11. Measured and fitted data for the indirect decay of **5fU** with weighted error in COPSI in a second-order kinetics. All data except the last data point were selected and the designed initial data point was omitted. The self-deactivation of iron-complex was considered. Substrate concentrations are given in M and reactions times in s. Residuals are shown as open circles.

S4.2.2 Direct Determination via 5fU Oxidation

We subsequently measured the direct oxidation of **5fU** to **5caU** and applied the optimal method (t_0 = 45 s, [**5fU**]$_0$ = 0.172 mM, [**TM1**]$_0$ = 1.0 mM, k_{TM} = 1.754384·10^{-5} s^{-1}) to the data points, yielding a rate constant of 0.478630 ± 0.010534 L·mol^{-1}·s^{-1}, with the following mathematic model:

step 0:

$$\mathbf{TM1} \rightarrow \mathbf{TM2} \tag{4.13}$$

$$-\frac{d[\mathbf{TM1}]}{dt} = k_{\mathrm{TET}}[\mathbf{TM1}] \tag{4.14}$$

step 1:

$$\mathbf{5fU} + \mathbf{TM1} \rightarrow \mathbf{5caU} + \mathbf{TM2} \tag{4.15}$$

$$-\frac{d[\mathbf{5fU}]}{dt} = k(\mathbf{5fU})[\mathbf{TM1}][\mathbf{5fU}] \tag{4.16}$$

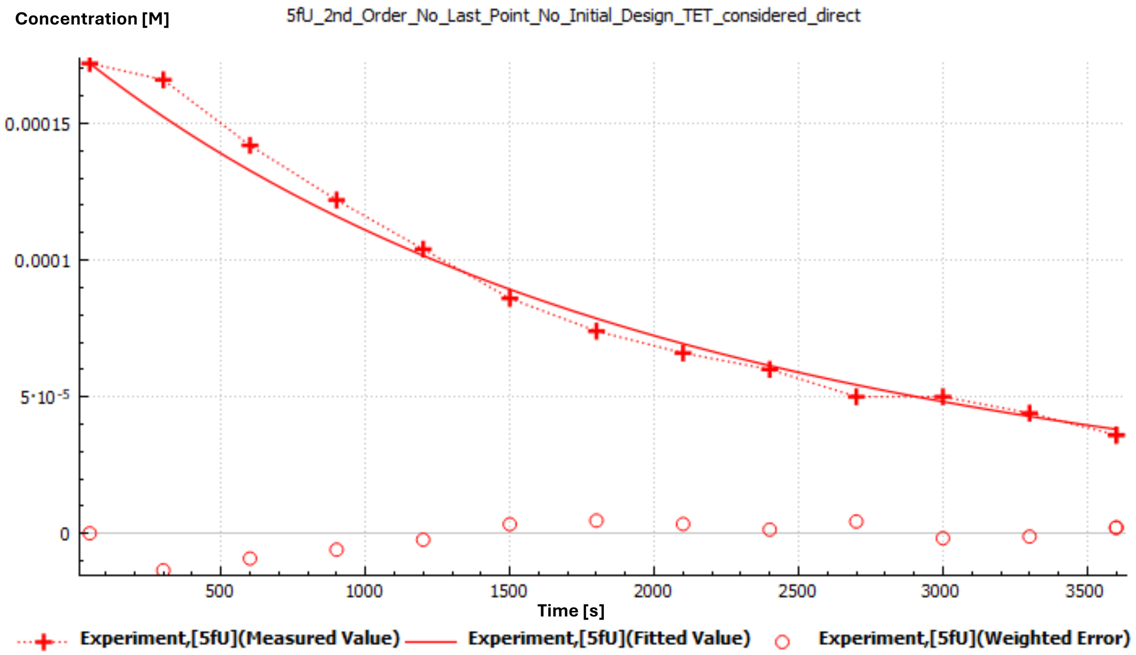

Figure S12. Measured and fitted data for the direct decay of **5fU** with weighted error in COPSI in a second-order kinetics. All data except the last data point were selected and the designed initial data point was omitted. The self-deactivation of iron-complex was considered. Substrate concentrations are given in M and reactions times in s. Residuals are shown as open circles.

In comparison of the value determined with indirect, the rate constant is slightly higher but still remains in a reasonable range. This supports the robustness of our method.

S4.3 Analysis of 5hmC Oxidation

We applied the optimal method (t_0 = 45 s, [**5hmC**]$_0$ = 0.14 mM, [**TM1**]$_0$ = 1.0 mM, k_{TM} = 1.754384·10^{-5} s^{-1}) to the data points of the **5hmC** oxidation yielding a rate constant of k(**5hmC**) = 0.506642 ± 0.010130 L·mol^{-1}·s^{-1}, with the following mathematic model:

step 0: **TM1** → **TM2** (4.17)

$$-\frac{d[\mathbf{TM1}]}{dt} = k_{\mathrm{TM}} \cdot [\mathbf{TM1}] \quad (4.18)$$

step 1: **5hmC** + **TM1** → **5fC** + **TM2** (4.19)

$$-\frac{d[\mathbf{5hmC}]}{dt} = k(\mathbf{5hmC})[\mathbf{TM1}][\mathbf{5hmC}] \quad (4.20)$$

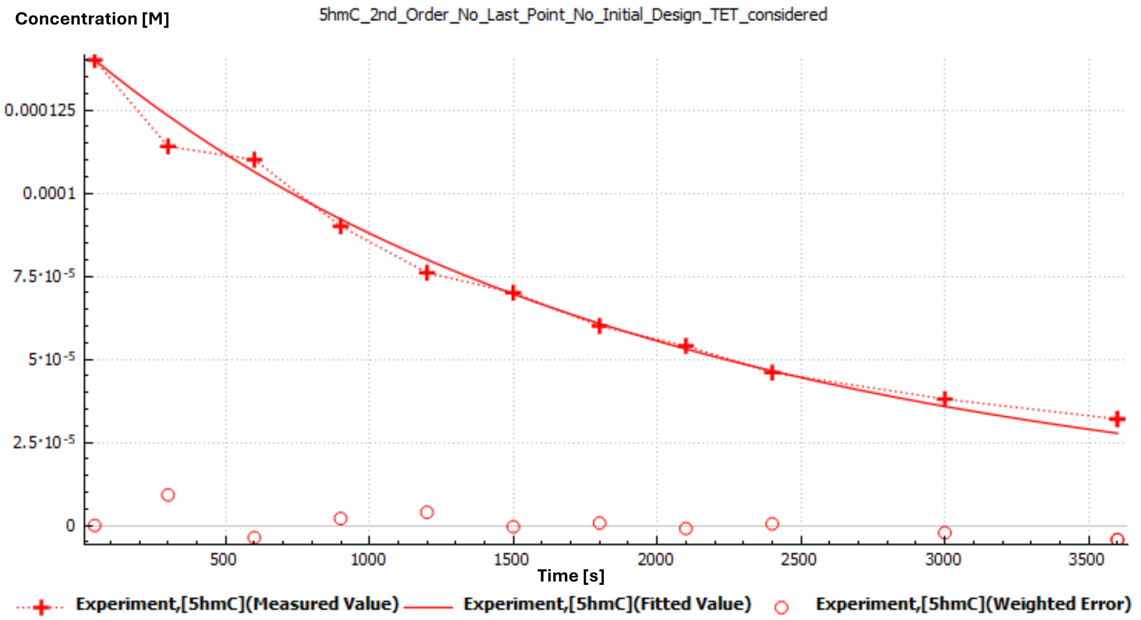

Figure S13. Measured and fitted data for the decay of **5hmC** with weighted error in COPSI in a second-order kinetics. All data except the last data point were selected and the designed initial data point was omitted. The self-deactivation of iron-complex was considered. Substrate concentrations are given in M and reactions times in s. Residuals are shown as open circles.

S4.4 Analysis of 5fC Oxidation

Since 5hmC is sequentially oxidized first to **5fC** and then to **5caC**, we extended the kinetic model to include both steps:

step 0: **TM1 → TM2**

$$-\frac{d[\mathbf{TM1}]}{dt} = k_{\mathrm{TET}} \cdot [\mathbf{TM1}] \tag{4.21}$$

step 1: **5hmC + TM1 → 5fC + TM2**

$$-\frac{d[\mathbf{5hmC}]}{dt} = \frac{d[\mathbf{5fC}]}{dt} = k(\mathbf{5hmC})[\mathbf{TM1}][\mathbf{5hmU}] \tag{4.22}$$

step 2: **5fC + TM1 → 5caC + TM2**

$$-\frac{d[\mathbf{5fC}]}{dt} = k(\mathbf{5fC})[\mathbf{TM1}][\mathbf{5fC}] \tag{4.23}$$

Since we already determined the rate constant *k*(**5hmC**) to be 0.506642 $L\cdot mol^{-1}\cdot s^{-1}$ by fitting its concentration profile alone with the optimal method (**section S4.1.1.4**), we used the value directly without further adjustment. We added the second oxidation step to the reaction kinetics, the formation of **5caC** from **5fC**, as a separate reaction with its own rate constant *k*(**5fC**). As previously discussed, we omitted the last measurement (t = 5400 s, [**5fC**] = 0.026 mM), used the first measured point (t_0 = 45 s, $[5fC]_0$ = 0.01 mM) instead of the designed initial concentration (t_0 = 0 s, $[\mathbf{5fC}]_0$ = 0 mM) , and included the self-deactivation of the iron-complex ($[\mathbf{TM1}]_0$ = 1.0 mM, k_{TM} = $1.754384\cdot10^{-5}$ s^{-1}). For the similar reason as in the case of **5fU**, there's no observable amount of hydrate formed with an even 20 times lower equilibiurm constant than that of **5dhmU** formation ($4.05\cdot10^{-5}$ $L\cdot mol^{-1}$ (derived from limit of detection) and $1.22\cdot10^{-5}$ $L\cdot mol^{-1}$ (derived from limit of quantification)), we omitted the equilibrium from **5fC** to **5dhmC**.[2] We optimized the *k*(**5fC**) to match the formation and consumption profiles of **5fC**, yielding 0.534227 ± 0.030116 $L\cdot mol^{-1}\cdot s^{-1}$.

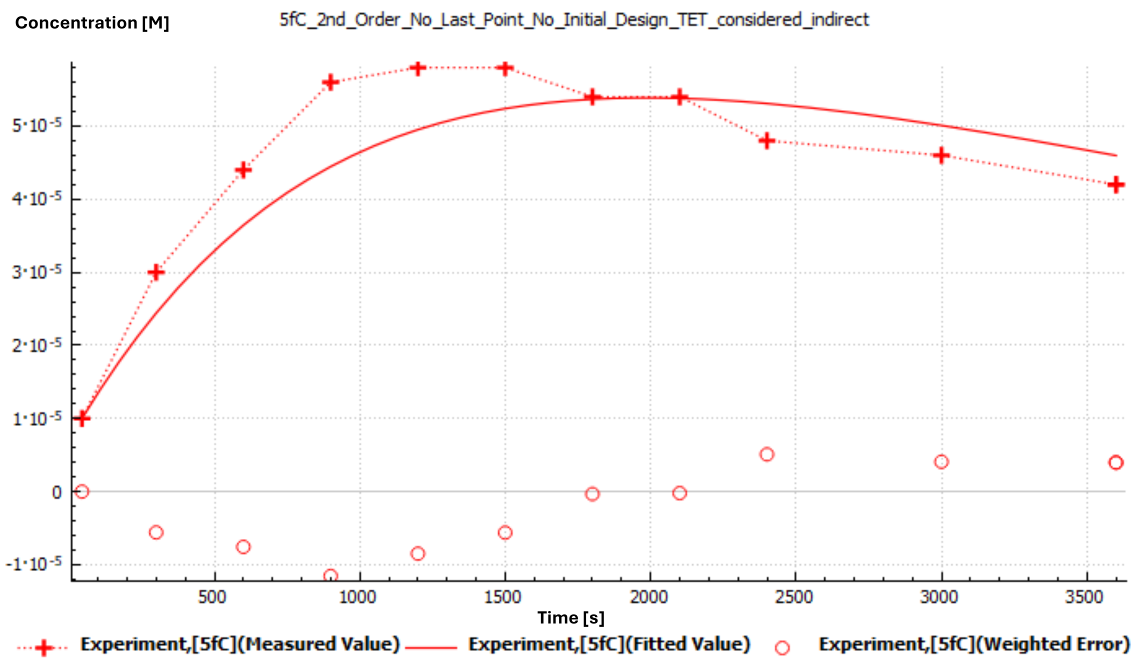

Figure S14. Measured and fitted data for the indirect decay of **5fC** with weighted error in COPSI in a second-order kinetics. All data except the last data point were selected and the designed initial data point was omitted. The self-deactivation of iron-complex was considered. Substrate concentrations are given in M and reactions times in s. Residuals are shown as open circles.

S4.5 Analysis of 5hm6aU Oxidation

We applied the optimal method (t_0 = 25 s, [**5hm6aU**]$_0$ = 0.164 mM, [**TM1**]$_0$ = 1.0 mM, k_{TM} = 1.754384·10^{-5} s^{-1}) to the data points of the **5hm6aU** oxidation yielding a rate constant k(**5hm6aU**) = 1.872846 ± 0.060247 L·mol^{-1}·s^{-1}, with the following kinetic model:

step 0:

$$\mathbf{TM1} \rightarrow \mathbf{TM2} \tag{4.24}$$

$$-\frac{d[\mathbf{TM1}]}{dt} = k_{\mathrm{TET}}[\mathbf{TM1}] \tag{4.25}$$

step 1:

$$\mathbf{5hm6aU} + \mathbf{TM1} \rightarrow \mathbf{5f6aU} + \mathbf{TM2} \tag{4.26}$$

$$-\frac{d[\mathbf{5hm6aU}]}{dt} = k(\mathbf{5hm6aU})[\mathbf{TM1}][\mathbf{5hm6aU}] \tag{4.27}$$

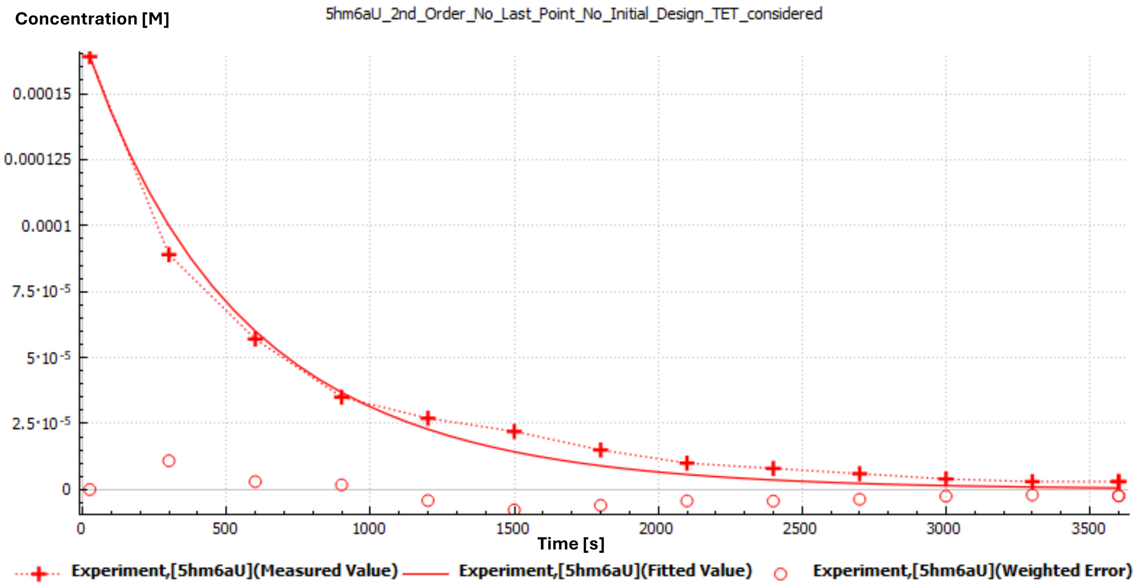

Figure S15. Measured and fitted data for the decay of **5hm6aU** with weighted error in COPSI in a second-order kinetics. All data except the last data point were selected and the designed initial data point was omitted. The self-deactivation of iron-complex was considered. Substrate concentrations are given in M and reactions times in s. Residuals are shown as open circles.

S4.6 Analysis of 5f6aU Oxidation

Since **5hm6aU** is sequentially oxidized first to **5f6aU** and then to **5ca6aU**, we could extend the kinetic model to include both steps. Besides, the formation of **5dhmU** is no longer negligible. The oxidation could happen for both **5fU** and **5dhmU**, however, due to the lack of sufficient data and numerical problems for fitting two variables simultaneously, both oxidation reaction constants could not be reliably determined at the same time. Therefore, we splitted the model into two scenarios: In the first scenario, we assume that only **5fU** goes into further oxidation, while in the second scenario, only **5dhmU** gets oxidized. Therefore, the rate-determining step may shift from direct hydrogen-atom abstraction at the formyl site to that from the geminal diol itself. The formation of the hydrate has been extensively studied by us, and we gathered the equilibrium constants.[2] Using the equilibrium constant for hydration of cytosine and uracil, we found out that uracil tend to build 20-times more hydrates than its cytosine counterparts. Here for **5f6aU**, there's no direct value for the equilibrium constant, however, we could extrapolate it by multiplying 20 to the hydration equilibrium constant K(**1rb5f6aC**) = $4.50\cdot10^{-3}$ L·mol^{-1} of **1rb5f6aC** in the literature[3] to get a rough estimate K(**5f6aU**) ≈ $9\cdot10^{-2}$ L·mol^{-1} (**Figure S16**). This value corresponds to 83% hydrate formation together with the concentration of water [H_2O] = 55.5 mol·L^{-1}, which is in good agreement with the measured average dynamic equilibrium of 88% hydrate (**Table S4**) in our kinetic experiments. One of our pre-experiments was also showed here as an example of the coexisting **5f6aU** and **5dhm6aU** (**Figure S17**). Therefore, we assume that this is the best way to go forward.

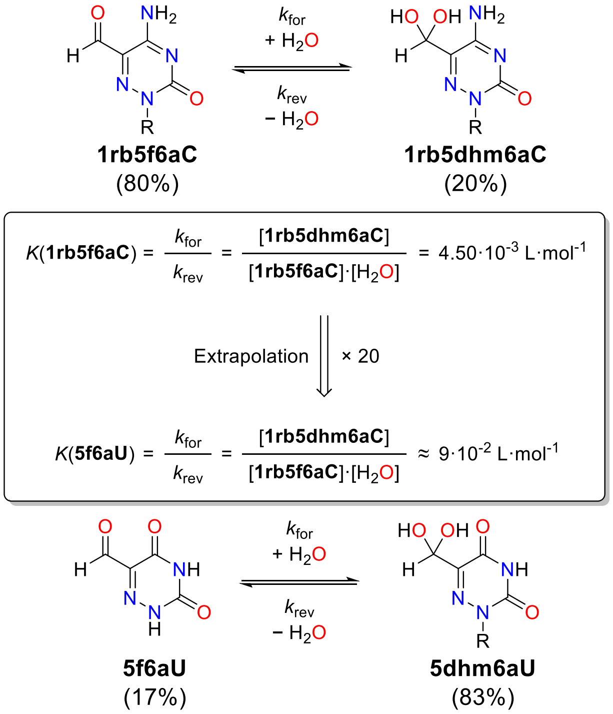

Figure S16. Equilibrium between 5-formyl- (**5f**) and 5-dihydroxy-group (**5dhm**). Together with $[H_2O] = 55.5$ mol·L^{-1}, an equilibrium constant K(**1rb5f6aC**) = $4.50\cdot10^{-3}$ L·mol^{-1} was obtained.[3] Scaling by a factor of 20 yields the extrapolated value K(**5f6aU**) ≈ $9\cdot10^{-2}$ L·mol^{-1} with 83% hydrate.

In dilute solutions, it is practical to consider that the equilibrium between the formyl and hydrate is rather quickly established. Hence, the rate constant was deliberately set to be $k_{for} = 1000$ during the simulation to ensure a quick equilibrium mathematically. Under these assumptions, we now start approaching the two scenarios separately. Whether or not, this equilibrium can be established during the reaction. In order to quantitatively comment on the overall fit accuracy of the two scenarios, we introduced cumulative root mean square deviation, $RMSD_{tot}$, to describe the average observed $RMSD$ for all three species, with N as the number of data points and ΔC as the difference between measured and fitted concentrations (eq. (4.28), eq. (4.29)).

$$RMSD = \sqrt{\frac{1}{N} \cdot \sum_{i=1}^{N} \Delta C^2} \tag{4.28}$$

$$RMSD_{\text{tot}} = \frac{RMSD(\mathbf{5hm6aU}) + RMSD(\mathbf{5f6aU}) + RMSD(\mathbf{5dhm6aU})}{3} \tag{4.29}$$

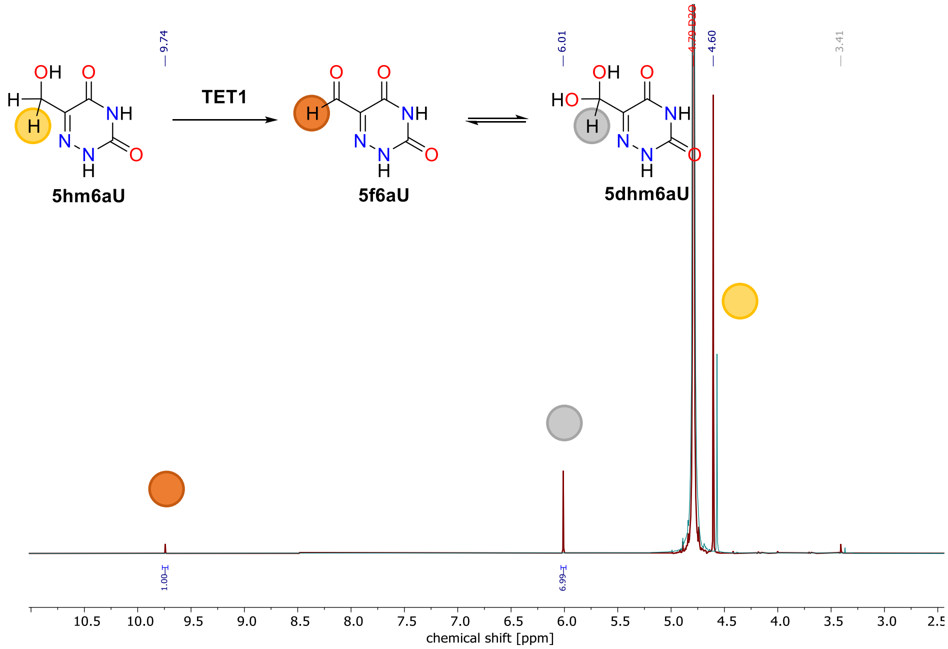

Figure S17. ^{1}H NMR spectra of the obtained products from a reaction of 5hm6aU with **TM1** and reference measurement of **5hm6aU** in D_2O at 298 K. Integration of the aldehyde and hydrate peaks revealed a distribution of 1:7 to **5f6aU** to **5dhm6aU** (88% hydrate). Reaction conditions: [**5hm6aU**] = [**TM1**] = 5 mM, H_2O, 25 °C, 1 h.

S4.6.1 Oxidation only from 5f6aU

For the first scenario, we established the following kinetic model:

step 0:

$$\textbf{TM1} \rightarrow \textbf{TM2} \tag{4.30}$$

$$-\frac{d[\textbf{TM1}]}{dt} = k_{\text{TM}}[\textbf{TM1}] \tag{4.31}$$

step 1:

$$\textbf{5hm6aU} + \textbf{TM1} \rightarrow \textbf{5f6aU} + \textbf{TM2} \tag{4.32}$$

$$-\frac{d[\textbf{5hm6aU}]}{dt} = k(\textbf{5hm6aU})[\textbf{TM1}][\textbf{5hm6aU}] \tag{4.33}$$

step 2:

$$\textbf{5f6aU} + \textbf{H}_2\textbf{O} \leftrightarrow \textbf{5dhm6aU} \tag{4.34}$$

$$-\frac{d[\textbf{5f6aU}]}{dt} = k_{\text{for}}[\textbf{5f6aU}][\textbf{H}_2\textbf{O}] \tag{4.35}$$

$$-\frac{d[\textbf{5dhm6aU}]}{dt} = k_{\text{rev}}[\textbf{5dhm6aU}] \tag{4.36}$$

step 3:

$$\textbf{5f6aU} + \textbf{TM1} \rightarrow \textbf{5caC} + \textbf{TM2} \tag{4.37}$$

$$-\frac{d[\textbf{5f6aU}]}{dt} = k(\textbf{5f6aU})[\textbf{TM1}][\textbf{5f6aU}] \tag{4.38}$$

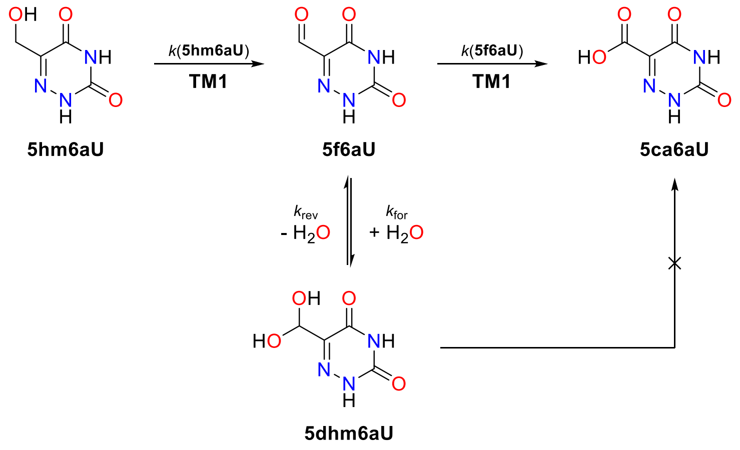

Scheme S9. Reaction model of the oxidation cascade from **5hm6aU** via **5f6aU** to **5ca6aU** for the first scenario.

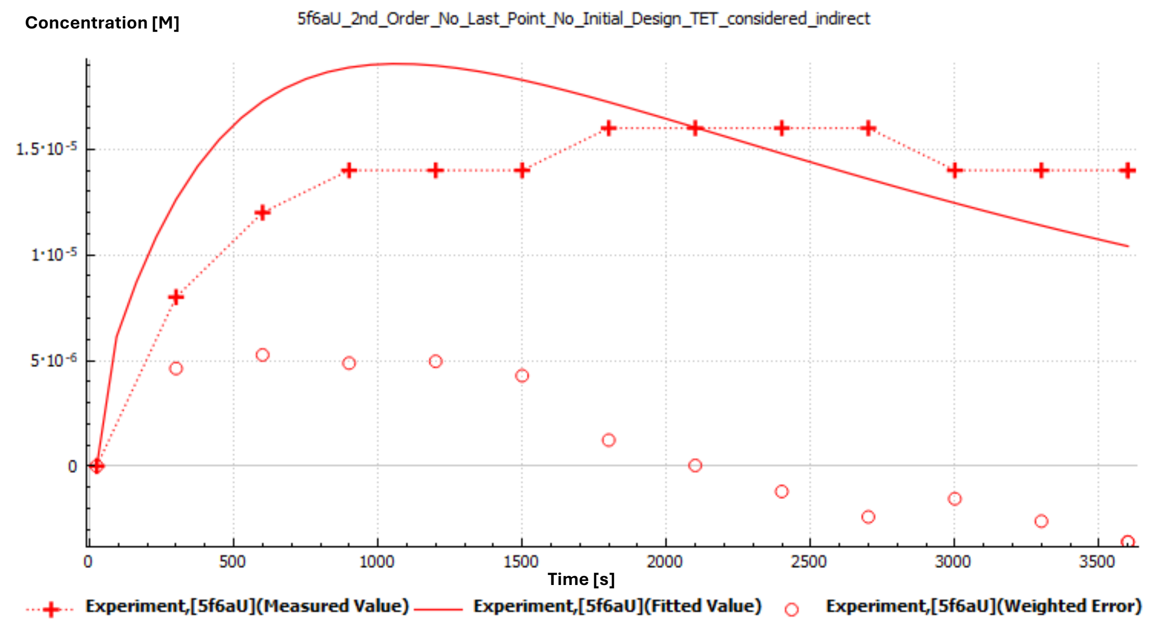

Figure S18. Measured and fitted data for the indirect decay of **5f6aU** with weighted error in COPSI in a second-order kinetics within the scenario where only **5f6aU** is oxidizable. All data except the last data point were selected and the designed initial data point was omitted. The self-deactivation of iron-complex was considered. Substrate concentrations are given in M and reactions times in s. Residuals are shown as open circles.

With values *k*(**5hm6aU**) = 1.872846 $L \cdot mol^{-1} \cdot s^{-1}$ in hand, we can address the oxidation of **5f6aU** to **5ca6aU** as a followup process. This reaction is assumed to follow second-order kinetics with rate constant *k*(**5f6aU**) as shown in eq. (4.38). As previously discussed, we omitted the last measurement (t = 86400 s, [**5f6aU**] = 0 mM), used the first measured point (t_0 = 25 s, [**5f6aU**]$_0$ = 0 mM) instead of the designed initial concentration (t_0 = 0 s, [**5f6aU**]$_0$ = 0.2 mM), and included the self-deactivation of the iron-complex ([**TM1**]$_0$ = 1.0 mM, k_{TET} = $1.754384 \cdot 10^{-5}$ s^{-1}). Fitting the formation and consumption profiles of **5f6aU**, we obtain *k*(**5f6aU**) = 2.861119 ± 0.256944 $L \cdot mol^{-1} \cdot s^{-1}$ with the indirect method within the first scenario. We also have *RMSD*(**5hm6aU**) = 0.000005 $mol \cdot L^{-1}$, *RMSD*(**5f6aU**) = 0.000003 $mol \cdot L^{-1}$, and *RMSD*(**5dhm6aU**) = 0.000030 $mol \cdot L^{-1}$, giving a cumulative $RMSD_{tot}$ = 0.000013 $mol \cdot L^{-1}$.The fitted curve deviated to a certain extent from the data points of **5f6aU** whose concentration stayed constant, suggesting that our kinetic model doesn't fully represent the whole situation of the **5f6aU** oxidation (**Figure S18**).

S4.6.2 Oxidation only from 5dhm6aU

Similarly, we established the following kinetic model for the second scenario:

step 0: $$\textbf{TM1} \rightarrow \textbf{TM2} \tag{4.39}$$

$$-\frac{d[\mathbf{TM1}]}{dt} = k_{\mathrm{TM}}[\mathbf{TM1}] \tag{4.40}$$

step 1: $$\textbf{5hm6aU} + \textbf{TM1} \rightarrow \textbf{5f6aU} + \textbf{TM2} \tag{4.41}$$

$$-\frac{d[\mathbf{5hm6aU}]}{dt} = k(\mathbf{5hm6aU})[\mathbf{TM1}][\mathbf{5hm6aU}] \tag{4.42}$$

step 2: $$\textbf{5f6aU} + \mathbf{H_2O} \leftrightarrow \textbf{5dhm6aU} \tag{4.43}$$

$$-\frac{d[\mathbf{5f6aU}]}{dt} = k_{\mathrm{for}}[\mathbf{5f6aU}][\mathbf{H_2O}] \tag{4.44}$$

$$-\frac{d[\mathbf{5dhm6aU}]}{dt} = k_{\mathrm{rev}}[\mathbf{5dhm6aU}] \tag{4.45}$$

step 3: $$\textbf{5dhm6aU} + \textbf{TM1} \rightarrow \textbf{5caC} + \textbf{TM2} + \mathbf{H_2O} \tag{4.46}$$

$$-\frac{d[\mathbf{5dhm6aU}]}{dt} = k(\mathbf{5dhm6aU})[\mathbf{TM1}][\mathbf{5f6aU}] \tag{4.47}$$

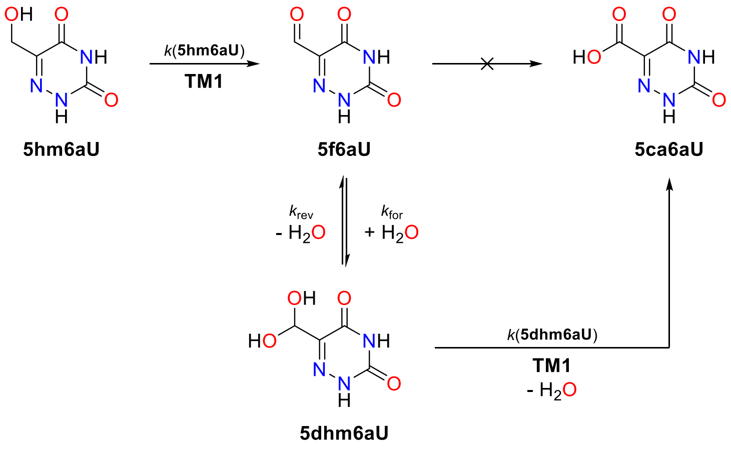

Scheme S10. Reaction model of the oxidation cascade from **5hm6aU** via **5dhm6aU** to **5ca6aU** for the second scenario.

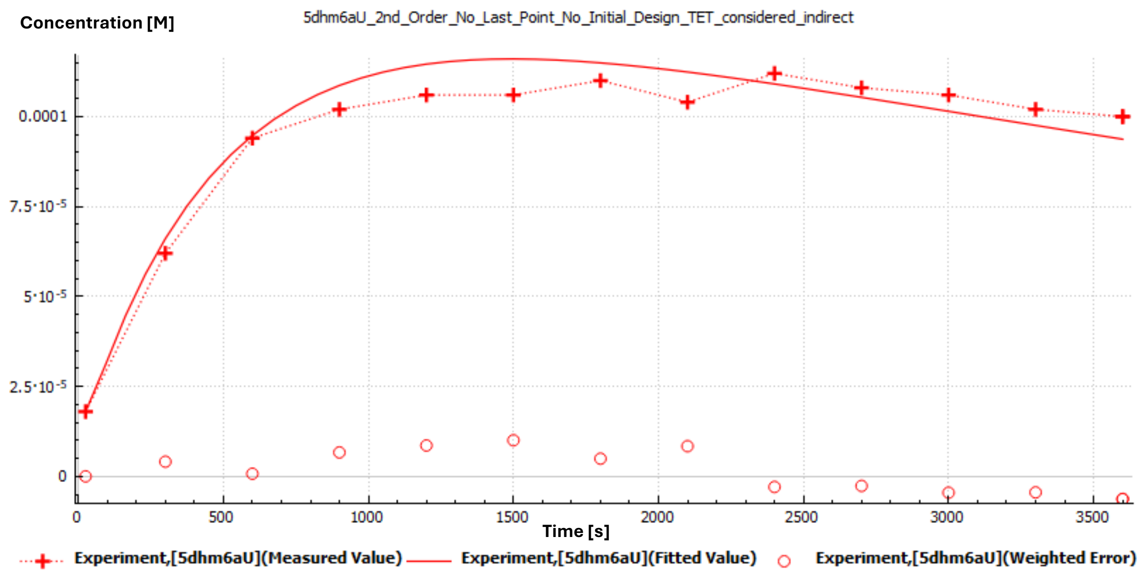

Figure S19. Measured and fitted data for the indirect decay of **5dhm6aU** with weighted error in COPSI in a second-order kinetics within the scenario where only **5dhm6aU** is oxidizable. All data except the last data point were selected and the designed initial data point was omitted. The self-deactivation of iron-complex was considered. Substrate concentrations are given in M and reactions times in s. Residuals are shown as open circles.

With a similar approach, this reaction is assumed to follow second-order kinetics with rate constant *k*(**5dhm6aU**) as shown in eq.(4.47). As previously discussed, we omitted the last measurement (t = 86400 s, [**5dhm6aU**] = 0.008 mM), used the first measured point (t_0 = 25 s, [**5dhm6aU**]$_0$ = 0.018 mM), and included the self-deactivation of the iron-complex ([**TM1**]$_0$ = 1.0 mM, k_{TM} = 1.754384·10^{-5} s^{-1}). Fitting the formation and consumption profiles of **5dhm6aU**, we obtain *k*(**5dhm6aU**) = 0.241119 ± 0.010390 L·mol^{-1}·s^{-1} with the indirect method within the second scenario. We also have *RMSD*(**5hm6aU**) = 0.000005 mol·L^{-1}, *RMSD*(**5f6aU**) = 0.000007 mol·L^{-1}, and RMSD(**5dhm6aU**) = 0.000006 mol·L^{-1}, giving a cumulative $RMSD_{tot}$ = 0.000006 mol·L^{-1}, which is significantly lower than that in the first scenario. The fitted curve correlates quite satisfactorily with the data points of **5dhm6aU**, suggesting that the hydration of **5f6aU** is important for the whole oxidation cascade (**Figure S19**).

S4.6.3 Simultaneous Oxidation from 5f6aU and 5dhm6aU

Next we combined the two known kinetic constants determined by assuming single oxidation path and allowed a simultaneous oxidation through both pathways in our kinetic model:

step 0: **TM1 → TM2** (4.48)

$$-\frac{d[\mathbf{TM1}]}{d\mathrm{t}} = k_{\mathrm{TET}}[\mathbf{TM1}] \quad (4.49)$$

step 1: **5hm6aU** + **TM1** → **5f6aU** + **TM2** (4.50)

$$-\frac{d[\mathbf{5hm6aU}]}{d\mathrm{t}} = k(\mathbf{5hm6aU})[\mathbf{TM1}][\mathbf{5hm6aU}] \quad (4.51)$$

step 2: **5f6aU** + $\mathbf{H_2O}$ ↔ **5dhm6aU** (4.52)

$$-\frac{d[\mathbf{5f6aU}]}{d\mathrm{t}} = k_{\mathrm{for}}[\mathbf{5f6aU}][\mathbf{H_2O}] \quad (4.53)$$

$$-\frac{d[\mathbf{5dhm6aU}]}{d\mathrm{t}} = k_{\mathrm{rev}}[\mathbf{5dhm6aU}] \quad (4.54)$$

step 3: **5f6aU** + **TM1** → **5caC** + **TM2** (4.55)

$$-\frac{d[\mathbf{5f6aU}]}{d\mathrm{t}} = k(\mathbf{5f6aU})[\mathbf{TM1}][\mathbf{5f6aU}] \quad (4.56)$$

step 4: **5dhm6aU** + **TM1** → **5caC** + **TM2** + $\mathbf{H_2O}$ (4.57)

$$-\frac{d[\mathbf{5dhm6aU}]}{d\mathrm{t}} = k(\mathbf{5dhm6aU})[\mathbf{TM1}][\mathbf{5f6aU}] \quad (4.58)$$

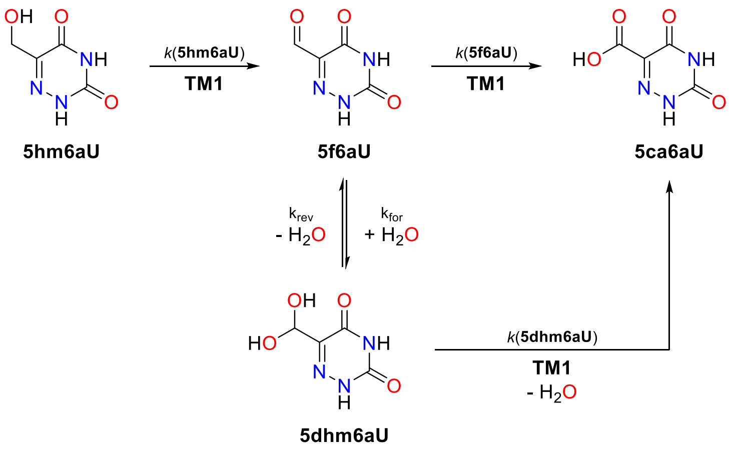

Scheme S11. Reaction model of the oxidation cascade from **5hm6aU** via **5dhm6aU** to **5ca6aU** for the second scenario.

Fitting the profiles of **5dhm6aU** and **5f6aU** simultaneously with the same method, we obtain the following unrealistic *k*(**5dhm6aU**) = 0.005144 ± 206.503819 $L \cdot mol^{-1} \cdot s^{-1}$ and *k*(**5f6aU**) = 1.225860 ± 1031.701323 $L \cdot mol^{-1} \cdot s^{-1}$ values. Switching the methods from the default "Evolutionary Programming" to "Differential Evolution" or "Levenberg - Marquardt" for the fitting still gave similar results with high deviation.

S4.7 Simulation of Oxidation Cascade

S4.7.1 5hmU Oxidation

With the known start condition and the determined rate constants for the oxidation of **5hmU** to **5fU** and **5fU** to **5caU**, we could simulate the concentration development of the nucleobases with the model previously discussed (S4.2.1). Since there are two different values for the oxidation of **5fU** to **5caU**, we simulated the data correspondingly twice (**Figure S20**). We then overlayed the simulated data with our original measured data (**Figure S21**).

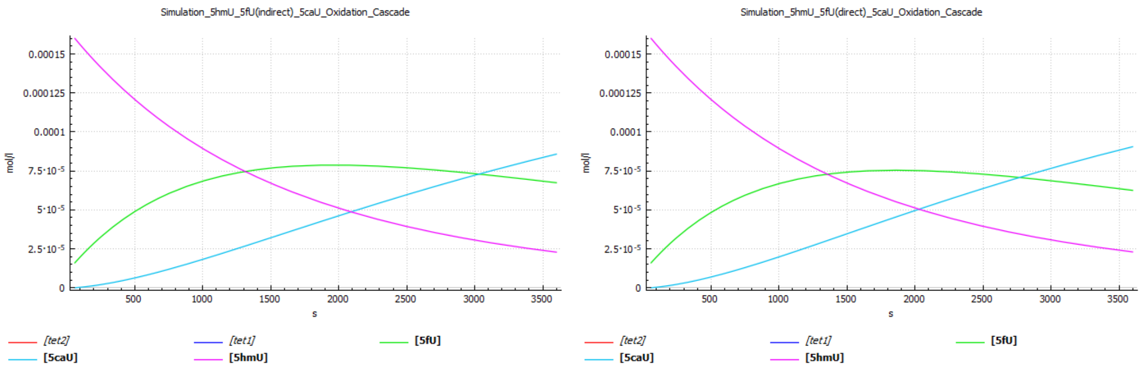

Figure S20. Simulated data for the oxidation cascade of **5hmU** with determined rate constants and known starting concentrations. The used oxidation reaction constant for **5fU** to **5caU** on the left were the indirectly determined, while the constants on the right were the directly determined.

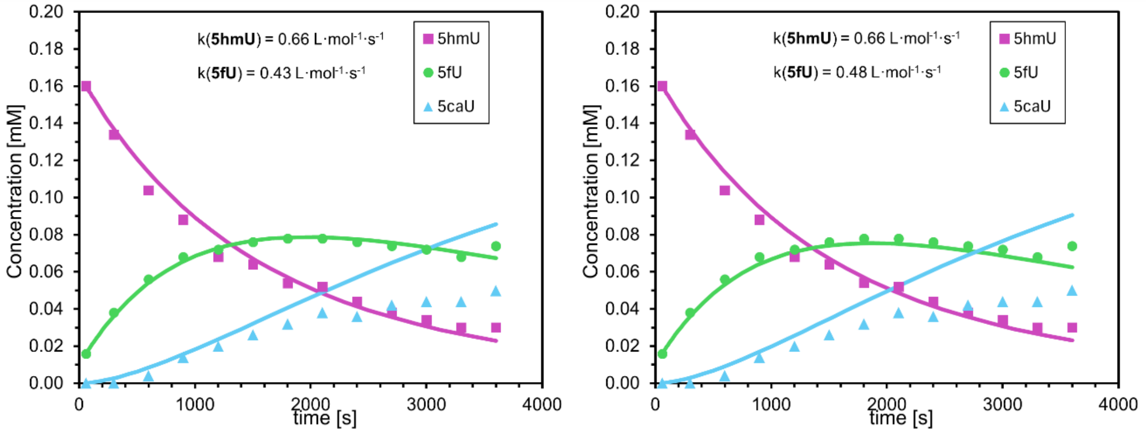

Figure S21. Overlay of simulated data with original data for the oxidation cascade of **5hmU** with determined rate constants and known starting concentrations. The used oxidation reaction constant for **5fU** to **5caU** on the left was the indirectly determined, while the constant on the right was the directly determined.

Both fits are satisfactory except for **5caU**, which could be explained by **5caU** product loss through column chromatography workup. The difference between the cases is minimal, but it's distinguishable that the indirectly determined rate constant fit better to the measured oxidation cascade.

S4.7.2 5hmC Oxidation

With the same procedure, we simulated the data correspondingly (**Figure S20**). We then overlayed the simulated data with our original measured data (**Figure S21**).

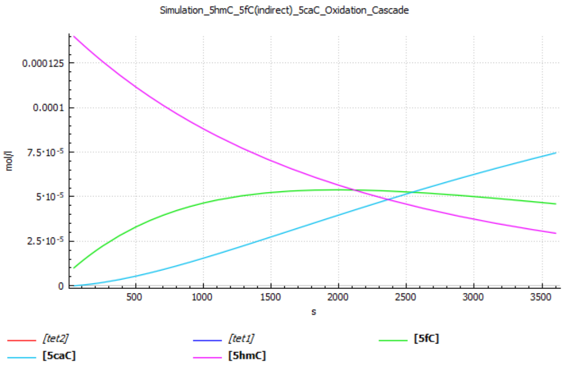

Figure S22. Simulated data for the oxidation cascade of **5hmC** with determined rate constants and known starting concentrations. The used oxidation reaction constant for **5fU** to **5caU** was the indirectly determined.

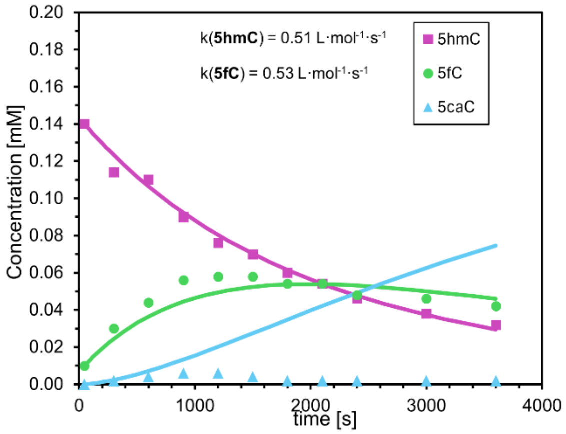

Figure S23. Overlay of simulated data with original data for the oxidation cascade of **5hmU** with determined rate constants and known starting concentrations. The used oxidation reaction constant for **5fC** to **5caC** was determined indirectly.

It is pretty clear that both fits look quite good except for **5caC**, which could be explained by loss of this base during workup.

S4.7.3 5hm6aU Oxidation

With the same procedure, we simulated the data correspondingly (**Figure S20**). We then overlayed the simulated data with our original measured data (**Figure S21**).

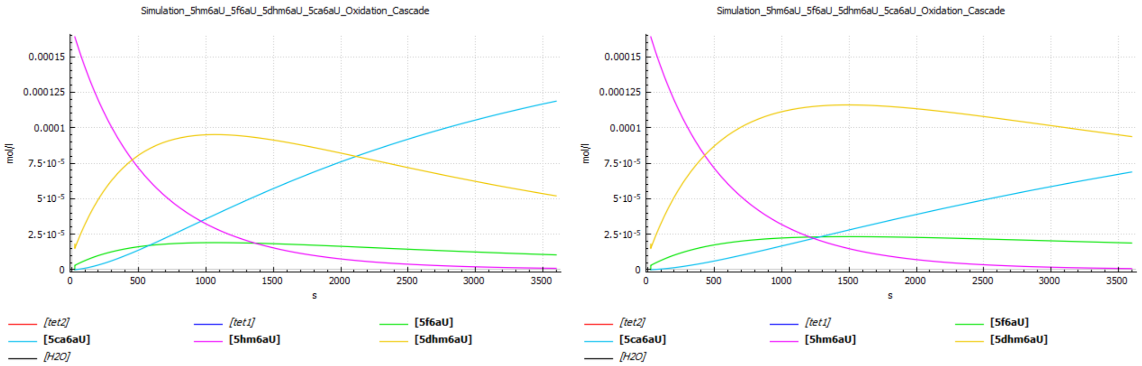

Figure S24. Simulated data for the oxidation cascade of **5hm6aU** with determined rate constants and known starting concentrations. The first scenario (only **5f6aU** oxidizable) is on the left and the second scenario (only **5dhm6aU** oxidizable) is on the right.

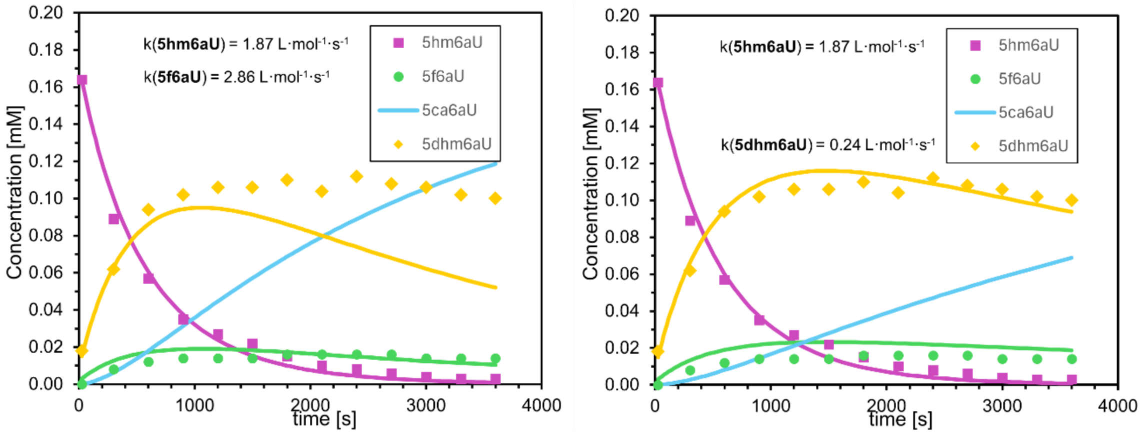

Figure S25. Overlay of simulated data with original data for the oxidation cascade of **5hm6aU** with determined rate constants and known starting concentrations. The first scenario (only **5f6aU** oxidizable) is on the left and the second scenario (only **5dhm6aU** oxidizable) is on the right.

It is quite clear that the fit for **5f6aU** in the first scenario is better than that of second scenario, however, the fit for **5dhm6aU** is awful in the first scenario. Despite the minor deviation for the fit of **5f6aU** in the second scenario, it remains the better fit overall, suggesting that the oxidation mostly proceeds through the hydrate. Since there's no data available for the formation of **5ca6aU** likely due to loss on the column, it was only simulated without corresponding raw data as reference.

S5. Results and Discussion

S5.1 Overview of Determined Rate Constants

Table S5. Rate constants (k) and their standard deviation (δk) for the side-chain oxidation of different nucleobases.

Base	Cascade	Step No.	Measurement	k $L \cdot mol^{-1} \cdot s^{-1}$	δk $L \cdot mol^{-1} \cdot s^{-1}$
**5hmU**	**5hm → 5f → 5ca**	1	direct	0.66	0.02
**5fU**		2	indirect	0.48	0.01
**5fU**	**5f → 5ca**	1	direct	0.43	0.01
**5hmC**	**5hm → 5f→ 5ca**	1	direct	0.51	0.01
**5fC**		2	indirect	0.53	0.03
**5hm6aU**	**5hm → 5f/5dhm→ 5ca**	1	direct	1.87	0.06
**5f6aU**		2	indirect	2.86	0.26
**5dhm6aU**		2	indirect	0.24	0.01

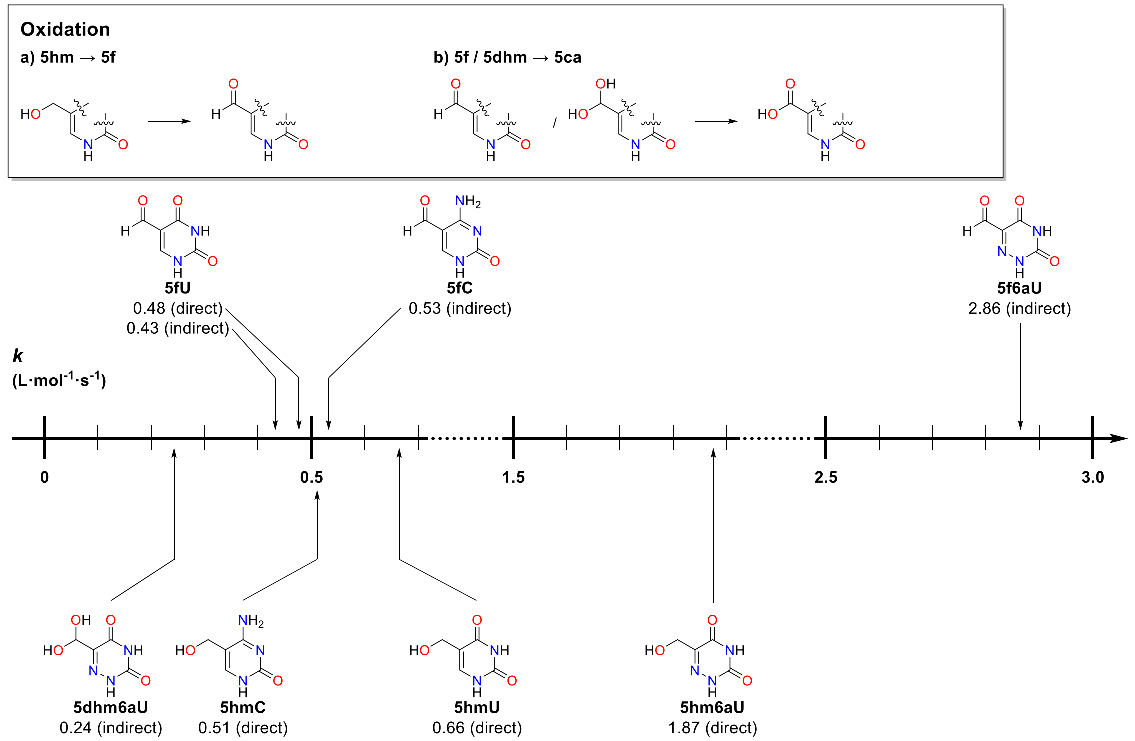

Figure S26. Second-order rate constants ($L \cdot mol^{-1} \cdot s^{-1}$) for the single-step oxidation of **(a)** hydroxymethyl- (**5hm**) and **(b)** formyl- (**5f**) or dihydroxyl- (**5dhm**) substituted nucleobases by our iron(IV)-oxo complex (**TM1**).

We summarize the experimentally determined rate constants *k* for the oxidation step converting **5hm**- to **5f**-, and **5f**- to **5ca**-moiety in a graphical manner (**Figure S26**). Each nucleobase exhibits a characteristic second-order rate constant, reflecting how quickly they react with the iron complex for the oxidation. Specifically, **5hmC** and **5hmU** show moderate reactivity, with direct oxidation rates of approximately 0.50 and 0.66 $L \cdot mol^{-1} \cdot s^{-1}$ respectively. By contrast, **5hm6aU** undergoes the fastest oxidation to its **5f**-derivative, at 1.87 $L \cdot mol^{-1} \cdot s^{-1}$, indicating that nitrogen substitution at the C6 (6-aza-substitution) can significantly accelerate the transformation, which is likely due to nitrogen's electron withdrawing nature. For the oxidation of **5f** species, we assume that **TM1** attacks the formyl group without prior nucleophilic addition of water, abstracting the necessary hydrogen and facilitating the subsequent oxidation steps toward **5ca** formation. Two different pathways for forming **5fU** were also measured, with a direct method (0.48 $L \cdot mol^{-1} \cdot s^{-1}$) as well as the indirect method (0.43 $L \cdot mol^{-1} \cdot s^{-1}$), where the slight difference in k may arise from experiment procedures. The oxidation of **5f6aU** within the assumption that **5dhm6aU** is not oxidizable would be the fastest among all measurements (2.86 $L \cdot mol^{-1} \cdot s^{-1}$), but it's not as reliable as in the case that the oxidation solely proceeds through **5dhm6aU** with an overall better fit and giving a rate constants of 0.24 $L \cdot mol^{-1} \cdot s^{-1}$, which is relatively slow. Nevertheless, these data underscore how small changes in nucleobase structure can markedly alter reaction kinetics. Electron-withdrawing substituents modulate the susceptibility of the **5hm** group to undergo oxidation, while the inherent stability of intermediates may also have an influence. Thus, among the nucleobases tested, **5hm6aU** is the most rapidly oxidized to its **5f** form, whereas **5hmC** and **5hmU** proceed at more moderate but still comparable rates.

S5.2 Correlation Between BDE and Oxidation Rate Constants

A key mechanistic question in the oxidation of **5f** moiety by our iron complex is whether the rate-determining step involves formation of the water hydrate. To probe this, we calculated the bond dissociation energies (BDEs) for the relevant C-H bonds in a series of nucleobases, including **5hmC**, **5hmU**, **5hm6aU**, **5fC**, **5fU**, **5f6aU**, **5dhmC**, **5dhmU**, and **5dhm6aU**. These theoretical BDE values provide a quantitative measure of how readily each substrate can undergo hydrogen abstraction. The averaged value of the direct and indirect method for **5fU** oxidation was used as the rate constant *k*(**5fU**), which is 0.46 (**Table S7**). For the other species, there is only one value for each. However, since the equilibrium constants for hydration of **5fC** and **5fU** are extremely low, we didn't manage to get reliable results to facilitate direct analysis. Therefore, there's still one scenario to be discussed, namely what would the rate constants look like if the oxidation of the **5f** moiety solely goes through the **5dhm** hydrate form.

Table S6. Comparison of calculated BDEs and observed reaction rates.

**Base**	**BDE** kJ·mol^{-1}	***k*** L·mol^{-1}·s^{-1}
**5hmU**	360.0	0.66
**5fU**	394.7	0.46
**5hmC**	367.4	0.51
**5fC**	401.0	0.53
**5hm6aU**	337.7	1.87
**5f6aU**	395.4	2.86
**5dhm6aU**	313.8	0.24

Similar to the previously discussed scenario with **5dhm6aU** (section S4.6.2) that envisions the **5f** moiety first undergoing hydration to form a geminal diol (**5dhm**) intermediate followed by oxidation by **TM1**, we established the following kinetic model:

step 0: **TM1** → **TM2** (5.1)

$$-\frac{d[\mathbf{TM1}]}{dt} = k_{\mathrm{TM}}[\mathbf{TM1}] \quad (5.2)$$

step 1: **5hm** + **TM1** → **5f** + **TM2** (5.3)

$$-\frac{d[\mathbf{5hm}]}{dt} = k(\mathbf{5hm})[\mathbf{TM1}][\mathbf{5hm}] \quad (5.4)$$

step 2: **5f** + $\mathbf{H_2O}$ ↔ **5dhm** (5.5)

$$-\frac{d[\mathbf{5f}]}{dt} = k_{\mathrm{for}}[\mathbf{5f}][\mathbf{H_2O}] \quad (5.6)$$

$$-\frac{d[\mathbf{5dhm}]}{dt} = k_{\mathrm{rev}}[\mathbf{5dhm}] \quad (5.7)$$

step 3: **5dhm** + **TM1** → **5caC** + **TM2** + $\mathbf{H_2O}$ (5.8)

$$-\frac{d[\mathbf{5dhm}]}{dt} = k(\mathbf{5dhm})[\mathbf{TM1}][\mathbf{5dhm}] \quad (5.9)$$

For the equilibrium constant of **5fC**, the value K(**5fC**) = $2.64\cdot10^{-5}$ was used as an average of $4.05\cdot10^{-5}$ (from limit of detection) and $1.22\cdot10^{-5}$ (from limit of quantification), while for **5fU**, we used the constant K(**5fU**) = $2.94\cdot10^{-4}$ directly.[2] We also consider rapid equilibrium between the formyl and hydrate and set the rate constant to be k_{for} = 1000 during the simulation to mathematically ensure a quick equilibrium. After applying the kinetic model, we gathered a rate constant for **5dhmU** with 26.165608 ± 0.402672 $\mathrm{L\cdot mol^{-1}\cdot s^{-1}}$ for the indirect method (**Figure S27**) and 29.159929 ± 0.738406 $\mathrm{L\cdot mol^{-1}\cdot s^{-1}}$ for the direct method (**Figure S28**), the average of which gives k(**5dhmU**) = 27.662769 $\mathrm{L\cdot mol^{-1}\cdot s^{-1}}$. With the same procedure, we gathered the rate constants k(**5dhmC**) = 364.229670 ± 20.649516 $\mathrm{L\cdot mol^{-1}\cdot s^{-1}}$ (**Figure S29**). These extremely high values reflect that the oxidation through the hydrates would be rather unlikely.

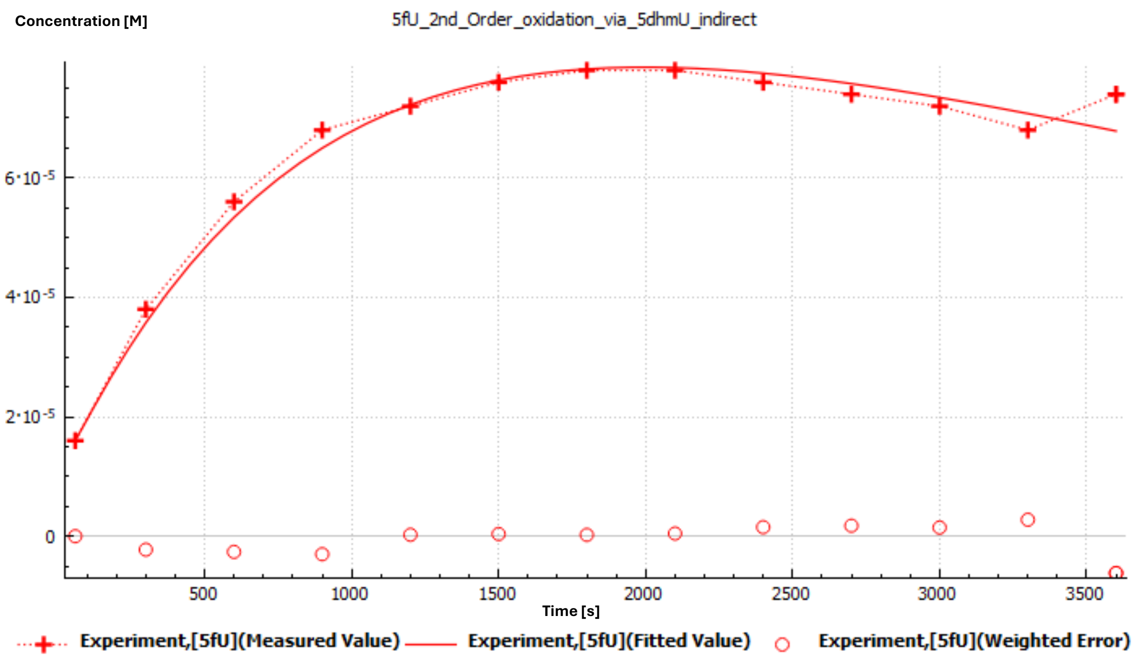

Figure S27. Measured and fitted data for the indirect decay of **5fU** with weighted error in COPSI in a second-order kinetics via formation of **5dhmU**. All data except the last data point were selected and the designed initial data point was omitted. The self-deactivation of iron-complex was considered. Substrate concentrations are given in M and reactions times in s. Residuals are shown as open circles.

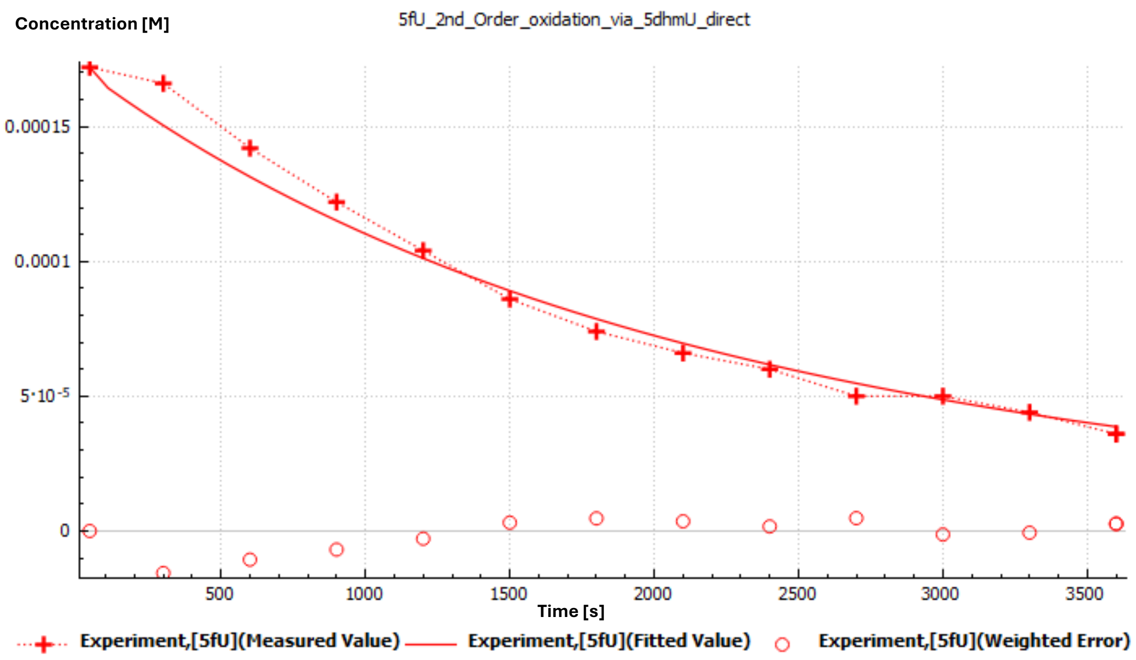

Figure S28. Measured and fitted data for the direct decay of **5fU** with weighted error in COPSI in a second-order kinetics via formation of **5dhmU**. All data except the last data point were selected and the designed initial data point was omitted. The self-deactivation of iron-complex was considered. Substrate concentrations are given in M and reactions times in s. Residuals are shown as open circles.

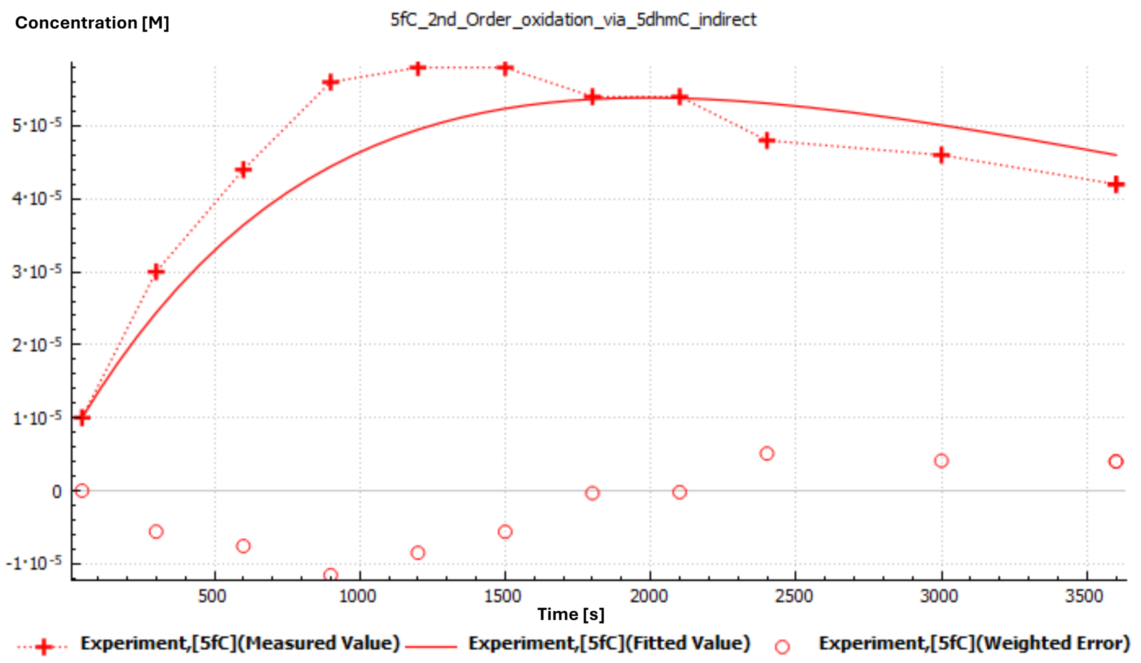

Figure S29. Measured and fitted data for the indirect decay of **5fC** with weighted error in COPSI in a second-order kinetics. All data except the last data point were selected and the designed initial data point was omitted. The self-deactivation of iron-complex was considered. Substrate concentrations are given in M and reactions times in s. Residuals are shown as open circles.

Table S7. Comparison of calculated BDEs and observed reaction rates.

Base	BDE<br>kJ·mol^{-1}	k<br>L·mol^{-1}·s^{-1}
**Previous study**[4]		
**1mC**	414.8	0.06
**1mU**	416.0	0.06
**5mC**	387.3	0.18
**1,5dmC**	386.5	0.30
**1,5dmU**	385.7	0.44
**5mU**	383.1	0.63
**5m*i*C**	379.4	1.44
**This study**		
**5hmU**	360.0	0.66
**5fU**	394.7	0.46
**5dhmU**	354.1	27.66
**5hmC**	367.4	0.51
**5fC**	401.0	0.53
**5dhmC**	322.1	364.23
**5hm6aU**	337.7	1.87
**5f6aU**	395.4	2.86
**5dhm6aU**	313.8	0.24

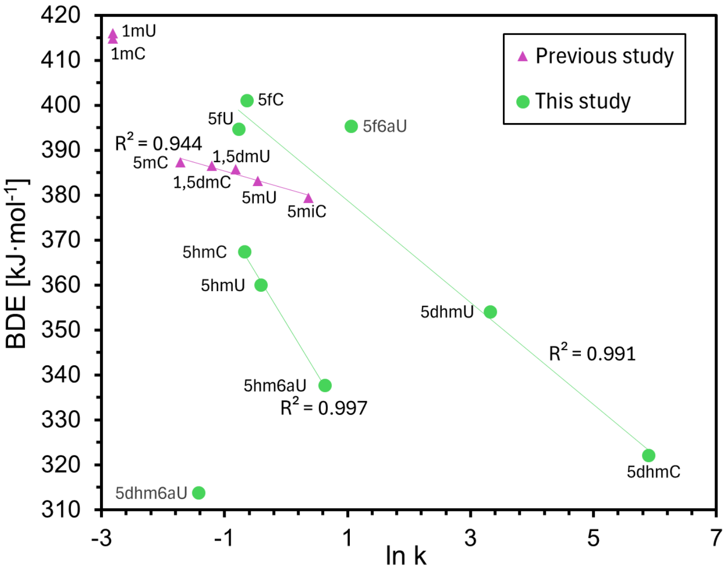

Figure S30. Correlation of the experimentally determined rate constants for **5hm** to **5f** and indirect oxidation of **5f** to **5ca** via **5dhm** with the calculated bond dissociation energies (BDEs) determined in this study (green), in comparison with our previous study (pink).[4]

With the determined rate constants for the diol, we plotted the BDEs against the corresponding rate constants (**Figure S30**). Our calculated bond dissociation energies (BDEs) suggest that hydrogen-atom abstraction from the **5f** moiety may be high but still remains in an energetically feasible range. The data point for **5fC** and **5fU** are evidently outliers for the dataset of **5hm**, while **5hm6aU**, **5hmU** and **5hmC** are more or less colinear. If we were to include **5fC** and **5fU** in the linear regression, the R^2 would drop to 0.677, suggesting a less satisfactory linearity. These two outliers are, however, perfectly on the line with their hydrated counterparts **5dhmC** and **5dhmU**, excluding **5f6aU** and **5dhm6aU**. This suggests that the oxidation of the aza-substituted **5f6aU** undergoes a different mechanism. This brings us to our conclusion, the oxidation actually goes through hydrate for the species which build a lot of hydrates, combined with the fact that the BDE for the CH of the geminal diol is much lower than the original formyl group, thereby facilitating oxidation.

S6. Appendix

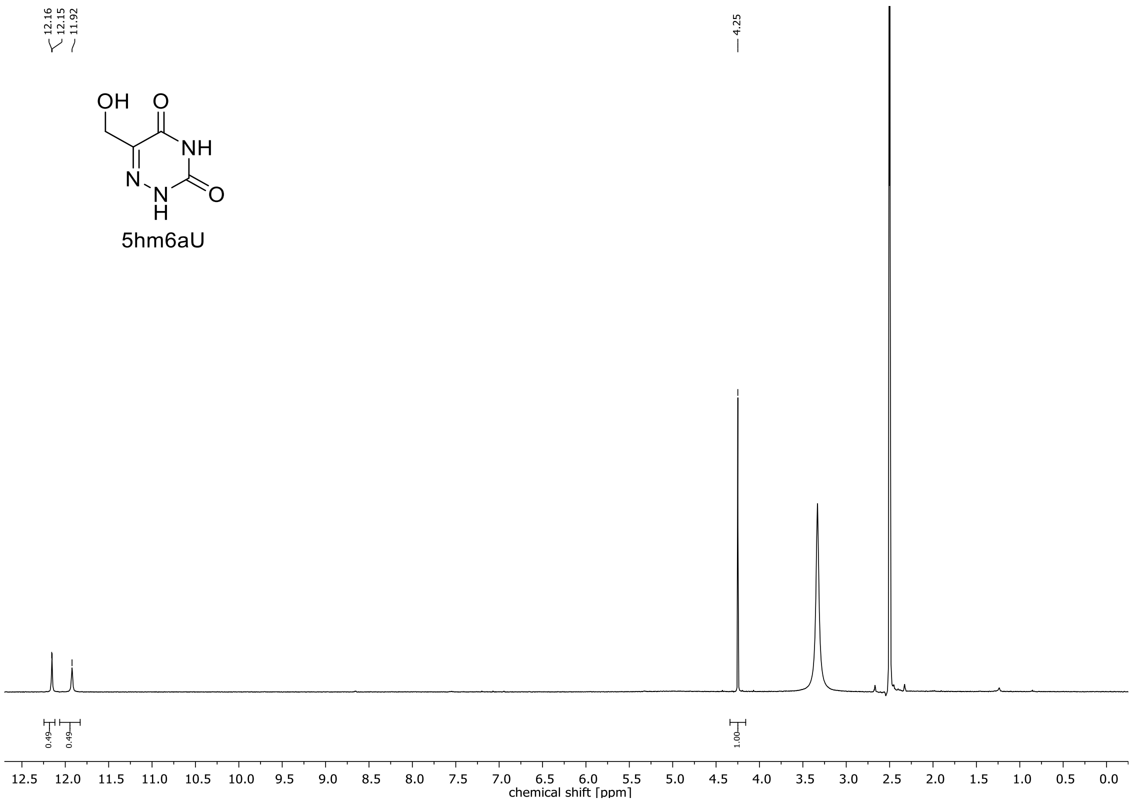

Figure S31. ^{1}H-NMR spectrum of 5hm6aU (DMSO-d_6, 400 MHz, 295 K).

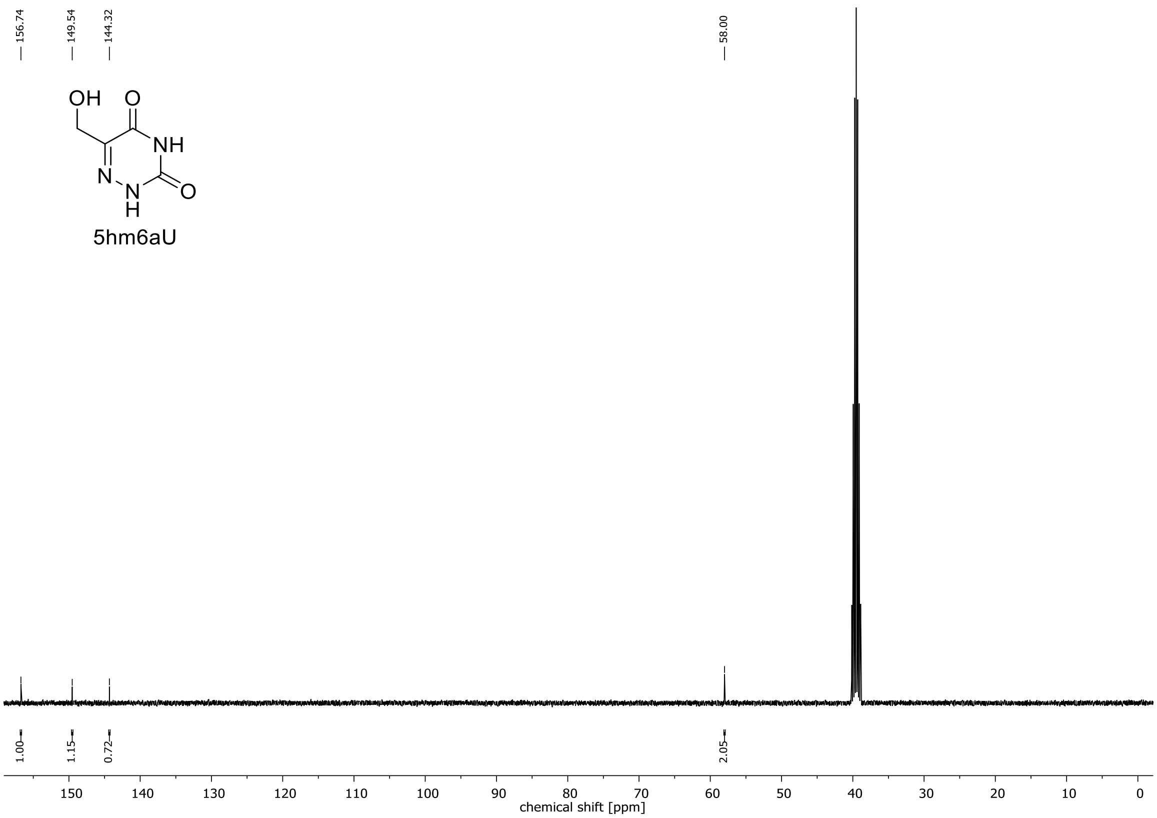

Figure S32. ^{13}C-NMR spectrum of 5hm6aU (DMSO-d_6, 101 MHz, 295 K).

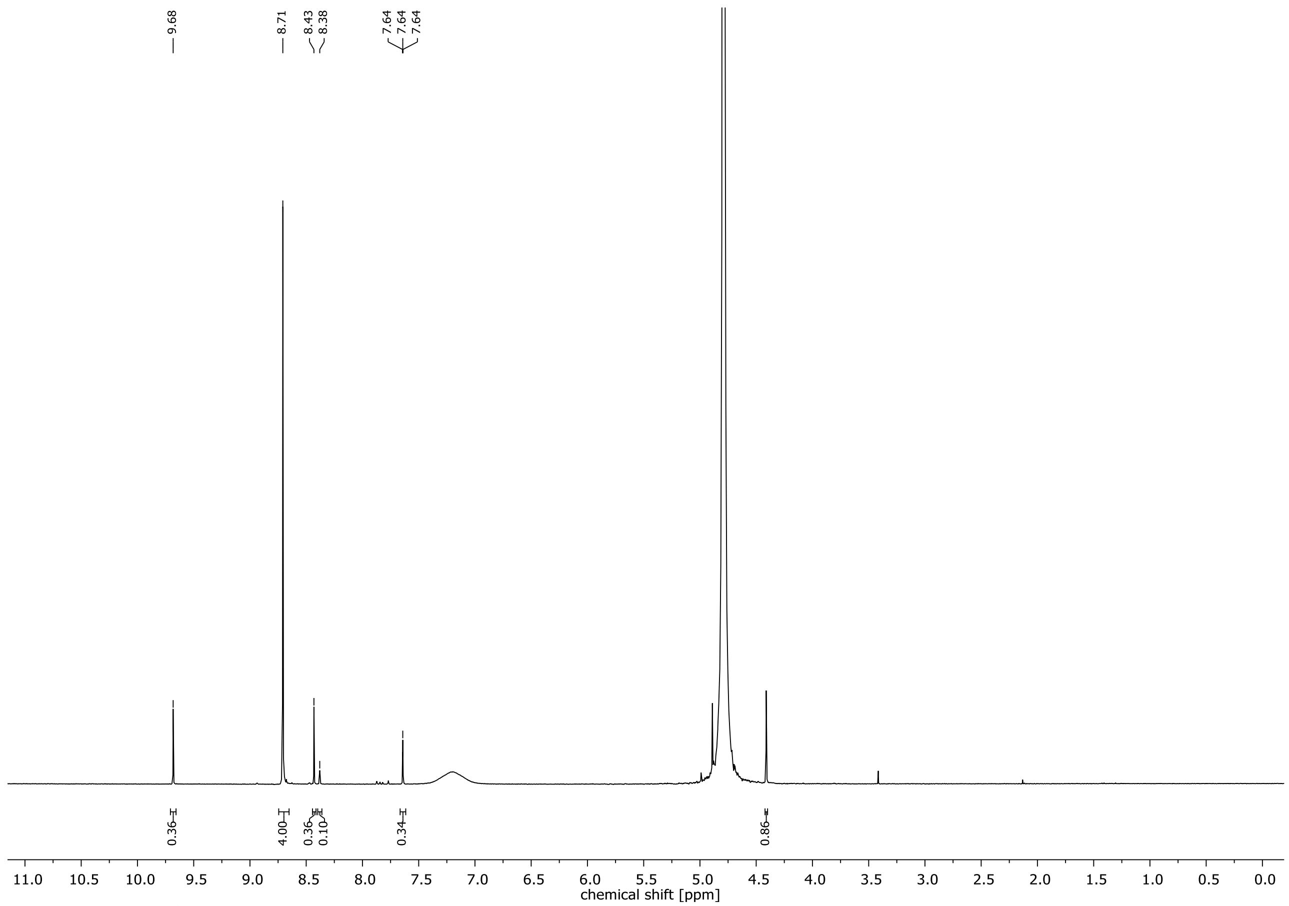

Figure S33. ^{1}H NMR spectrum of the reaction mixture of 5hmU with **TM1** after a reaction time of 20 min (D_2O, 400 MHz, 295 K).

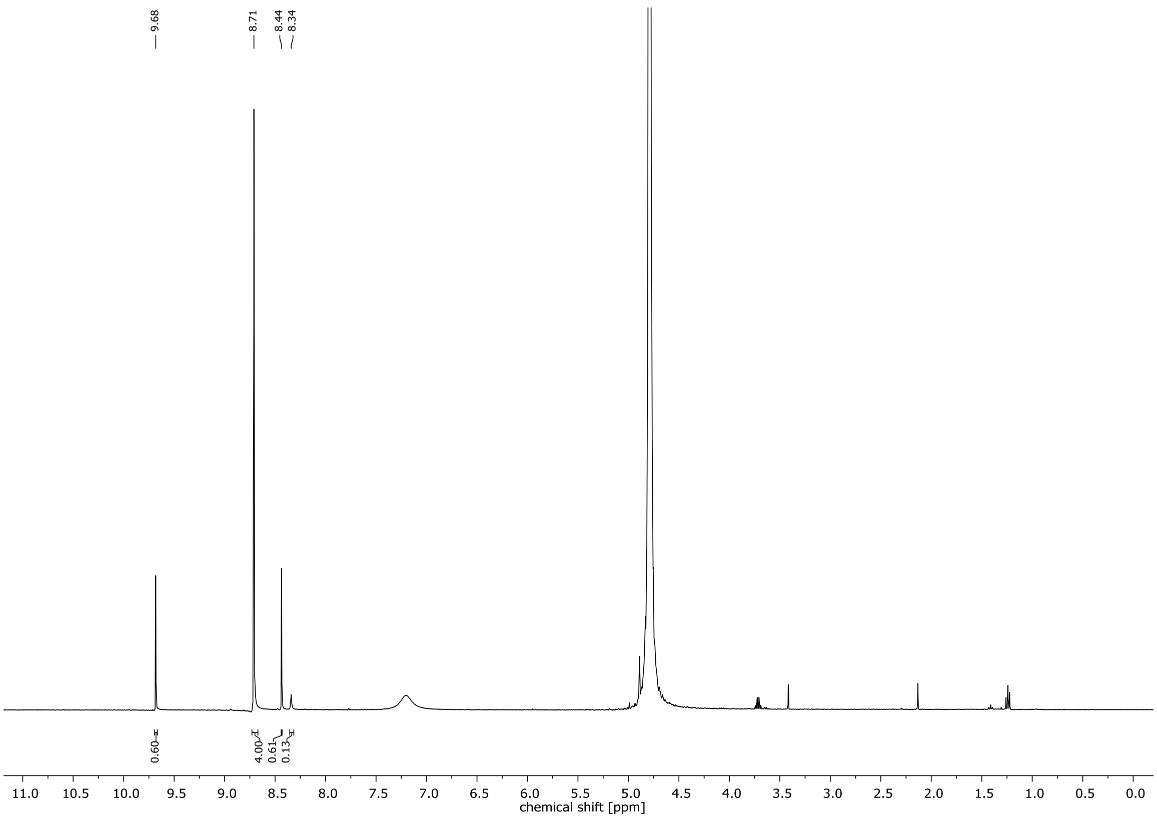

Figure S34. ^{1}H NMR spectrum of the reaction mixture of 5fU with **TM1** after a reaction time of 20 min (D_2O, 400 MHz, 295 K).

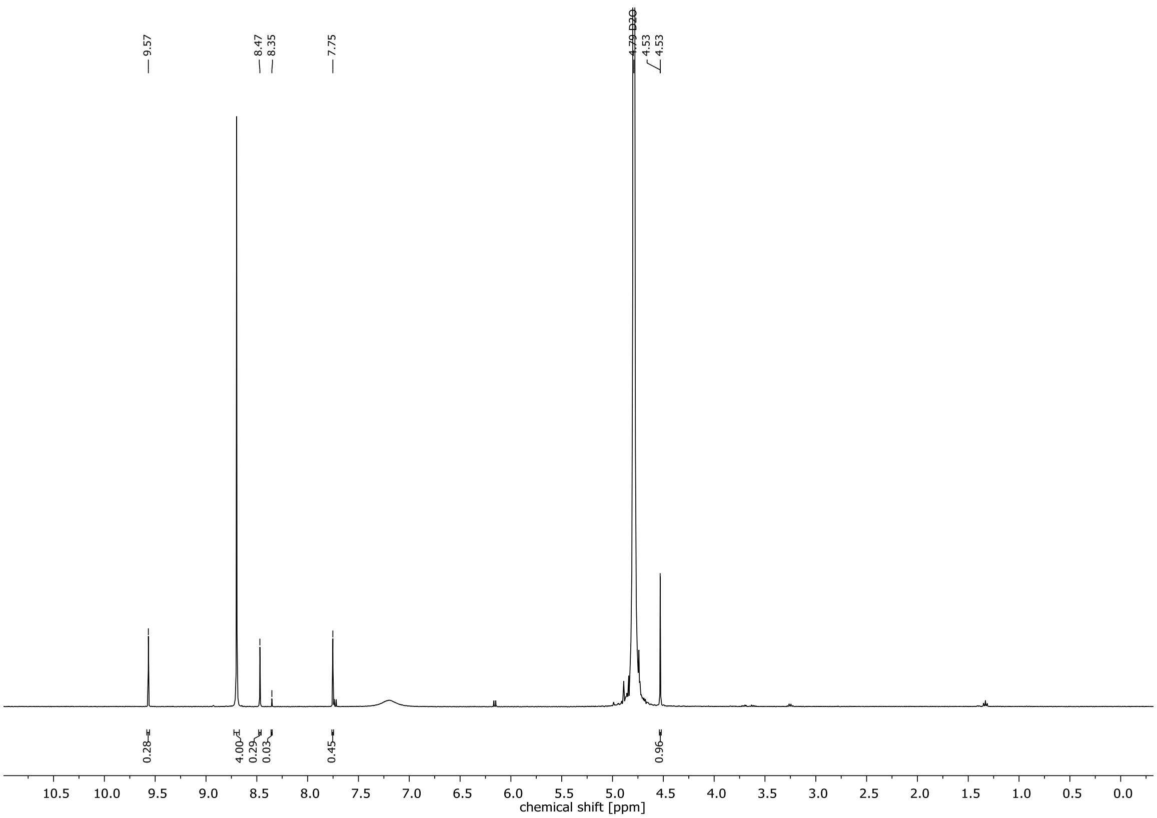

Figure S35. ^{1}H NMR spectrum of the reaction mixture of 5hmC with **TM1** after a reaction time of 20 min (D_2O, 400 MHz, 295 K). See section S3 for experimental setup.

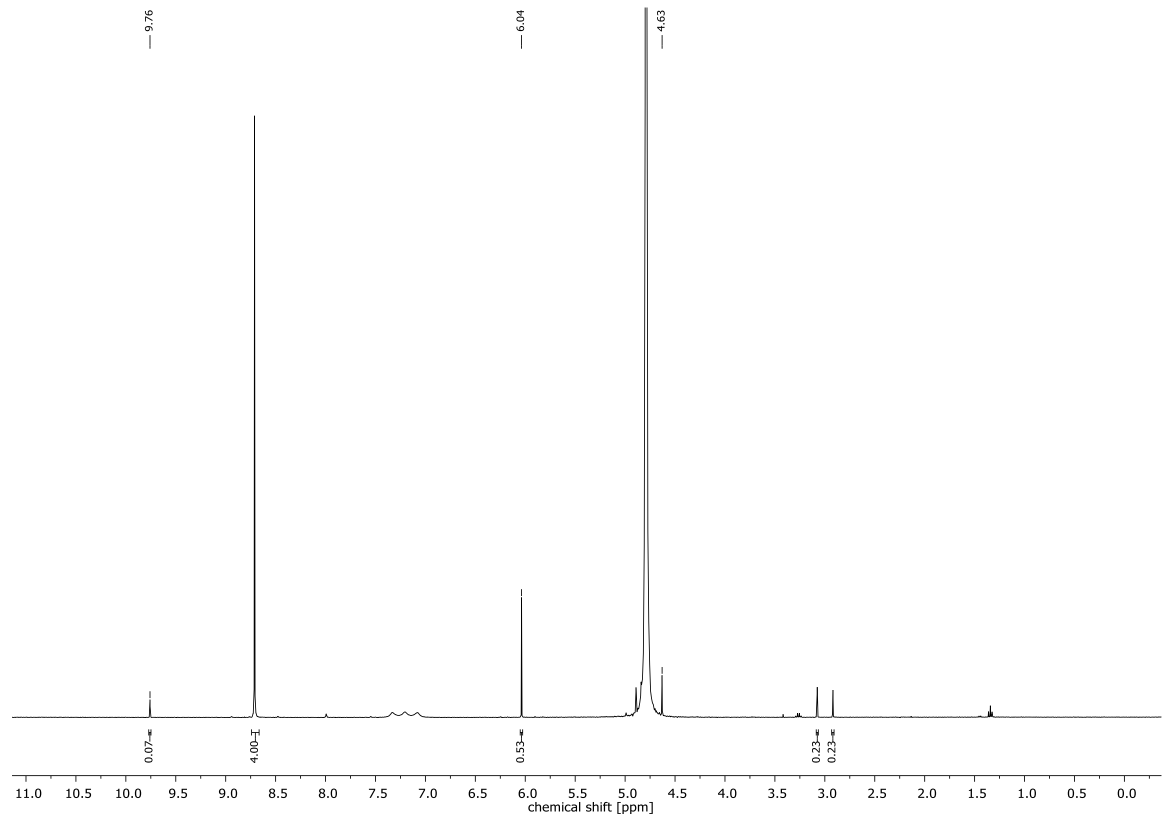

Figure S36. ^{1}H NMR spectrum of the reaction mixture of 5hm6aU with **TM1** after a reaction time of 20 min (D_2O, 400 MHz, 295 K).

S7. Literature

[1] a) T. Chantarojsiri, Y. Sun, J. R. Long, C. J. Chang, *Inorganic Chemistry* **2015**, *54*, 5879-5887; b) N. S. W. Lindlar né Jonasson, A. Menke, L. Senft, A. Squarcina, D. Schmidl, K. Fisher, S. Demeshko, J. C. Kruse, T. Josephy, P. Mayer, J. Gutenthaler-Tietze, P. Comba, F. Meyer, I. Ivanović-Burmazović, L. J. Daumann, *Inorganic Chemistry* **2025**, *64*, 3719-3734.

[2] F. L. Zott, V. Korotenko, H. Zipse, *ChemBioChem* **2022**, *23*, e202100651.

[3] A. Schön, E. Kaminska, F. Schelter, E. Ponkkonen, E. Korytiaková, S. Schiffers, T. Carell, *Angewandte Chemie International Edition* **2020**, *59*, 5591-5594.

[4] N. S. W. Jonasson, R. Janßen, A. Menke, F. L. Zott, H. Zipse, L. J. Daumann, *ChemBioChem* **2021**, *22*, 3333-3340.

Supporting Information II

Aldehyde or Hydrate? Investigation into the Oxidation of 5-Formylcytosine Derivatives using a Computational and Experimental Approach

Kuangjie Liu§, Annika Menke§, Fabian L. Zott§, Domenic Mayer, Lena J. Daumann*, Hendrik Zipse*

lena.daumann@hhu.de

Faculty of Mathematics and Natural Sciences, Heinrich-Heine-Universität Düsseldorf, Universitätsstr. 1, 40225 Düsseldorf, Germany

zipse@cup.uni-muenchen.de

Faculty of Chemistry and Pharmacy, Ludwig-Maximilians University Munich, Butenandtstr. 5-13, 81377 Munich, Germany

BOND DISSOCIATION ENERGY CALCULATIONS

Methodology

As in previous studies on radical stabilities,[1-7] geometry optimizations have been performed with a combination of the (U)B3LYP hybrid functional[8] complemented by the D3 dispersion correction[10] and the 6-31+G(d,p) basis set[11-12] in the gas phase. Thermochemical corrections (corr. ΔH & ΔG) to 298.15 K have been calculated at the same level of theory using the rigid rotor/harmonic oscillator model. Enthalpies (ΔH_{298}) and Gibbs energies (ΔG_{298}) at (U)B3LYP-D3/6-31+G(d,p) level have been obtained through addition of corr. ΔG and corr. ΔH to ΔE_{tot}, respectively. Single point energies have subsequently been calculated with the DLPNO-CCSD(T) method[13-14] as implemented in ORCA 4.2.1[15] in combination with the cc-pVTZ and cc-pVQZ basis sets, followed by extrapolation to the complete basis set (CBS) limit to DLPNO-CCSD(T)/CBS total energies.[16-18] Enthalpies at 298.15 K have subsequently been calculated through combination of the DLPNO-CCSD(T)/CBS total energies with thermochemical corrections (ΔH) calculated at the (U)B3LYP-D3/6-31+G(d,p) level before. In order to estimate the impact of aqueous solvation, single point energies have been calculated at the SMD(H_2O)/(U)B3LYP-D3/6-31+G(d,p) level using the gas phase-optimized geometries.[19] Solvation free energies in water (ΔG_{solv}) have then been obtained as the difference of total energies obtained from SMD(H_2O)/(U)B3LYP-D3/6-31+G(d,p) and (U)B3LYP-D3/6-31+G(d,p) calculations. Combination of the solvation energies with gas phase enthalpies then yield enthalpy values in water. Only conformers with an E_{tot} difference of 70 kJ/mol relative to the best conformer were taken into consideration or above 2% Boltzmann population at the (U)B3LYP-D3/6-31+G(d,p)/SMD(H_2O)//DLPNO-CCSD(T)/CBS level of theory.

Radical stabilization energies (RSE) for C-centered radicals generated by C-H homolytic bond cleavage are measured with reference to toluene ($PhCH_2$-H) using the isodesmic hydrogen exchange reaction shown in eq. S1. R-CH_2H bond dissociation energies (BDE) can then be derived from the calculated RSE values through the addition of the reference BDE(C-H) value in $PhCH_2$-H (375.5 ± 5 kJ/mol^{-1})[20] as expressed in eq. S2. In the subsequent discussion we assume this value to be valid in the gas phase as well as in aqueous solution.

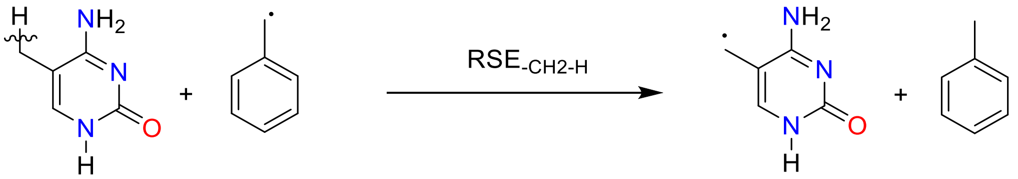

Eq. S1

$$BDE_{RCH_2-H} = RSE_{RCH_2-H} + BDE^{Exp}_{RCH_2-H} \quad \textbf{Eq. S2}$$

In **Figure S1** the modified nucleobases under investigation are depicted. The number indicates the position of the modification. The prefix "n_" indicates a neutral molecule. For radicals, "r_" precedes the modification-location marker.

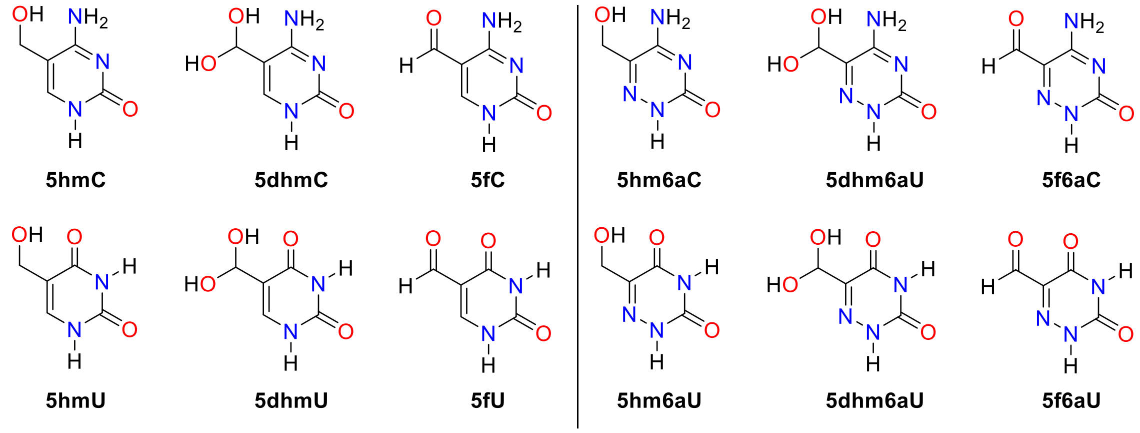

Figure S1: Naming of modified nucleobases under investigation (n_ = neutral, r_ = radical). The letter "a" preceding C (cytosine) and U (uracil) indicates the 6-aza derivatives (see right side).

Calculations of BDE and RSE values:

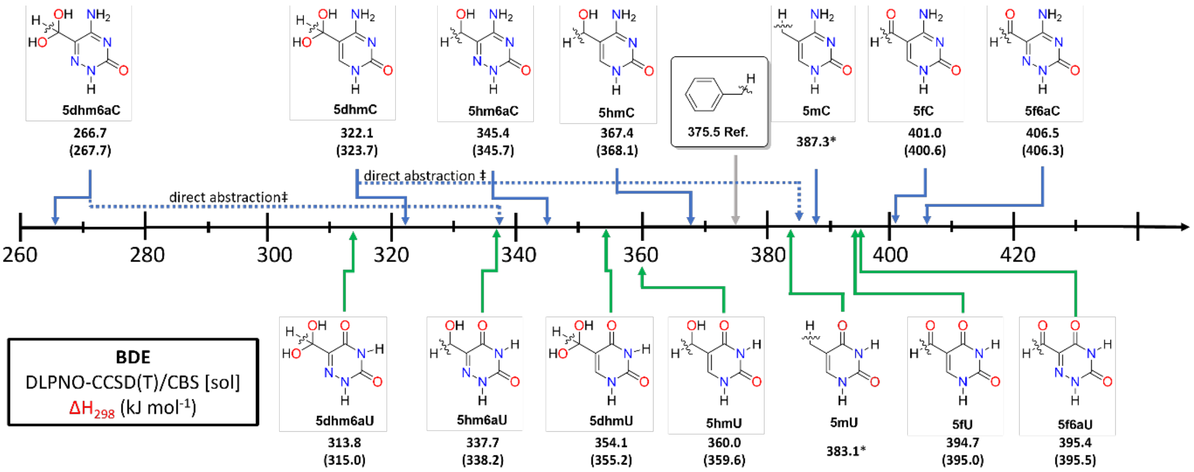

Figure S2: Boltzmann averaged aqueous phase ($\Delta H_{sol} = \Delta H_{298} + \Delta G_{solv}$) RCH_2-H bond dissociation energies (BDEs) calculated at the DLPNO-CCSD(T)/CBS level of theory (BDE for best conformers in brackets, see Table S1). The BDE values of thymine (**5mU**) and 5-methylcytosine (**5hmC**) marked with an asterisk are taken from earlier studies.[9] (‡ direct abstraction: BDE(C-H) without tautomeric proton shift, explained in Figure S3)

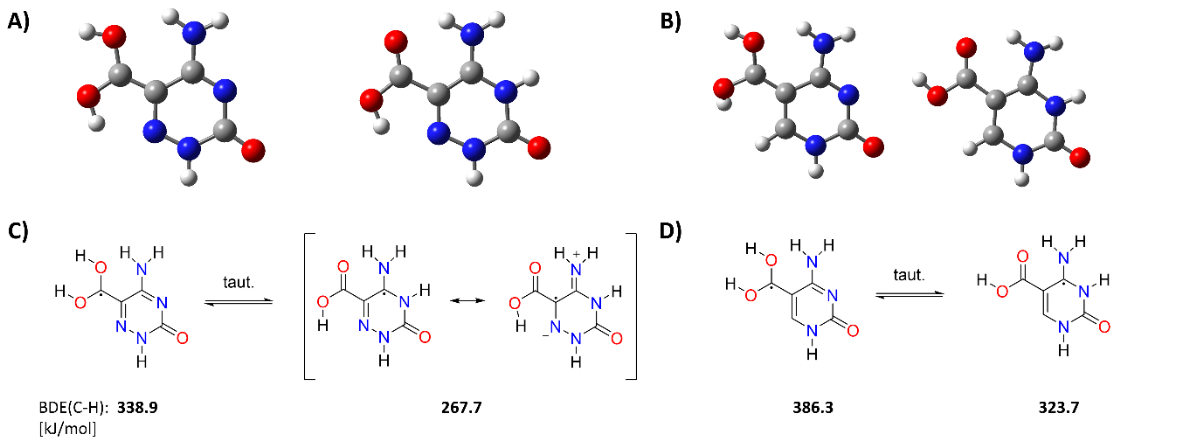

Figure S3: Rationalization of radical stabilization for tautomeric forms: A) and B) lowest energy conformer for both tautomeric radicals of **5dhm6aC** and **5dhmC**; C) Tautomerization and captodative effect of **5dhm6aC**; D) Tautomerization of **5dhmC** (Displayed with aqueous phase BDE(C-H) energies for the best conformer at the DLPNO-CCSD(T)/CBS level of theory; compare with value in brackets, **Figure S2**).

In **Figure S1** and **Table S1**, the RSE and BDE values of modified cytosine and uracil nucleobases in aqueous phase with toluene as a reference compound (with BDE(C-H) = +375.5 kJ mol^{-1}) can be seen. The BDE(C-H) values marked with an asterisk for 5mC and 5mU are literature values from our previous investigation.[9] The highest C-H bond energies can be found for the 5-formyl derivatives of cytosine and uracil (**5fC**, **5f6aC**, **5fU** and **5f6aU**), where the 6-aza derivatives have slightly higher BDE value of up to +5.5 kJ mol^{-1} relative to their non-aza counterparts. In case of **5hmC** and **5hmU** the N substitution at the 6-position decreases the BDE value for both compounds by 22 kJ mol^{-1}. The effects of 6-aza substitution are even larger for the 5-dihydroxymethyl systems. The BDE decreases from **5dhmU** to **5dhm6aU** by 40.3 kJ mol^{-1}, and from **5dhmC** to **5dhm6aC** by 55.4 kJ mol^{-1}. These substituent effects can be rationalized by donor/acceptor interactions between the amino group as donor and the nitrogen atom at the 6-position as acceptor The resulting captodative effect is expressed by the Lewis structures (**Figure S3C**), and not possible for the **5dhmC**, **5dhm6aC** and **5dhm6aU** analogues (**Figure S3D**). It should also be mentioned that the lowest energy conformer at the DLPNO-CCSD(T)/CBS level of theory depicted in **Figure S3A** and **Figure S3B**. They show an additional intramolecular hydrogen bond in **5dhm6aC** between the 6-aza position and the carboxy group, further adding a stabilizing effect versus the **5dhmC** derivative. When determining the direct C-H bond abstraction from the lowest energy conformer of **5dhm6aC** while neglecting a tautomeric proton shift, the BDE (C-H) value is 338.9 kJ mol^{-1}(see **Figure 3A** and **Figure 3C**) and 386.3 kJ mol^{-1} for **5dhmC** (see **Figure S3B** and **S3D**). This means that the tautomerization contributes 62.6 kJ mol^{-1} for the **5dhmC**, while a combination of tautomerization and captodative stabilization contributes 71.2 kJ mol^{-1} for **5dhm6aC**.

The values obtained here for **5hmC** and **5fC** are largely similar to those reported by Hu et al. at several different levels in the gas phase (BDE(**5hmC**,CBS-QB3) = +366.1 kJ/mol and BDE(**5fC**, CBS-QB3 = +384.8 kJ/mol) as well as in aqueous solution (BDE(**5hmC**,CPCM/CBS-QB3) = +360.7 kJ/mol and BDE(**5fC**,CPCM/CBS-QB3) = +388.7 kJ/mol).[21] While the gas phase values are very similar, solution phase calculations exhibit differences due to an absolute bond cleavage reaction (R–H→R•+H•) being used instead of a relative method with toluene as a reference.

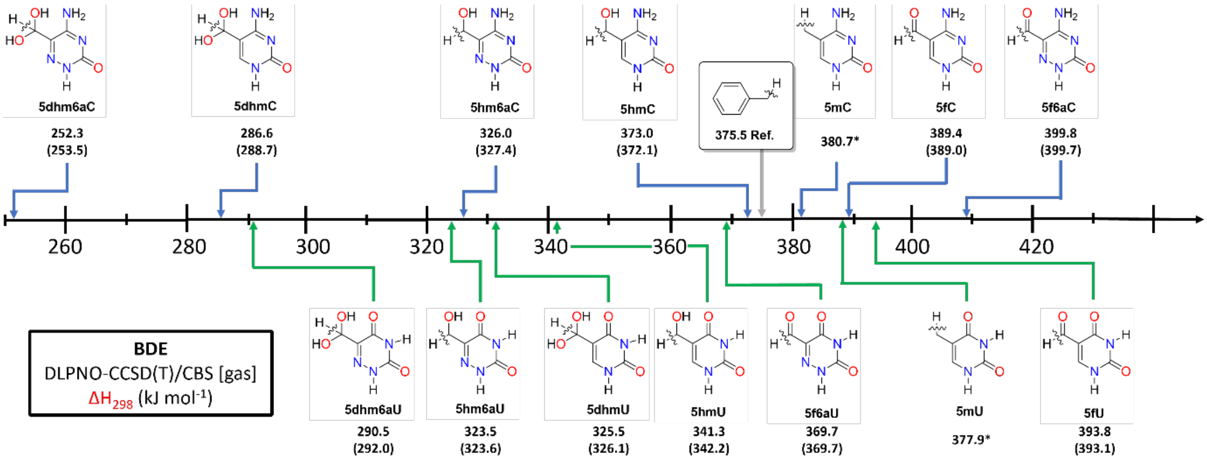

Figure S4: Boltzmann averaged gas phase (ΔH_{gas}) RCH_2-H bond dissociation energies (BDEs) calculated at the DLPNO-CCSD(T)/CBS level of theory (BDE for best conformers in brackets, see **Table S2**). The BDE value of thymine (**5mU**) and 5-methylcytosine (**5mC**) marked with an asterisk are taken from earlier studies.[9]

Table S1: Boltzmann-averaged, gas- and solution phase RSE und BDE values with toluene as reference for the systems shown in **Figure S1**.

compound	gas phase				water (SMD model)				$\Delta\Delta G_{solv}$ [kJ/mol]
	(U)B3LYP-D3/ 6-31+G(d,p)[a]		DLPNO-CCSD(T)/ CBS[a]		(U)B3LYP-D3/ 6-31+G(d,p)[a]		DLPNO-CCSD(T)/ CBS[a]		
	RSE - ΔH_{298} [kJ/mol]	BDE - ΔH_{298} [kJ/mol]	RSE - ΔH_{298} [kJ/mol]	BDE - ΔH_{298} [kJ/mol]	RSE - ΔH_{298} [kJ/mol]	BDE - ΔH_{298} [kJ/mol]	RSE - ΔH_{298} [kJ/mol]	BDE - ΔH_{298} [kJ/mol]	
**toluene**		**375.5**[b]							
**5hmC**	-12.47	363.03	-2.50	373.00	-13.92	361.58	-8.09	**367.41**	-5.6
**5hm6aC**	-59.68	315.82	-49.50	326.00	-35.52	339.98	-30.08	**345.42**	19.4
**5fC**	10.94	386.44	13.86	389.36	23.96	399.46	25.49	**400.99**	11.6
**5f6aC**	23.26	399.79	24.29	399.79	29.95	405.45	31.00	**406.50**	6.7
**5dhmC**	-103.67	271.83	-88.91	286.59	-67.01	308.49	-53.45	**322.05**	35.5
**5dhm6aC**	-135.46	240.04	-123.24	252.26	-119.62	255.88	-108.79	**266.71**	14.5
**5hmU**	-46.56	328.94	-34.24	341.26	-26.82	348.68	-15.55	**359.95**	18.7
**5hm6aU**	-67.01	308.49	-52.05	323.45	-52.16	323.34	-37.85	**337.65**	14.2
**5fU**	19.89	395.39	18.31	393.81	18.22	393.72	19.16	**394.66**	0.9
**5f6aU**	-9.78	365.72	-5.79	369.71	14.08	389.58	19.88	**395.38**	25.7
**5dhmU**	-65.38	310.12	-50.04	325.46	-36.22	339.28	-21.36	**354.14**	28.7
**5dhm6aU**	-99.52	275.98	-85.03	290.47	-76.12	299.38	-61.75	**313.75**	23.3

[a]: Using gas phase optimized (U)B3LYP-D3/6-31+G(d,p) geometries. [b] Reference value: BDE(Ph-CH_2-H) = 375.5 ± 5 kJ/mol.[22]

Table S2: Best conformer, gas- and solution phase RSE und BDE values with toluene as reference for the systems shown in **Figure S1**.

compound	gas phase				water (SMD model)				$\Delta\Delta G_{solv}$ [kJ/mol]
	(U)B3LYP-D3/ 6-31+G(d,p)[a]		DLPNO-CCSD(T)/ CBS[a]		(U)B3LYP-D3/ 6-31+G(d,p)[a]		DLPNO-CCSD(T)/ CBS[a]		
	RSE - ΔH_{298} [kJ/mol]	BDE - ΔH_{298} [kJ/mol]	RSE - ΔH_{298} [kJ/mol]	BDE - ΔH_{298} [kJ/mol]	RSE - ΔH_{298} [kJ/mol]	BDE - ΔH_{298} [kJ/mol]	RSE - ΔH_{298} [kJ/mol]	BDE - ΔH_{298} [kJ/mol]	
**toluene**		**375.5[b]**							
**5hmC**	-11.71	363.79	-3.42	372.08	-13.65	361.85	-7.42	**368.08**	-4.0
**5hm6aC**	-58.96	316.54	-48.14	327.36	-35.56	339.94	-29.82	**345.68**	18.3
**5fC**	10.73	386.23	13.52	389.02	23.80	399.30	25.12	**400.62**	11.6
**5f6aC**	23.23	398.73	24.23	399.73	29.78	405.28	30.78	**406.28**	6.5
**5dhmC**	-101.52	273.98	-86.77	288.73	-65.52	309.98	-51.80	**323.70**	35.0
**5dhm6aC**	-134.22	241.28	-121.99	253.51	-118.58	256.92	-107.80	**267.70**	14.2
**5dhm6aC[c]**					-49.08	326.42	-36.64	**338.86**	
**5hmU**	-45.64	329.86	-33.31	342.19	-25.99	349.51	-15.95	**359.55**	17.4
**5hm6aU**	-66.77	308.73	-51.92	323.58	-52.18	323.32	-37.33	**338.17**	14.6
**5fU**	19.30	394.80	17.64	393.14	17.95	393.45	19.46	**394.96**	1.8
**5f6aU**	-9.17	366.33	-5.75	369.75	14.54	390.04	19.95	**395.45**	25.7
**5dhmU**	-64.72	310.78	-49.36	326.14	-35.05	340.45	-20.27	**355.23**	29.1
**5dhm6aU**	-98.44	277.06	-83.54	291.96	-74.54	300.96	-60.48	**315.02**	23.1

[a]: Using gas phase optimized (U)B3LYP-D3/6-31+G(d,p) geometries. [b] Reference value: BDE($Ph-CH_2-H$) = 375.5 ± 5 kJ/mol.[22]
[c]: BDE value for direct abstraction without tautomeric shift of a proton (see **Figure S3**).

Thermodynamic data

Table S3: Enthalpies for gas phase optimized conformers shown in **Table S4** and **Table S5** calculated at the (U)B3LYP-D3\6-31G+(d,p) level of theory (r = radical).

compound	(U)B3LYP-D3/ 6-31+G(d,p)[a,b]	DLPNO-CCSD(T)/ TZ[a,b]	DLPNO-CCSD(T)/ QZ[a,b]	DLPNO-CCSD(T)/ CBS[a,b]
	Boltzmann averaged, aqueous **H_{298} [kJ/mol]**			
tol	-271.4630588	-270.9120373	-270.9894210	-271.0377006
rtol	-270.8245230	-270.2723251	-270.3484469	-270.3958873
5hmC	-509.4107345	-508.5560674	-508.7114408	-508.8072000
r5hmC	-508.7774988	-507.9191895	-508.0735558	-508.1684692
5fC	-508.2277346	-507.3751059	-507.5279899	-507.6220786
r5fC	-507.5800722	-506.7266448	-506.8776901	-506.9705579
5dhmC	-584.6430881	-583.6931206	-583.8729764	-583.9837533
r5dhmC	-584.0300525	-583.0738694	-583.2522588	-583.3622276
5hm6aC	-525.4323570	-524.5691149	-524.7293213	-524.8280843
r5hm6aC	-524.8073510	-523.9404442	-524.0998222	-524.1977290
5f6aC	-524.2406315	-523.3801772	-523.5377500	-523.6347222
r5f6aC	-523.5906891	-522.7293934	-522.8852921	-522.9811002
5dhm6aC	-600.6622745	-599.7035061	-599.8879725	-600.0016038
r5dhm6aC	-600.0692845	-599.1048097	-599.2882765	-599.4011750
5hmU	-529.2986276	-528.4365919	-528.5981369	-528.6976625
r5hmU	-528.6703076	-527.8034237	-527.9632746	-528.0617733
5fU	-528.1084077	-527.2507367	-527.4090749	-527.5065689
r5fU	-527.4629329	-526.6045434	-526.7611378	-526.8574564
5dhmU	-604.5319138	-603.5739630	-603.7596116	-603.8739829
r5dhmU	-603.9071732	-602.9430047	-603.1269595	-603.2403046
5hm6aU	-545.3164530	-544.4460957	-544.6123539	-544.7147550
r5hm6aU	-544.6579090	-543.7826890	-543.9471986	-544.0484393
5f6aU	-544.1173340	-543.2523753	-543.4154478	-543.5158117
r5f6aU	-543.4734372	-542.6052090	-542.7669887	-542.8664253
5dhm6aU	-620.5482022	-619.5821781	-619.7724955	-619.8897082
r5dhm6aU	-619.9383238	-618.9663419	-619.1550228	-619.2711602
	best conformer, aqueous **H_{298} [kJ/mol]**			
tol	-271.463059	-270.912037	-270.989421	-271.037701
rtol	-270.824523	-270.272325	-270.348447	-270.395887
5hmC	-509.411238	-508.556665	-508.712051	-508.807818
r5hmC	-508.777901	-507.919589	-508.073928	-508.168831
5fC	-508.227898	-507.375278	-507.528132	-507.622212
r5fC	-507.580299	-506.726955	-506.877970	-506.970830
5dhmC	-584.643754	-583.693845	-583.873803	-583.984585
r5dhmC	-584.030173	-583.074183	-583.252554	-583.362500
5dhmC_dir[c]	-584.006774	-583.050434	-583.228918	-583.338669
5hm6aC	-525.4327428	-524.5695525	-524.7297037	-524.8283819
r5hm6aC	-524.8077519	-523.9407774	-524.1000439	-524.1979248
5f6aC	-524.240634	-523.380182	-523.537755	-523.634726
r5f6aC	-523.590757	-522.729500	-522.885390	-522.981190
5dhm6aC	-600.662701	-599.703737	-599.888239	-600.001932
r5dhm6aC	-600.069330	-599.104815	-599.288281	-599.401179
5dhm6aC_dir[c]	-600.042858	-599.077593	-599.261188	-599.374075
5hmU	-529.299091	-528.436864	-528.598382	-528.697904
r5hmU	-528.670453	-527.803614	-527.963627	-528.062164
5fU	-528.108493	-527.250998	-527.409336	-527.506832
r5fU	-527.463119	-526.604691	-526.761284	-526.857607
5dhmU	-604.532359	-603.574361	-603.760019	-603.874398
r5dhmU	-603.907173	-602.943005	-603.126960	-603.240305
5hm6aU	-545.3167119	-544.4463576	-544.6125961	-544.7149773
r5hm6aU	-544.6579836	-543.7829212	-543.9474361	-544.0486735
5f6aU	-544.117596	-543.252587	-543.415666	-543.516034
r5f6aU	-543.473523	-542.605464	-542.767213	-542.866621
5dhm6aU	-620.548695	-619.582640	-619.772972	-619.890189
r5dhm6aU	-619.938548	-618.966597	-619.155276	-619.271413

[a] Using gas phase optimized (U)B3LYP-D3/6-31+G(d,p) geometries. [b] Without standard state correction of $\Delta G_{0K\rightarrow 298K}^{1atm\rightarrow 1M}$= +7.91 kJ mol^{-1}. [c] lowest energy conformer that can facilitate the direct C-H abstraction at the 5-position without tautomeric proton shift (see **Figure S3**).

Table S4: Energies for gas phase optimized conformers and tautomers for modified cytosine bases shown in Fig. 1 calculated at the (U)B3LYP-D3\6-31+G(d,p) level of theory.

compound	E_{tot}[a] (UB3LYP-D3/ 6-31+G(d,p))	E_{tot}[a] (SMD(H2O)/ UB3LYP-D3/ 6-31+G(d,p))	E_{tot}[a] (DLPNO-CCSD(T)/ cc-pVTZ)	E_{tot}[a] (DLPNO-CCSD(T)/ cc-pVQZ)	E_{tot}[a] (DLPNO-CCSD(T)/ CBS)	corr. ΔH[a] (UB3LYP-D3/ 6-31+G(d,p))	corr. ΔG[a,b] (UB3LYP-D3/ 6-31+G(d,p))
	**[Hartree]**					**[Hartree]**	
**toluene**							
tol	-271.597084	-271.597992	-271.046063	-271.123446	-271.171726	0.134933	0.097272
r_tol	-270.945089	-270.945771	-270.392891	-270.469013	-270.516453	0.121248	0.084945
**5hmC**							
5hmC_C5	-509.515899	-509.553322	-508.661326	-508.816712	-508.912479	0.142084	0.098521
5hmC_C9	-509.513863	-509.551849	-508.659516	-508.815027	-508.910819	0.142010	0.098416
5hmC_C7	-509.509991	-509.550141	-508.656050	-508.811638	-508.907449	0.141889	0.097689
5hmC_C3	-509.508924	-509.549834	-508.655291	-508.810730	-508.906532	0.141763	0.097140
5hmC_C1	-509.509002	-509.549182	-508.654879	-508.810784	-508.906806	0.141589	0.096635
5hmC_C6	-509.506259	-509.546999	-508.650863	-508.805985	-508.901616	0.141696	0.097926
5hmC_C4	-509.506259	-509.546999	-508.650863	-508.805985	-508.901616	0.141696	0.097926
5hmC_C8	-509.506259	-509.546999	-508.650863	-508.805985	-508.901616	0.141696	0.097926
5hmC_C14	-509.515348	-509.542802	-508.664701	-508.819501	-508.914994	0.142123	0.098939
5hmC_C16	-509.515348	-509.542802	-508.664701	-508.819501	-508.914994	0.142123	0.098939
r5hmC_C3_2	-508.867590	-508.905982	-508.009278	-508.163617	-508.258520	0.128081	0.083035
r5hmC_C3_1	-508.864775	-508.903750	-508.007249	-508.161395	-508.256180	0.127977	0.083074
r5hmC_C9_1	-508.867894	-508.904721	-508.008156	-508.162277	-508.257134	0.127930	0.082597
r5hmC_C17_1	-508.863533	-508.895494	-508.009357	-508.163122	-508.257753	0.127947	0.083291
r5hmC_C20_2	-508.865068	-508.895681	-508.010721	-508.164522	-508.259176	0.128030	0.083423
**5fC**							
5fC_C1	-508.312605	-508.345610	-507.459985	-507.612839	-507.706919	0.117712	0.075422
5fC_t2C2	-508.307934	-508.342692	-507.455226	-507.607823	-507.701816	0.117379	0.075010
5fC_C2	-508.302753	-508.338574	-507.450212	-507.602975	-507.697040	0.117335	0.072815
5fC_t4C2	-508.311412	-508.335415	-507.462823	-507.615110	-507.708956	0.117772	0.075884
5fC_t4C1	-508.312354	-508.335598	-507.463468	-507.615822	-507.709702	0.117826	0.075978
5fC_t2C1	-508.292428	-508.335651	-507.440698	-507.593189	-507.687099	0.115964	0.072978
5fC_t3C1	-508.302996	-508.332709	-507.454014	-507.606287	-507.700094	0.118100	0.075647
5fC_t4C3	-508.298488	-508.328231	-507.450860	-507.602964	-507.696709	0.117238	0.072473
5fC_t4C4	-508.300127	-508.328333	-507.452143	-507.604336	-507.698136	0.117339	0.073295
5fC_t3C4	-508.288409	-508.326500	-507.441580	-507.593610	-507.687255	0.117484	0.074184
r5fC_C1	-507.657983	-507.685787	-506.804639	-506.955654	-507.048514	0.105488	0.062453
r5fC_t2C2	-507.650383	-507.682266	-506.797487	-506.948379	-507.041233	0.105107	0.061878
r5fC_C2	-507.653083	-507.682104	-506.799923	-506.950880	-507.043718	0.105147	0.061396
r5fC_t2C1	-507.646546	-507.680521	-506.793691	-506.944397	-507.037114	0.104893	0.060040
r5fC_t4C2	-507.655196	-507.675584	-506.806307	-506.956883	-507.049591	0.105482	0.062810
**5dhmC**							
5dhmC1a1	-584.741505	-584.790730	-583.791596	-583.971554	-584.082336	0.146976	0.098972
5dhmC1b6	-584.744477	-584.790325	-583.794915	-583.974347	-584.084943	0.147504	0.101109
5dhmC1b5	-584.750618	-584.791132	-583.800263	-583.979823	-584.090502	0.147756	0.101950
5dhmC1b3	-584.748412	-584.790255	-583.798465	-583.977966	-584.088593	0.147807	0.102081
5dhmC1b1	-584.744315	-584.788951	-583.794709	-583.974409	-584.085100	0.147411	0.100746
5dhmC1a6	-584.744807	-584.789052	-583.794454	-583.974326	-584.085101	0.147068	0.099435
5dhmC1a2	-584.749065	-584.790029	-583.799084	-583.978474	-584.089000	0.147857	0.102201
5dhmC1a8	-584.744149	-584.788107	-583.794644	-583.974031	-584.084543	0.147548	0.101721
5dhmC2a1	-584.741209	-584.779931	-583.795198	-583.974586	-584.085099	0.147094	0.099618
5dhmC4b4	-584.738920	-584.784614	-583.788204	-583.967516	-584.078047	0.147351	0.101318
r5dhmC_a17	-584.137530	-584.164482	-583.181539	-583.359911	-583.469856	0.134309	0.086941
r5dhmC_a3	-584.127737	-584.160196	-583.173754	-583.352057	-583.461840	0.134319	0.087561
r5dhmC_a16	-584.132556	-584.160898	-583.177492	-583.355703	-583.465521	0.134325	0.086895
r5dhmC_a13	-584.115119	-584.143519	-583.160937	-583.339116	-583.448822	0.133630	0.087201
r5dhmC_a5	-584.106361	-584.138890	-583.154544	-583.332792	-583.442435	0.133447	0.086877
**5hm6aC**							
5hm6aC_t1C4	-525.528072	-525.562492	-524.664882	-524.825033	-524.923711	0.129749	0.086249
5hm6aC_t1C3	-525.521835	-525.559427	-524.659466	-524.820393	-524.919402	0.128972	0.082828
5hm6aC_t1C1	-525.526139	-525.560662	-524.663164	-524.823440	-524.922156	0.129616	0.086025

5hm6aC_t6C1	-525.523279	-525.551710	-524.663178	-524.823057	-524.921585	0.129888	0.086497
5hm6aC_t7C3	-525.520293	-525.549438	-524.660600	-524.821117	-524.919924	0.129407	0.084631
5hm6aC_t7C1	-525.521879	-525.551130	-524.661227	-524.821145	-524.919709	0.129964	0.085999
5hm6aC_t4C5	-525.512466	-525.545200	-524.653193	-524.813562	-524.912286	0.128545	0.083128
5hm6aC_t4C2	-525.516685	-525.547151	-524.656738	-524.816394	-524.914822	0.129230	0.086041
5hm6aC_t5C2	-525.514647	-525.545259	-524.654892	-524.814686	-524.913159	0.129091	0.085799
r5hm6aC_t1C3	-524.890489	-524.924223	-524.023515	-524.182781	-524.280662	0.116471	0.072047
r5hm6aC_t1C12	-524.885933	-524.922581	-524.017816	-524.176809	-524.274642	0.116068	0.071157
r5hm6aC_t6C12	-524.898902	-524.921974	-524.031739	-524.189747	-524.287140	0.116432	0.073485
r5hm6aC_t7C3	-524.890756	-524.916409	-524.025367	-524.184227	-524.281990	0.116834	0.072648
r5hm6aC_t7C12	-524.883442	-524.913438	-524.017786	-524.176293	-524.273883	0.116601	0.072013
**5f6aC**							
5f6aC_1	-524.318854	-524.345957	-523.458403	-523.615975	-523.712947	0.105323	0.063173
5f6aC_3	-524.300903	-524.337377	-523.441160	-523.598764	-523.695680	0.104712	0.060220
5f6aC_11	-524.308909	-524.332772	-523.452270	-523.609325	-523.706043	0.104882	0.063111
5f6aC_10	-524.306407	-524.332269	-523.450043	-523.607051	-523.703741	0.104758	0.062930
5f6aC_2	-524.299111	-524.336199	-523.437641	-523.595117	-523.692014	0.104262	0.062002
r5f6aC_1	-523.659264	-523.683647	-522.798007	-522.953897	-523.049697	0.092890	0.049986
r5f6aC_3	-523.652983	-523.679464	-522.792297	-522.948077	-523.043769	0.092492	0.048644
r5f6aC_10	-523.645248	-523.670131	-522.788777	-522.944235	-523.039841	0.092261	0.049630
r5f6aC_11	-523.648093	-523.670626	-522.791101	-522.946593	-523.042218	0.092392	0.049826
r5f6aC_6	-523.650732	-523.670682	-522.793055	-522.948439	-523.043993	0.093090	0.049967
**5dhm6aC**							
5dhm6aC_conf15	-600.757338	-600.797632	-599.798213	-599.982876	-600.096569	0.134931	0.088163
5dhm6aC_conf3	-600.760000	-600.796986	-599.801610	-599.986152	-600.099786	0.135141	0.089224
5dhm6aC_conf5	-600.759609	-600.797690	-599.801133	-599.985261	-600.098686	0.135477	0.089962
5dhm6aC_conf4	-600.759253	-600.796196	-599.800979	-599.985607	-600.099285	0.135041	0.089058
5dhm6aC_conf5_2	-600.731876	-600.782684	-599.771330	-599.955747	-600.069252	0.134132	0.087671
5dhm6aC_conf4_2	-600.735857	-600.781542	-599.775109	-599.959625	-600.073206	0.134055	0.088243
5dhm6aC_conf2_2	-600.735549	-600.781153	-599.774894	-599.959478	-600.073075	0.134008	0.088104
5dhm6aC_conf15_2	-600.732694	-600.781560	-599.771294	-599.956074	-600.069766	0.133965	0.086953
5dhm6aC_conf15	-600.757338	-600.797632	-599.798213	-599.982876	-600.096569	0.134931	0.088163
r5dhm6aC_conf12_3	-600.159309	-600.190968	-599.194794	-599.378260	-599.491158	0.121638	0.075861
r5dhm6aC_conf12_4	-600.147960	-600.184738	-599.183790	-599.366892	-599.479607	0.122602	0.075652
r5dhm6aC_conf12_5	-600.145404	-600.186249	-599.176288	-599.360129	-599.473188	0.121306	0.074748
r5dhm6aC_conf16	-600.128445	-600.164756	-599.163180	-599.346775	-599.459662	0.121898	0.074430
r5dhm6aC_conf12_7	-600.116550	-600.147795	-599.153958	-599.337093	-599.449698	0.121390	0.074631
r5dhm6aC_conf12_8	-600.114078	-600.147017	-599.151564	-599.334723	-599.447333	0.121212	0.074327
r5dhm6aC_conf12_6	-600.092387	-600.146998	-599.126033	-599.309082	-599.421603	0.121437	0.074156
r5dhm6aC_conf12_2	-600.096512	-600.148008	-599.126632	-599.310483	-599.423335	0.120396	0.072949

[a] Using gas phase optimized (U)B3LYP-D3/6-31+G(d,p) geometries. [b] Excluding standard state correction of $\Delta G_{0K\to298K}^{1atm\to1M}$= +7.91 kJ mol^{-1}.
[c] Using geometries optimized in water at the SMD(H2O)/(U)B3LYP-D3/6-31+G(d,p) level of theory.

Table S5: Energies for gas phase optimized conformers and tautomers for modified uracil bases shown in **Figure S1** calculated at the (U)B3LYP-D3\6-31+G(d,p) level of theory.

**compound**	E_{tot}[a] (UB3LYP-D3/ 6-31+G(d,p))	E_{tot}[a] (SMD(H2O)/ UB3LYP-D3/ 6-31+G(d,p))	E_{tot}[a] (DLPNO-CCSD(T)/ cc-pVTZ)	E_{tot}[a] (DLPNO-CCSD(T)/ cc-pVQZ)	E_{tot}[a] (DLPNO-CCSD(T)/ CBS)	**corr. ΔH**[a] (UB3LYP-D3/ 6-31+G(d,p))	**corr. ΔG**[a,b] (UB3LYP-D3/ 6-31+G(d,p))
	**[Hartree]**					**[Hartree]**	
**5hmU**							
5hmU14_13	-529.400035	-529.429121	-528.537808	-528.699326	-528.798848	0.130030	0.086706
5hmU14_65	-529.393433	-529.428076	-528.531831	-528.693397	-528.792935	0.129732	0.085632
5hmU14_161	-529.396957	-529.426732	-528.535166	-528.696461	-528.795953	0.129731	0.085599
5hmU14_100	-529.398088	-529.426436	-528.535774	-528.697417	-528.797066	0.129678	0.085514
5hmU15_40	-529.373652	-529.411782	-528.513426	-528.675130	-528.774646	0.129305	0.085381
5hmU15_1	-529.377128	-529.412084	-528.516307	-528.677898	-528.777366	0.129490	0.086113
5hmU16_105	-529.373789	-529.410555	-528.513147	-528.674986	-528.774543	0.129261	0.084952
5hmU16_100	-529.375376	-529.410059	-528.515191	-528.676491	-528.775879	0.129309	0.085276
5hmU24_13	-529.381979	-529.409213	-528.521793	-528.683090	-528.782458	0.129578	0.086524
5hmU24_10	-529.375930	-529.408038	-528.516140	-528.677488	-528.776868	0.129355	0.085547
r5hmU14_161_1	-528.759604	-528.786655	-527.892800	-528.052813	-528.151351	0.116237	0.072225
r5hmU14_13_1	-528.765393	-528.786767	-527.898453	-528.057937	-528.156232	0.116314	0.073172
r5hmU14_65_1	-528.755239	-528.784744	-527.889273	-528.049265	-528.147771	0.116034	0.070608
r5hmU24_13_2	-528.749668	-528.769252	-527.884431	-528.043804	-528.142016	0.115879	0.073089
r5hmU24_100_1	-528.741697	-528.767781	-527.876696	-528.036553	-528.134956	0.115766	0.072033
r5hmU24_10_1	-528.739782	-528.766271	-527.875437	-528.035224	-528.133571	0.115663	0.071540
**5fU**							
5fU_C2	-528.178344	-528.213531	-527.321038	-527.479377	-527.576872	0.105227	0.062392
5fU_C1	-528.187414	-528.214054	-527.328583	-527.486935	-527.584546	0.105561	0.063042
5fU_t4C3	-528.167937	-528.203124	-527.310608	-527.469344	-527.566928	0.104978	0.063353
5fU_t4C2	-528.161239	-528.197588	-527.304844	-527.463293	-527.560750	0.104947	0.062495
5fU_t5C2	-528.171539	-528.197992	-527.313388	-527.471494	-527.568855	0.104440	0.063053
5fU_t4C1	-528.167371	-528.197469	-527.309731	-527.468221	-527.565805	0.105131	0.062749
5fU_t6C6	-528.168716	-528.193354	-527.315399	-527.473476	-527.570775	0.105106	0.063992
5fU_t6C3	-528.169149	-528.193134	-527.315842	-527.473885	-527.571180	0.105098	0.063996
5fU_t3C4	-528.161384	-528.194117	-527.305516	-527.463670	-527.561040	0.104797	0.062243
5fU_t3C1	-528.168156	-528.194153	-527.311083	-527.469228	-527.566693	0.105059	0.062675
r5fU_C2	-527.529324	-527.556251	-526.670896	-526.827489	-526.923812	0.093132	0.049952
r5fU_C1	-527.528514	-527.553849	-526.669687	-526.826272	-526.922632	0.093017	0.049496
r5fU_t4C3	-527.510405	-527.539760	-526.652590	-526.809585	-526.905987	0.092707	0.050267
r5fU_t4C2	-527.511156	-527.539753	-526.653598	-526.810254	-526.906504	0.092811	0.049924
r5fU_t4C1	-527.510240	-527.537722	-526.652396	-526.809055	-526.905352	0.092662	0.049520
**5dhmU**							
5dhmU_318	-604.632881	-604.668104	-603.674883	-603.860540	-603.974920	0.135745	0.090246
5dhmU_324	-604.633019	-604.667008	-603.675326	-603.860971	-603.975337	0.135587	0.090121
5dhmU_346	-604.629960	-604.666043	-603.672212	-603.857841	-603.972246	0.135493	0.089352
5dhmU_323	-604.633038	-604.663989	-603.674866	-603.860760	-603.975298	0.135362	0.089576
5dhmU_425	-604.607905	-604.650032	-603.651369	-603.837024	-603.951300	0.135026	0.089337
5dhmU_446	-604.609730	-604.649525	-603.653446	-603.839229	-603.953627	0.135043	0.089069
5dhmU_4110	-604.609408	-604.649672	-603.653058	-603.838813	-603.953121	0.135055	0.089484
5dhmU_432	-604.606883	-604.648646	-603.651311	-603.836942	-603.951234	0.134856	0.088866
5dhmU_421	-604.609076	-604.646608	-603.652376	-603.838315	-603.952783	0.134803	0.088962
5dhmU_436	-604.607693	-604.644741	-603.651465	-603.837198	-603.951556	0.134635	0.088592
r5dhmU_339	-604.005491	-604.028648	-603.041322	-603.225277	-603.338622	0.121475	0.075753
r5dhmU_438	-603.970031	-604.002677	-603.007361	-603.191704	-603.305084	0.121134	0.074661
**5hm6aU**							
5hm6aU_t1C2	-545.408329	-545.434209	-544.537975	-544.704213	-544.806594	0.117497	0.074258
5hm6aU_t1C3	-545.405753	-545.432307	-544.535674	-544.702217	-544.804738	0.116993	0.072570
5hm6aU_t7C3	-545.384037	-545.417680	-544.515524	-544.681966	-544.784353	0.117036	0.073119

5hm6aU_t7C1	-545.385741	-545.417860	-544.517038	-544.683331	-544.785669	0.116994	0.073717
5hm6aU_t7C4	-545.384717	-545.416130	-544.516511	-544.683060	-544.785505	0.116630	0.072337
5hm6aU_t6C2	-545.383099	-545.415495	-544.515558	-544.682043	-544.784435	0.117038	0.073863
r5hm6aU_t1C22	-544.782430	-544.802526	-543.906311	-544.070584	-544.171763	0.104477	0.061742
r5hm6aU_t1C3	-544.779544	-544.801629	-543.903690	-544.068476	-544.169908	0.104638	0.061175
r5hm6aU_t1C2	-544.764180	-544.795599	-543.889118	-544.053633	-544.154870	0.104071	0.060018
r5hm6aU_t7C32	-544.755964	-544.783052	-543.882653	-544.047603	-544.148978	0.104121	0.060703
r5hm6aU_t6C2	-544.757946	-544.783497	-543.884513	-544.048925	-544.150065	0.103779	0.061266
**5f6aU**							
5f6aU_C2	-544.182799	-544.210305	-543.317790	-543.480869	-543.581237	0.092709	0.049757
5f6aU_C1	-544.183707	-544.209327	-543.317794	-543.480941	-543.581342	0.092854	0.050056
5f6aU_t4C4	-544.172706	-544.200322	-543.307850	-543.471255	-543.571675	0.092564	0.051089
5f6aU_t4C1	-544.166487	-544.195870	-543.302600	-543.465752	-543.566079	0.092492	0.050048
5f6aU_t4C2	-544.164265	-544.194110	-543.299697	-543.462980	-543.563382	0.092462	0.049835
5f6aU_t6C2	-544.153483	-544.189376	-543.291937	-543.455226	-543.555596	0.092129	0.047509
5f6aU_t6C3	-544.156057	-544.188991	-543.293711	-543.457121	-543.557556	0.092350	0.049018
5f6aU_t4C3	-544.150859	-544.188831	-543.286608	-543.450131	-543.550541	0.091872	0.048684
5f6aU_t6C1	-544.137646	-544.184424	-543.277071	-543.440673	-543.541110	0.091535	0.047547
5f6aU_t6C4	-544.140578	-544.184898	-543.279307	-543.443041	-543.543548	0.091670	0.046662
r5f6aU_C1_2	-543.536202	-543.553691	-542.668143	-542.829892	-542.929300	0.080168	0.036462
r5f6aU_C2	-543.528383	-543.550420	-542.662311	-542.823643	-542.922753	0.080361	0.036807
r5f6aU_t4C1	-543.511162	-543.535594	-542.646176	-542.807621	-542.906741	0.080133	0.037138
r5f6aU_t4C4	-543.510507	-543.534936	-542.645024	-542.806870	-542.906179	0.080090	0.037794
r5f6aU_t6C3	-543.510418	-543.534271	-542.644811	-542.806775	-542.906201	0.079614	0.036077
**5dhm6aU**							
5dhm6aU_9	-620.639928	-620.671931	-619.673874	-619.864205	-619.981422	0.123236	0.077912
5dhm6aU_4	-620.640256	-620.670827	-619.674506	-619.864858	-619.982074	0.123065	0.077554
5dhm6aU_15	-620.637294	-620.669870	-619.671463	-619.862118	-619.979459	0.122962	0.076688
5dhm6aU_17	-620.636028	-620.668514	-619.670279	-619.860918	-619.978263	0.122791	0.076853
5dhm6aU_21	-620.633566	-620.666620	-619.667808	-619.858509	-619.975892	0.122691	0.076890
5dhm6aU_43	-620.617054	-620.654059	-619.653049	-619.843708	-619.960965	0.122557	0.076535
5dhm6aU_49	-620.617054	-620.654059	-619.653049	-619.843708	-619.960965	0.122557	0.076535
5dhm6aU_31	-620.617840	-620.654413	-619.653480	-619.843945	-619.961131	0.122617	0.077130
5dhm6aU_37	-620.610167	-620.650096	-619.646027	-619.836727	-619.954034	0.122174	0.076308
5dhm6aU_26	-620.609892	-620.652586	-619.641643	-619.832396	-619.949737	0.122961	0.077042
r5dhmU_11	-620.026589	-620.048762	-619.054638	-619.243316	-619.359453	0.110214	0.065145
r5dhmU_4	-620.021146	-620.047457	-619.048317	-619.236937	-619.353063	0.109540	0.064504
r5dhmU_23	-620.010081	-620.040420	-619.037732	-619.226719	-619.343019	0.110029	0.063746
r5dhmU_13	-620.010081	-620.040420	-619.037732	-619.226719	-619.343019	0.110029	0.063746
r5dhmU_10	-620.008247	-620.041349	-619.032531	-619.221528	-619.337779	0.109250	0.063834
r5dhmU_26	-620.008247	-620.041349	-619.032531	-619.221528	-619.337779	0.109250	0.063835

[a] Using gas phase optimized (U)B3LYP-D3/6-31+G(d,p) geometries. [b] Excluding standard state correction of $\Delta G_{0K\rightarrow 298K}^{1atm\rightarrow 1M}$ = +7.91 kJ/mol.
[c] Using geometries optimized in water at the SMD(H2O)/(U)B3LYP-D3/6-31+G(d,p) level of theory.

References

[1] J. Hioe, M. Mosch, D. M. Smith, H. Zipse, *RSC Adv.* **2013**, *3*, 12403-12408.
[2] J. Hioe, G. Savasci, H. Brand, H. Zipse, *Chem. Eur. J.* **2011**, *17*, 3781-3789.
[3] J. Hioe, H. Zipse, *Org. Biomol. Chem.* **2010**, *8*, 3609-3617.
[4] J. Hioe, H. Zipse, *Faraday Discuss.* **2010**, *145*, 301-313.
[5] J. Hioe, H. Zipse, *Chem. Eur. J.* **2012**, *18*, 16463-16472.
[6] D. Šakić, H. Zipse, *Adv. Synth. Catal.* **2016**, *358*, 3909-3909.
[7] H. Zipse, *Radicals in Synthesis I*, 1 ed., Springer-Verlag Berlin Heidelberg, **2006**.
[8] A. D. Becke, *J. Chem. Phys.* **1993**, *98*, 5648-5652.
[9] N. S. W. Jonasson, R. Janßen, A. Menke, F. L. Zott, H. Zipse, L. J. Daumann, *ChemBioChem* **2021**, *22*, 3333-3340.
[10] S. Grimme, J. Antony, S. Ehrlich, H. Krieg, *J. Chem. Phys.* **2010**, *132*, 154104.
[11] R. Ditchfield, W. J. Hehre, J. A. Pople, *J. Chem. Phys.* **1971**, *54*, 724.
[12] R. Krishnan, J. S. Binkley, R. Seeger, J. A. Pople, *J. Chem. Phys.* **1980**, *72*, 650.
[13] A. Altun, F. Neese, G. Bistoni, *Beilstein J. Org. Chem.* **2018**, *14*, 919-929.
[14] M. Saitow, U. Becker, C. Riplinger, E. F. Valeev, F. Neese, *J. Chem. Phys.* **2017**, *146*, 164105.
[15] F. Neese, *WIREs Comput. Mol. Sci.* **2018**, *8*, e1327.
[16] S. Zhong, E. C. Barnes, G. A. Petersson, *The Journal of Chemical Physics* **2008**, *129*.
[17] A. Halkier, T. Helgaker, P. Jørgensen, W. Klopper, H. Koch, J. Olsen, A. K. Wilson, *Chemical Physics Letters* **1998**, *286*, 243-252.
[18] T. Helgaker, W. Klopper, H. Koch, J. Noga, *The Journal of Chemical Physics* **1997**, *106*, 9639-9646.
[19] A. V. Marenich, C. J. Cramer, D. G. Truhlar, *J. Phys. Chem. B* **2009**, *113*, 6378-6396.
[20] V. S. F. Muralha, R. M. Borges dos Santos, J. A. Martinho Simões, *J. Phys. Chem. A* **2004**, *108*, 936-942.
[21] L. Hu, J. Lu, J. Cheng, Q. Rao, Z. Li, H. Hou, Z. Lou, L. Zhang, W. Li, W. Gong, M. Liu, C. Sun, X. Yin, J. Li, X. Tan, P. Wang, Y. Wang, D. Fang, Q. Cui, P. Yang, C. He, H. Jiang, C. Luo, Y. Xu, *Nature* **2015**, *527*, 118-122.
[22] V. S. F. Muralha, R. M. Borges dos Santos, J. A. Martinho Simões, *The Journal of Physical Chemistry A* **2004**, *108*, 936-942.
